# Gold-like activity copper-like selectivity of heteroatomic transition metal carbides for electrocatalytic carbon dioxide reduction reaction

**DOI:** 10.1038/s41467-021-25295-y

**Published:** 2021-08-20

**Authors:** Mohammadreza Esmaeilirad, Artem Baskin, Alireza Kondori, Ana Sanz-Matias, Jin Qian, Boao Song, Mahmoud Tamadoni Saray, Kamil Kucuk, Andres Ruiz Belmonte, Pablo Navarro Munoz Delgado, Junwon Park, Rahman Azari, Carlo U. Segre, Reza Shahbazian-Yassar, David Prendergast, Mohammad Asadi

**Affiliations:** 1 Department of Chemical and Biological Engineering, Illinois Institute of Technology, Chicago, IL USA; 2 Molecular Foundry, Lawrence Berkeley National Laboratory, Berkeley, CA USA; 3 Department of Mechanical and Industrial Engineering, University of Illinois at Chicago, Chicago, IL USA; 4 Department of Physics & CSRRI, Illinois Institute of Technology, Chicago, IL USA; 5 Department of Architecture, Pennsylvania State University, University Park, PA USA

**Keywords:** Heterogeneous catalysis, Electrocatalysis, Electrocatalysis

## Abstract

An overarching challenge of the electrochemical carbon dioxide reduction reaction (eCO_2_RR) is finding an earth-abundant, highly active catalyst that selectively produces hydrocarbons at relatively low overpotentials. Here, we report the eCO_2_RR performance of two-dimensional transition metal carbide class of materials. Our results indicate a maximum methane (CH_4_) current density of −421.63 mA/cm^2^ and a CH_4_ faradic efficiency of 82.7% ± 2% for di-tungsten carbide (W_2_C) nanoflakes in a hybrid electrolyte of 3 M potassium hydroxide and 2 M choline-chloride. Powered by a triple junction photovoltaic cell, we demonstrate a flow electrolyzer that uses humidified CO_2_ to produce CH_4_ in a 700-h process under one sun illumination with a CO_2_RR energy efficiency of about 62.3% and a solar-to-fuel efficiency of 20.7%. Density functional theory calculations reveal that dissociation of water, chemisorption of CO_2_ and cleavage of the C-O bond—the most energy consuming elementary steps in other catalysts such as copper—become nearly spontaneous at the W_2_C surface. This results in instantaneous formation of adsorbed CO—an important reaction intermediate—and an unlimited source of protons near the tungsten surface sites that are the main reasons for the observed superior activity, selectivity, and small potential.

## Abstract (web summary)

It is of high interests to develop new catalysts for selective CO2 electroreduction. Here the authors investigate two-dimensional transition metal carbides for CO2 to methane conversion with superior activity, selectivity and low overpotentials.

## Introduction

The electrocatalytic carbon dioxide reduction reaction (eCO_2_RR) driven by renewable energy has great potential for the sustainable production of chemicals and fuels at the gigaton scale that can be used any time, any place^1–4^. It also offers a promising way to store energy in chemical bonds due to having nearly two orders of magnitude higher energy density compared to the most advanced battery technologies^5^. However, reducing CO_2_ to value-added chemicals is both costly and slow based on intrinsic thermodynamics and kinetics, making the goal of an effective and feasible process a real challenge^6–9^.

Conventional pure metal catalysts such as gold (Au), palladium (Pd), silver (Ag), and newly developed transition metal dichalcogenides (TMDCs)^8,10–18^ are known to exhibit high activities for the CO_2_RR in different electrolyte solutions^19–26^. However, these catalysts are mainly selective for carbon monoxide (CO), known as an intermediate product^8,27^. Other catalysts such as copper (Cu) and Cu-based catalysts have the ability to reduce CO_2_ to various chemicals such as methane (CH_4_), ethylene (C_2_H_4_), formic acid (HCOOH), methanol (CH_3_OH), and ethanol (C_2_H_5_OH)^28–36^. Despite their good selectivity, these catalysts require high potentials—excess energy—to achieve suitable current densities—reaction rates—impeding their use for effective production of chemicals and fuels^37,38^. Therefore, an effective catalyst needs to be developed to selectively produce hydrocarbons at high rates at relatively low potentials.

Heteroatomic transition metal carbide (TMC) catalysts, also known as MXenes, have recently received great attention for various electrocatalytic reactions due to their unique structural and electronic properties^39–42^. In particular, M_2_C (M denotes transition metals) stoichiometry of this class of two-dimensional materials forms layered structures of M-C-M where a plane of carbon atoms is sandwiched between two hexagonal planes of metal atoms. This structure provides a high density of active metal atoms at the surface breaking conventional scaling relationships that limit the electrocatalytic performance of their counterparts such as TMDCs and pure metals^43^. However, there is limited knowledge of their performance and characteristics as eCO_2_RR catalysts under actual experimental conditions.

In this work, we investigate the performance of di-tungsten carbide (W_2_C), di-molybdenum carbide (Mo_2_C), diniobium carbide (Nb_2_C), and divanadium carbide (V_2_C) nanoflakes (NFs) as inexpensive, non-precious members of TMCs for eCO_2_RR. Our electrochemical results indicate that W_2_C NFs work remarkably well for eCO_2_RR by achieving a maximum CH_4_ formation current density of −421.63 mA/cm^2^ and faradaic efficiency of 82.7% ± 2 that are the highest values yet reported. These results suggest a catalytic activity higher than Au, product selectivity similar to Cu in the CO_2_RR for W_2_C NFs. Our DFT calculations also reveal that dissociation of water, chemisorption of CO_2_, and cleavage of the C–O bond, known as the most energy-consuming elementary steps in other catalysts, become nearly spontaneous at the W_2_C surface and are the main reason for the observed superior activity, selectivity, and small overpotential for CH_4_ production.

## Results and discussion

The TMC NFs i.e., W_2_C, Mo_2_C, Nb_2_C, and V_2_C were synthesized using a carburization process followed by the liquid exfoliation technique (Supplementary section 1)^27,44,45^. The electrocatalytic performance of TMC NFs with similar crystallite sizes (25.4 ± 5 nm) were then studied in a three-electrode cell and compared with Au and Cu nanoparticles (NPs), conventional catalysts for this reaction,^46^ under identical experimental conditions (Supplementary section 2). To improve the CO_2_RR performance in competing with hydrogen evolution reaction (HER), we have employed a mixture of 3 M potassium hydroxide (KOH) and 2 M choline chloride (CC) solution (KOH:CC 3 M:2 M) as the electrolyte in this study^47^.

**Fig. 1:**
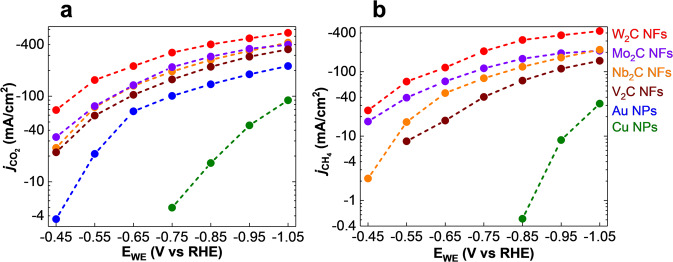
Electrocatalytic performance of TMCs i.e., W_2_C, Mo_2_C, Nb_2_C, V_2_C NFs in the two-compartment three-electrode electrochemical cell using CO_2_ saturated KOH:CC (3 M:2 M) electrolyte. **a** CO_2_ reduction reaction current densities (*j*_CO2_) of TMCs compared to Au and Cu NPs at different cathodic potentials (E_WE_) under identical experimental condition. **b** CH_4_ formation current densities (*j*_CH4_) of TMCs compared to Cu NPs at different potentials (E_WE_) under identical experimental condition.

The linear sweep voltammetry (LSV) experiments and a real-time product stream analysis show that CO_2_RR on the W_2_C surface starts at a potential of −122.7 mV vs reversible hydrogen electrode (RHE) by producing CO and H_2_ and reach maximum CO_2_RR current density (*j*_CO2RR_) of −548.9 mA/cm^2^ at −1.05 V vs RHE (Supplementary Figs. 2–4 and Fig. 1a). As shown in Fig. 1a, *j*_CO2RR_ of −419.9, −381.9, and −350.8 mA/cm^2^ were observed for Mo_2_C, Nb_2_C, and V_2_C NFs, respectively, at this potential (Supplementary section 3). However, Au and Cu NPs exhibit a *j*_CO2RR_ of −208.11 and −89.53 mA/cm^2^ at −1.05 V vs RHE (Fig. 1a). The selectivity analysis also indicates that TMC NFs produce hydrocarbons (i.e., CH_4_, C_2_H_4_, CH_3_OH, and C_2_H_5_OH) at a potential range of −0.45 to −1.05 V vs RHE for W_2_C, Mo_2_C, and Nb_2_C NFs and a potential range of −0.55 to −1.05 V vs RHE for V_2_C NFs where CH_4_ is identified as the main product (Supplementary section 3).

Figure 1b illustrates CH_4_ formation current densities (*j*_CH4_, mA/cm^2^) of the TMC NFs compared to Cu NPs, a conventional catalyst for hydrocarbon production. The partial current densities of different products (i.e., H_2_, CO, CH_4_, C_2_H_4_, CH_3_OH, and C_2_H_5_OH) were calculated by multiplying FEs and total current densities at different potentials (Supplementary section 3 and Supplementary Fig. 3). As shown in Fig. 1b, a maximum *j*_CH4_ of −421.63 mA/cm^2^ is obtained for W_2_C NFs at a potential of −1.05 V vs RHE where Nb_2_C NFs, Mo_2_C NFs, and V_2_C NFs show values of −219.16, −211.33, and −147.56 mA/cm^2^, respectively, at this potential. We also compared the CH_4_ formation activity of TMCs i.e., W_2_C, Nb_2_C, Mo_2_C, and V_2_C NFs with state-of-the-art catalysts in the literature by calculating their maximum CH_4_ formation current densities (*j*_max.CH4_, Supplementary Table 2)^46,48–54^. Supplementary Table 2b indicates that the *j*_max.CH4_ of W_2_C NFs is 3.6 and 4.2 times higher than recently studied La_2_CuO_4_ (−117 mA/cm^2^ at −1.4 V vs RHE)^51^ and Cu–N (−100 mA/cm^2^ at −1.0 V vs RHE)^48^, respectively. The partial current densities of other hydrocarbon products i.e., C_2_H_4_, CH_3_OH, and C_2_H_5_OH are also shown in Supplementary Fig. 3 (Supplementary section 3).

To evaluate the intrinsic activity of W_2_C NFs, we measured CH_4_ formation turnover frequency (TOF_CH4_) by normalizing its activity to the number of active atoms at the surface using the roughness factor method and compared it with the other catalysts in this study (Supplementary section 5). Our calculations indicate a TOF_CH4_ of 10.42 s^−1^ at a potential of −1.05 V vs RHE for W_2_C NFs; by comparison, TOF_CH4_ of 4.54, 3.74, and 2.79 s^−1^ were calculated for Mo_2_C NFs, Nb_2_C NFs, and V_2_C NFs, respectively. The calculated TOF_CH4_ of W_2_C NFs at the potential of −1.05 V vs RHE is about two orders of magnitude higher than that of Cu NPs (0.0736 s^−1^) under identical experimental conditions (Supplementary Fig. 7a). Moreover, total CO_2_RR turnover frequencies (TOF_CO2RR_) of 19.09, 19.36, 17.82, and 17.55 s^−1^ were calculated for W_2_C NFs, Mo_2_C NFs, Nb_2_C NFs, and V_2_C NFs, respectively, where Au NPs and Cu NPs exhibit TOF_CO2RR_ of 4.35 and 0.1956 s^−1^, respectively (Supplementary Fig. 7f). These results suggest the superior CH_4_ selectivity of TMC catalysts compared to state-of-the-art catalysts^48–51,54–57^.

Furthermore, we performed a comparative mechanistic study by calculating Tafel slopes for different products to gain insight about the eCO_2_RR mechanism of the TMCs i.e., W_2_C, Mo_2_C, Nb_2_C, and V_2_C NFs in the two-compartment three-electrode electrochemical cell (Supplementary section 6 and Supplementary Fig. 8)^58^. Our Tafel plot analyses show that the TMC NFs possess steeper Tafel slopes, and therefore a weaker potential dependence compared with Cu NPs for the formed products (i.e., CO, CH_4_, and C_2_H_4_) (Supplementary Fig. 8)^58^. The Tafel plot analyses suggest a different CO_2_RR mechanism for TMC NFs than that of Cu catalysts where C–O bond scission is the rate-determining step^58^.

To gain more insight to the remarkable performance of these catalysts for electrocatalytic CO_2_RR, the structural and physicochemical properties of TMC NFs were characterized at molecular and atomic scales by performing X-ray diffraction (XRD), X-ray photoelectron spectroscopy (XPS), and scanning transmission electron microscopy (STEM) (Supplementary sections 7–9). At first, we have performed XPS experiments to analyze the surface chemistry of TMC NFs. XPS analysis (Supplementary Fig. 9) indicates that our NF samples contain metallic TMCs, with little or no evident surface oxidation. The results show that the chemical composition of the surface, the empirical formula of M_2_C (M: transition metal, C: Carbide), and the oxidation state of +2 for the transition metals i.e., W, Mo, Nb, and V are similar in all synthesized TMCs (Supplementary section 7). The lattice structure and crystallite size of the TMC NFs were then studied by performing XRD experiments. The XRD pattern of W_2_C NFs shows a sharp peak at 39.91° along with three pronounced peaks at 34.84°, 38.54°, and 52.65° corresponding to (101), (100), (002), and (102) crystal surfaces of W_2_C, respectively. The XRD spectra of the TMCs show all Bragg peaks of W_2_C, Mo_2_C, Nb_2_C, and V_2_C NFs; verifying their homogenous and pure structures. The XRD results indicate a constant dominant lattice plane of (101) and a similar average crystallite size of 25.4 ± 5 nm for all synthesized TMCs (Supplementary Fig. 10)^59–61^.

**Fig. 2:**
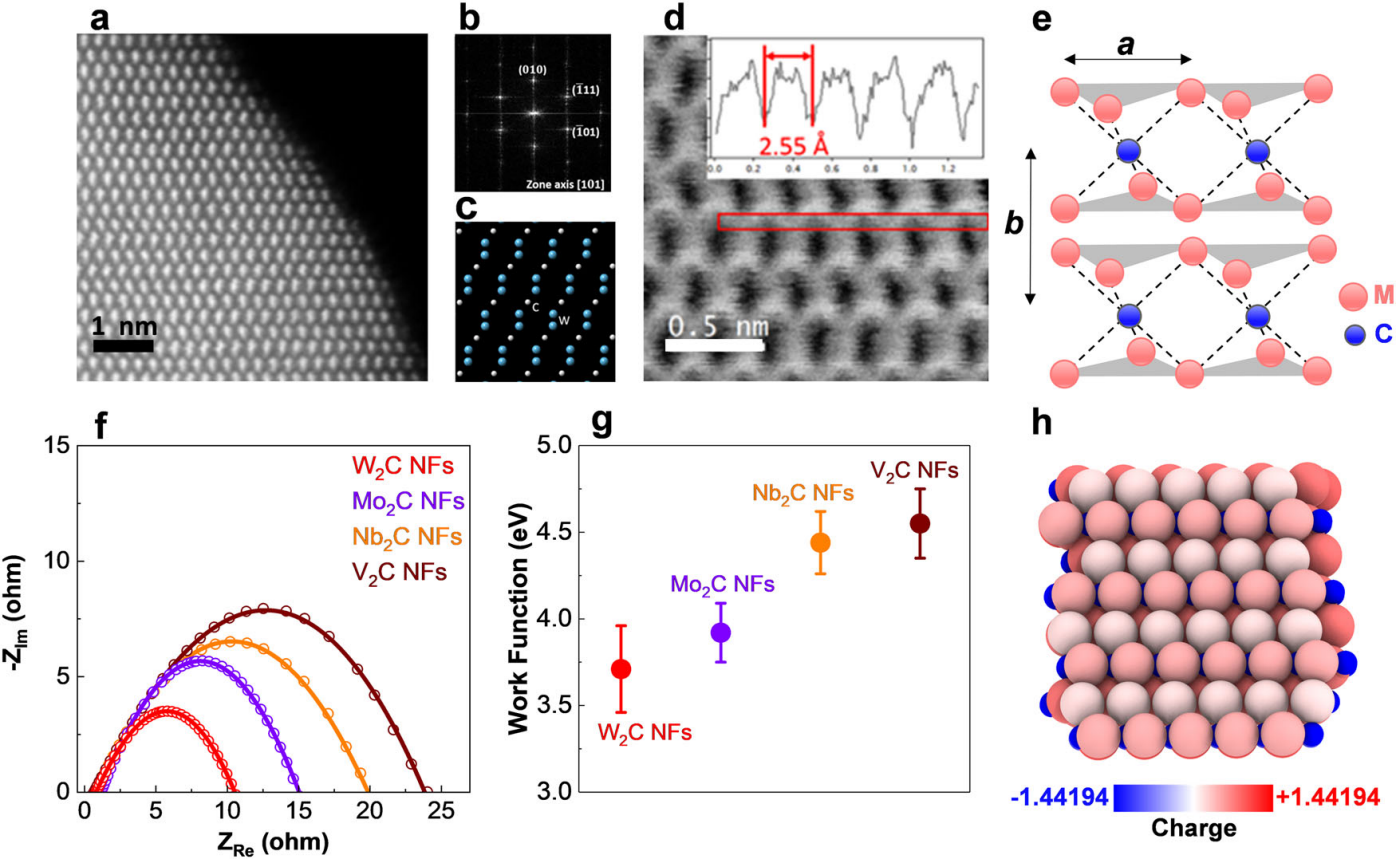
Structural and electrochemical characterization of TMC catalysts. **a** High-angle annular dark-field (HAADF) of W_2_C NFs in <101> zone axis. **b** FFT corresponding to the HAADF image of W_2_C NFs showing the diffraction spots from <101> zone axis. **c** Atomic model of W_2_C NFs in <101> zone axis. W atoms are shown as blue and carbon atoms as white spheres. **d** Bright field (BF) of W_2_C NFs in <101> zone axis. It shows the carbon atomic columns in a red box. The inset is intensity profile across red box region showing the distance between two carbon atoms is 2.55 Å. **e** Schematic of 1T structure TMCs showing tetragonal symmetry, one layer per repeat unit with octahedral coordination. The transition metal atoms (W, Mo, Nb, and V) are red and the carbon atoms are blue. The lattice constant *a* is in the range of 3.07 to 3.15 Å for synthesized TMCs. The stacking index *b* indicates the interlayer spacing which is in the range of 4.53 to 5 Å for studied TMCs. **f** Electrochemical impedance spectroscopy (EIS) for studied catalysts at a potential of −310 mV vs RHE in the two-compartment three-electrode electrochemical cell using KOH:CC (3 M:2 M) electrolyte. **g** Work function measurements for synthesized TMCs using ultraviolet photoelectron spectroscopy (UPS) method. **h** Bader charges of W_2_C NFs indicate that surface W-atoms are contributing significantly to the catalytic activity of the W_2_C (101) surface.

Furthermore, we performed atomic-scale STEM experiments to study surface atom coordination, crystallite sizes, and dominant plane structures of TMC NFs (Supplementary Figs. 11–18). Figure 2a–d shows STEM results of W_2_C NFs. Figure 2a, b indicate high-angle annular dark-field (HAADF) image and corresponding fast Fourier transforms (FFT) of W_2_C NFs in the <101> zone axis. The atomic models of the <101> zone axis and bright-field (BF) image of W_2_C NFs are represented in Fig. 2c and d, respectively. Figure 2d indicates the carbon atomic columns in the red box and the intensity profile across the red box region showing that the distance between two carbon atoms is 2.55 Å. The STEM results of other TMCs i.e., Mo_2_C, Nb_2_C, and V_2_C NFs are explained in Supplementary section 9. The STEM and XRD results of synthesized TMC NFs confirm that the structure of these materials is a perfect match with the standard 1T structure, suggesting a tetragonal symmetry and octahedral coordination of the atoms (Fig. 2e)^43,62^. Figure 2e indicates the schematic of 1T structure TMC NFs showing tetragonal symmetry, one layer per repeat unit with octahedral coordination. The lattice constant *a* is in the range of 3.07 to 3.15 Å for synthesized TMC NFs. The stacking index *b* indicates the interlayer spacing which is in the range of 4.53 to 5 Å for studied TMCs. As shown in Fig. 2e, 1T atomic coordination provides metal-terminated surface atoms that are known to be favorable binding sites of adsorbed intermediates in eCO_2_RR^43,62^. Our atomic and molecular scale structural analyses indicate that the synthesized TMC NFs have fairly similar structural properties e.g., (1T) crystalline structure with a dominant plane of (101), crystallite sizes, and atomic coordination.

To further discern the difference between the observed electrocatalytic performance of the TMCs, we have studied their electronic properties by performing electrochemical impedance spectroscopy (EIS) (Supplementary section 11) and, work function measurements using ultraviolet photoelectron spectroscopy (UPS) (Supplementary section 12)^8^. At first, we have employed the EIS experiments to compare the overall electron-transfer properties of the TMC catalysts in the double layer region (Supplementary section 11). To do this, TMC NFs with similar structural and physical properties e.g., sizes, shapes, and mass loadings (0.1 mg/cm^2^) coated on glass carbon were used as the working electrodes. This results in similar roughness, morphology, intrinsic capacitance, and exposed surface area of the studied samples confirmed by our characterization results (Supplementary sections 5–10). The EIS experiments have been performed at a potential of −310 mV vs RHE for all TMCs under identical experimental conditions (Supplementary section 11). Figure 2f shows the fitted EIS spectra of each TMC catalyst using Randles circuit model, indicating a smaller charge transfer resistance (R_ct_) for W_2_C NFs (~17 ohm) compared to the other TMCs, i.e., Mo_2_C NFs (~25 ohm), Nb_2_C NFs (~33 ohm), and V_2_C NFs (~38 ohm)^63^. The UPS method also was used to compare the surface work function of TMCs (Fig. 2g). The results indicate a lower work function for W_2_C NFs (0.2 to 0.84 eV) compared to Mo_2_C NFs (3.92 eV), Nb_2_C NFs (4.44 eV), and V_2_C NFs (4.55 eV). The charge transfer resistance obtained by EIS experiments and the surface work function value measured by UPS experiments suggests the superior activity of W_2_C NFs compared to other TMCs in this study i.e., Mo_2_C, Nb_2_C, and V_2_C NFs.

In addition to our experimental observations, we have performed density functional theory (DFT) calculations to gain more insight to the electronic and catalytic properties of M_2_C compounds. The aim is to address the enhanced activity and selectivity of these TMCs and to explore both electrochemical (i.e., driven) and chemical (i.e., favorable or spontaneous) processes that distinguish them from other catalysts, such as Au and Cu.

With respect to activity, the electronic density of states (DOS) indicate that transition metal *d* states dominate at the Fermi level of these TMCs, much more so than elemental Au, another high activity catalyst. Bader charge calculations indicate that metal atoms at the TMC surface are significantly more reduced compared to the bulk atoms (Fig. 2h and Supplementary Fig. 24). These results indicate the increased availability of electrons at metal-rich TMC surfaces, which may increase the catalytic activity of TMC NFs.

**Fig. 3:**
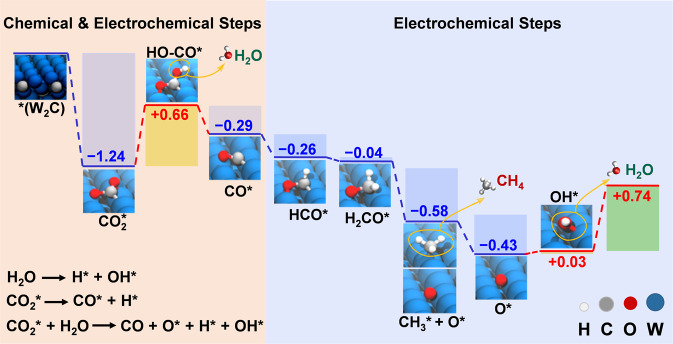
Minimum energy path for the electrochemical CO_2_ conversion into CH_4_ on the surface of W_2_C NFs. Only electrochemical steps are shown. The parts of the reaction where chemical and electrochemical steps are essential are highlighted by colors. The favorable reactions of key chemical steps are provided (for free energies of these reactions, see Table S9). The intermediates are indicated. Gibbs free energies for reaction at zero potential vs RHE are given in eV.

With respect to the increased selectivity of TMC NFs, especially for CH_4_ production, we have explored the CO_2_RR pathway on the W_2_C (101) surface in detail by using DFT calculations. Focusing initially on electrochemical processes, we employed the computational hydrogen electrode (CHE) model^64–66^ (Supplementary Tables 7–10) to explore the stepwise electronic reduction and protonation of adsorbed species in the low molecular coverage limit. The lowest free energy pathway to produce CH_4_ with only electrochemical steps is shown in Fig. 3 and Supplementary Fig. 27. The same steps with only a slight adjustment for experimental Faradaic efficiencies at the potential for optimal CH_4_ production is provided in Supplementary Fig. 26).

This pathway indicates that limiting steps (at zero potential with respect to RHE) are protonation of adsorbed CO_2_ and O, and, most importantly, the desorption of H_2_O following protonation of adsorbed OH. The difficulty of this final step is not surprising, as W_2_C (101) strongly adsorbs and spontaneously dissociates water (${{{{{{\mathrm{H}}}}}}}_{2}{{{{{\mathrm{O}}}}}}\to {{{{{{\mathrm{H}}}}}}}^{\ast }+{{{{{{{\mathrm{OH}}}}}}}}^{\ast }$, $\triangle G=-1.797\,{eV}$) without electrochemical assistance. Similarly, our calculations indicate that W_2_C (101) strongly chemisorbs CO_2_ (${{{{{{{\mathrm{CO}}}}}}}}_{2}\to {{{{{{{\mathrm{CO}}}}}}}}_{2}^{\ast },\triangle G=-1.24\,{eV}$, bond length $d\left(W-O\right)=2.04 \AA,{d}\left(W-C\right)=2.12 \AA$) in contrast to normally weak physisorption on Cu ($-0.3\,{eV}$) and other catalyst surfaces^28,67–69^. Furthermore, the (101) surface of W_2_C enables favorable and unassisted dissociation of adsorbed CO_2_ (${{{{{{{\mathrm{CO}}}}}}}}_{2}^{\ast }\to {{{{{{{\mathrm{CO}}}}}}}}^{\ast }+{{{{{{\mathrm{O}}}}}}}^{\ast }$, $\triangle G=-0.97\,{eV}$, Supplementary Table 10) suggesting that C–O bond scission may take place in the early stages of CO_2_ reduction, skipping the uphill production of adsorbed carboxyl. Based on these findings, we propose that W_2_C (101) distinguishes itself as a catalyst due to an interplay between surface-assisted chemical steps, whose energetics will depend on the local chemical equilibrium at the surface and electrochemical steps that reduce preexisting surface reagents and open up new pathways for the overall reaction to proceed. More detailed studies of such cooperative catalytic processes and their limiting steps may be encouraged based on the promise of W_2_C as a high-performance CO_2_ reduction catalyst. Here, we highlight the plausible cooperative effects of these steps, which set apart W_2_C from conventional noble metal catalysts and the other TMCs, specially for CH_4_ production. The immediate benefit of the favorable chemical processes mentioned above should be a higher surface coverage of CO_2_ (and consequently CO) and an excess of surface protons. This may explain the high Faradaic efficiencies for the production of both H_2_ and CO at low potentials (see Supplementary section 3 and Supplementary Table 1). However, once a limiting potential (−0.74 V estimate) is reached, the readily protonated products of adsorbed CO that produce CH_4_ are no longer hindered by a build-up of adsorbed byproducts (O* then OH*), which can now be protonated and released from the surface.

We can divide the complex, multistep reaction into two key parts: initial conversion of adsorbed CO_2_ to adsorbed CO, followed by conversion of adsorbed CO to CH_4_ with the release of H_2_O (see Fig. 3 and Supplementary Fig. 27). As indicated in Fig. 3, the first part, generation of adsorbed CO, can be achieved by chemical or electrochemical means. We have direct and favorable chemical conversion of adsorbed CO_2_ to adsorbed CO and O on W_2_C (101), but also two electrochemical pathways: production of HO–CO* in a single step (+0.66 eV, Fig. 3) or an alternative initially favorable protonation to OCHO* followed by two uphill electrochemical steps producing the first OCH_2_O* followed by the release of H_2_ and the final product of HO–CO* with a similar free energy cost (+0.68 eV, Supplementary Fig. 27 and Supplementary Table 10). A final electrochemically driven protonation of HO–CO* favorably releases H_2_O and leaves CO*.

With chemically or electrochemically generated adsorbed CO, we can proceed to the second part of the overall reaction to produce CH_4_ from CO_2_, which involves multiple favorable protonation steps. The W_2_C catalyst distinguishes itself here. The electrochemical activation of ${{{{{{{\mathrm{CO}}}}}}}}^{\ast }\to {{{{{{{\mathrm{HCO}}}}}}}}^{\ast }$ remains thermodynamically favorable (ΔG = −0.26 eV) on W_2_C (101), whereas on other catalysts, such as Cu, this process is usually uphill with the potential ranging from −0.74 to −0.97 V vs RHE^66,70^. Moreover, due to the spontaneous water dissociation, the direct H* transfer step ${{{{{{{\mathrm{CO}}}}}}}}^{\ast }+{{{{{{\mathrm{H}}}}}}}^{\ast }\to {{{{{{{\mathrm{HCO}}}}}}}}^{\ast }$ on W_2_C could be even more favorable with a resultant ΔG = −0.433 eV (Supplementary Table 10). The next two electrochemical steps are downhill (ΔG = −0.04 and −0.58 eV): the first forming the unstable methoxy radical ${{{{{{\mathrm{C}}}}}}{{{{{{\mathrm{H}}}}}}}_{3}{{{{{\mathrm{O}}}}}}}^{\ast }$ with oxygen attached to a surface W atom; the second leading to spontaneous dissociation into the methyl radical ${{{{{{{\mathrm{CH}}}}}}}}_{3}^{\ast }$ and a surface oxygen atom ${{{{{{\mathrm{O}}}}}}}^{\ast }$. The electrochemical conversion of the surface ${{{{{{{\mathrm{CH}}}}}}}}_{3}^{\ast }$ into CH_4_ is favorable (ΔG = −0.43 eV) and the protonation of the surface oxygen ${{{{{{\mathrm{O}}}}}}}^{\ast }$ is only slightly uphill (ΔG = +0.03 eV). As we already stated, for the overall reaction ${{{{{\mathrm{C}}}}}}{{{{{{\mathrm{O}}}}}}}_{2}^{\ast }+8{{{{{{\mathrm{H}}}}}}}^{+}/{{e}}^{-}\to \ast +2{{{{{{\mathrm{H}}}}}}}_{2}{{{{{\mathrm{O}}}}}}+{{{{{{{\mathrm{CH}}}}}}}}_{4}$ on W_2_C (101) it is the final protonation of ${{{{{{\mathrm{OH}}}}}}}^{\ast }$ to release H_2_O that is the limiting step (ΔG = +0.74 eV).

We also compared W_2_C with the other TMCs studied by calculating the energies of adsorption of water and CO_2_ as well as the potentials of the rate-determining step (i.e., protonation of ${{{{{{{\mathrm{OH}}}}}}}}^{\ast }$) for Nb_2_C, Mo_2_C, and V_2_C (Supplementary Table 11). Our calculations indicate that these TMCs also strongly chemisorb CO_2_ with adsorption energies of −1.32, −1.62, and −0.96 eV, respectively. Moreover, Nb_2_C also shows favorable C–O bond scission of adsorbed CO_2_. Additionally, Nb_2_C, Mo_2_C, and V_2_C strongly adsorb water with the energies of −1.87, −1.23, and −0.59 eV, respectively, where Nb_2_C is the only other catalyst that dissociates water. In contrast to W_2_C, the energies required for the protonation of ${{{{{{{\mathrm{OH}}}}}}}}^{\ast }$ are higher: +1.17, +1.25, and +0.85 eV for Nb_2_C, Mo_2_C, and V_2_C, respectively (Supplementary Table 11). Therefore, we can conclude that, within this set of four TMCs, W_2_C possesses the optimal characteristics for efficient completion of CO_2_RR: (1) sufficiently strong adsorption of CO_2_, (2) spontaneous dissociation of water, and (3) the lowest limiting potential for OH* protonation. We conclude that the performance of Nb_2_C is reduced due to its stronger water adsorption, resulting in the protonation of ${{{{{{{\mathrm{OH}}}}}}}}^{\ast }$ requiring more energy. We would expect Mo_2_C to have a lower surface coverage of protons and higher costs for the protonation of ${{{{{{{\mathrm{OH}}}}}}}}^{\ast }$. The weakest CO_2_ adsorption on V_2_C decreases its surface coverage, making it the worst TMC catalyst here, despite its relatively small limiting reaction potential of protonation of OH*.

As we mentioned before, for W_2_C the realistic network of pathways towards CH_4_ consists of a potential-dependent combination of competing chemical and electrochemical steps with the actual limiting potential being in the range from −0.483 to −0.744 V vs RHE (see the full path. Supplementary Fig. 27), which is consistent with our three-electrode electrochemical experimental results (Supplementary section 3 and Supplementary Table 1). A steeper Tafel slope for CH_4_ formation on W_2_C than other TMCs and Cu, (Supplementary Fig. 8) also indicates the competition between reactions for the active sites on the catalyst surface. Specifically, the spontaneous water dissociation on W_2_C (101) explains the ease of the HER in our nonacidic electrolyte where the source of protons is normally water. A weak potential dependence of the partial CO current and its small overpotential also originate from the interplay between chemical and electrochemical steps (see Supplementary Information for details).

**Fig. 4:**
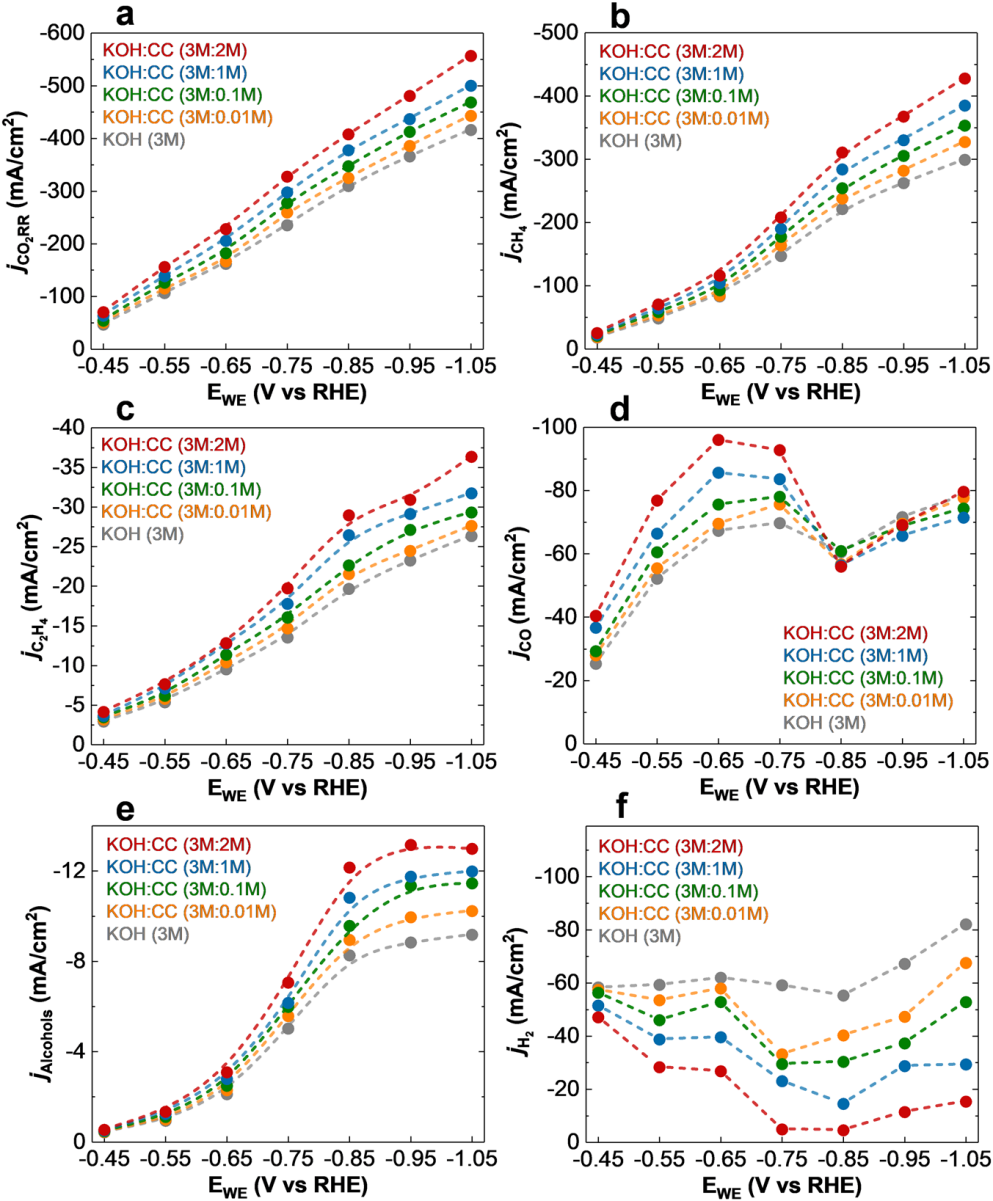
Effect of choline chloride in the electrochemical performance of W_2_C NFs for CO_2_RR. The values are measured using 3 M KOH and mixed 3 M KOH with different concentrations (0.1, 0.01, 1, and 2 M) of choline chloride (CC) electrolytes. Partial current density (*j*) measurements for **a** CO_2_RR, **b** CH_4_, **c** C_2_H_4_, **d** CO, **e** Alcohols (CH_3_OH and C_2_H_5_OH), and **f** H_2_ as a function of potential (E_WE_).

Experimentally, we have studied the effect of CC on the activity and selectivity of the TMC catalysts. To do this, we have performed electrochemical CO_2_RR in different CC concentrations of i.e., 0.01, 0.1, 1, and 2 M mixed with 3 M KOH (Supplementary section 14). Figure 4 shows CO_2_RR overall current density and different products (i.e., CH_4_, C_2_H_4_, CO, alcohols-CH_3_OH, and C_2_H_5_OH- and H_2_) partial current densities for W_2_C NFs in different CC concentration electrolytes. Figure 4a indicates that by increasing the concentration of CC in the electrolyte the CO_2_RR current density (*j*_CO2RR_) increases and reaches a maximum value of −548.89 mA/cm^2^ at a potential of −1.05 V vs RHE for 2 M of CC. The obtained value is about 32, 24, and 17, 9% higher than that of 0, 0.01, 0.1, and 1 M of CC, respectively. Moreover, a maximum CH_4_ formation current density (*j*_CH4_) of −421.63 mA/cm^2^ is obtained for 2 M CC at a potential of −1.05 V vs RHE that is about 1.41, 1.29, 1.19, and 1.1 times higher than that of 0, 0.01, 0.1, and 1 M, respectively (Fig. 4b).

The results also indicate using W_2_C NFs, maximum partial current densities of other products i.e., C_2_H_4_ (*j*_C2H4_ of −35.84 mA/cm^2^), CO (*j*_CO_ of −78.48 mA/cm^2^), and alcohols (*j*_Alcohols_ of −12.81 mA/cm^2^; −6.84 mA/cm^2^ for CH_3_OH and −5.97 mA/cm^2^ for C_2_H_5_OH) were obtained at the potential of −1.05 V vs RHE in 2 M CC (Fig. 4b–d). In contrast, the measured H_2_ partial current densities indicate that by adding a higher concentration of CC to the electrolyte solution the rate of H_2_ production decreases significantly where a minimum H_2_ formation current density of −4.48 mA/cm^2^ was obtained for 2 M CC at a potential of −0.85 V vs RHE that is 12.31, 8.97, 6.76, 3.23 times lower than that of 0, 0.01, 0.1 and 1 M CC, respectively.

These results suggest that adding CC to the 3 M KOH electrolyte suppresses the competing HER and increases the formation of CO_2_RR products more specifically CH_4_^27^.

The stability of the CC electrolytes was studied by conducting nuclear magnetic resonance (NMR) and ^13^CO_2_ isotope experiments (Supplementary sections15 and 16)^27,46,71^. The ^1^H and ^13^C NMR spectra reveal similar peak areas and chemical shifts for fresh and used electrolytes indicating no generation of new diamagnetic species or change in the CC structure under an applied potential of −1.05 V vs RHE (Supplementary Figs. 38, 39). The ^13^CO_2_ isotope experiments also show that the CO_2_ gas present inside the electrolyte is the only source of the formed products in the electrochemical CO_2_RR (Supplementary Fig. 41). These results confirm that CC with different concentrations i.e., 0, 01, 0.1, 1, and 2M remains stable at the range of applied potentials in the electrochemical CO_2_RR experiments.

Next, we studied the performance of W_2_C NFs in our developed solid polymer electrolyte flow electrolyzer for continuous electrochemical CO_2_RR using this catalyst as the cathode (Supplementary section 17). The flow electrolyzer used in this study consists of a two-compartment electrochemical setup with an active area of 5 cm^2^ coated with W_2_C NFs at the cathode and iridium oxide nanoparticles (IrO_2_ NPs) as the anode and were then fed with humidified CO_2_ and KOH:CC (3 M:2 M) electrolyte, respectively (Supplementary section 17).

**Fig. 5:**
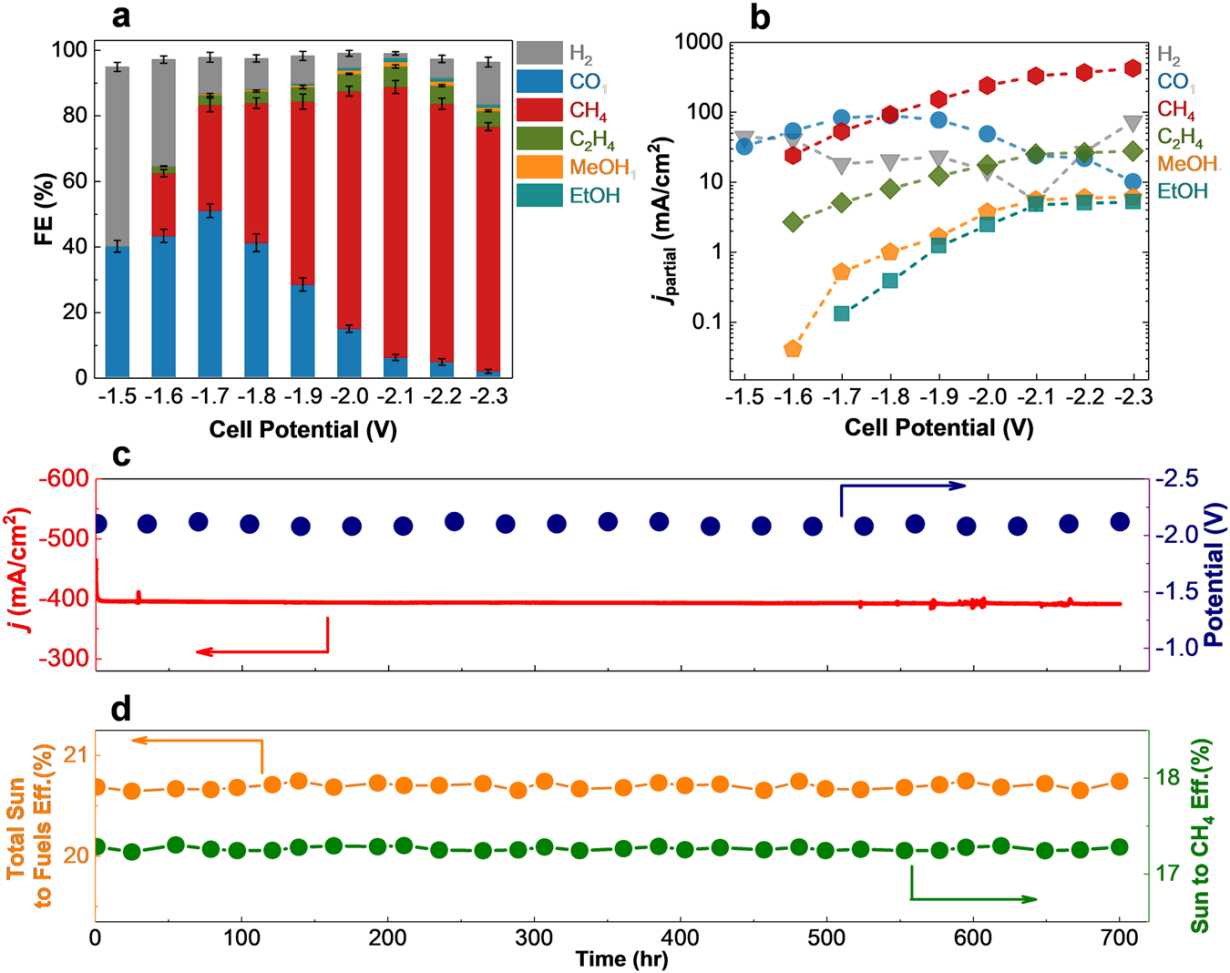
Electrocatalytic performance and stability of W_2_C NFs in the solid polymer electrolyte flow electrolyzer. **a** Faradaic efficiency (FE) measurements of H_2_, CO, CH_4_, C_2_H_4_, methanol (MeOH), and ethanol (EtOH) for W_2_C NFs at different cell potentials. The error bars represent standard deviations of four independent experiments. **b** Partial current density for each product as a function of cell potential. The values are obtained considering the total current density and faradaic efficiencies of products at the entire range of cell potential. **c** Measured total current densities and cell potentials of the solar-driven solid polymer electrolyte flow electrolyzer under one sun illumination provided by the TJ-PV cell over time. **d** Total sun to fuels efficiency and sun to CH_4_ production efficiency in the solar-driven solid polymer electrolyte flow electrolyzer over time.

To study the CO_2_RR performance of W_2_C NFs in the flow electrolyzer, we performed chronoamperometry (CA) experiments at different cell potentials ranging from −1.5 to −2.3 V for W_2_C NFs (Supplementary section 17). As shown in Fig. 5a, the results show that at a cell potential of −1.5 V, hydrogen (H_2_, FE of 54.9% ± 1.4) and CO (FE of 40.1% ± 1.8) are the dominant products. However, our measurements indicate that by increasing the cell potential a system becomes more selective for CH_4_ formation with the maximum FE of 82.7% ± 2 at a cell potential of −2.1 V. At this potential, W_2_C NFs slightly produce other products such as C_2_H_4_, CH_3_OH, C_2_H_5_OH, CO, and H_2_ with FEs of 5.6, 1.4, 1.2, 6.1, and 1.4, respectively. Figure 5b shows the maximum CH_4_, C_2_H_4_, CH_3_OH, and C_2_H_5_OH current densities of −421.28, −27.31, −5.95, and −5.19 mA/cm^2^ at the cell potential of −2.3 V, respectively, confirming high selectivity of W_2_C NFs towards CH_4_ as the main product.

Next, we coupled the electrolyzer to a triple junction photovoltaic (TJ-PV) cell with a maximum efficiency of 34.3% to determine the CO_2_RR performance and energy efficiency of W_2_C NFs in a solar-driven device (Supplementary section 18). The j-V characteristic curve of the TJ-PV cell under one sun illumination (100 mW/cm^2^) using a sun simulator light source is shown in Supplementary Fig. 48. The operating point is chosen to provide a photocurrent density of −394.3 mA/cm^2^ at a potential of −2.1 V which has the maximum FE of CH_4_ (82.7% ± 2) calculated in the flow electrolyzer (Supplementary Fig. 49).

Figure 5c shows the current density of the solar-driven electrolyzer for a 700-h continuous process at a potential of −2.1 V. The results shown in Fig. 5c indicate a negligible decrease (~2%) in the photocurrent density of W_2_C NFs over the 700-h experiment while the corresponding photo-potential fluctuates between −2.08 to −2.12 V, confirming the high stability of W_2_C NFs for CO_2_RR.

The measured sun to CO_2_RR products (CO, CH_4_, C_2_H_4_, CH_3_OH, and C_2_H_5_OH) as well as total solar-to-fuel efficiency (SFE) of W_2_C NFs over a 700-h process are shown in Fig. 5d (Supplementary section 18). As shown in this figure, an average sun to the CH_4_ production efficiency of 17.3% with negligible variation (2%) is achieved during the 700-h continuous process. Considering other products, W_2_C NFs show an SFE of 20.7%.

We also calculated the energy efficiency of CO_2_RR in our developed flow electrolyzer and compared it with state-of-the-art catalytic systems in the literature (Supplementary section 17)^29,30,37,57,72^. As shown in this figure (Supplementary Fig. 47), the maximum energy efficiency of 62.3% was obtained for our developed flow electrolyzer using W_2_C catalyst that is about 67 and 73% more efficient than Cu_oh_ (37.4%)^50^ and recently developed Cu-CIPH (36.1%)^72^ catalytic systems, respectively.

In summary, we have synthesized four members of TMCs with a formula of M_2_C, i.e., W_2_C, Mo_2_C, Nb_2_C, and V_2_C NFs using the carburization method followed by the liquid exfoliation technique and tested their catalytic performance for eCO_2_RR in KOH:CC (3 M:2 M) electrolyte. The electrocatalytic performance studies of TMCs shows these materials are mainly selective for CH_4_ formation with W_2_C NFs having the best CO_2_RR activity compared to the studied catalysts. For instance, a CO_2_RR current density of −548.89 mA/cm^2^ and a maximum CH_4_ current density of −421.63 mA/cm^2^ at the potential of −1.05 V vs RHE were observed for W_2_C NFs. Our electrochemical results also indicate that adding CC to the electrolyte enhances the formation of CO_2_RR products by suppressing the HER for all studied TMCs. Moreover, the NMR and ^13^CO_2_ isotope experiments confirm that the CC remains stable during the electrochemical experiments. Atomic and molecular scale characterizations such as XPS, XRD, and STEM indicate that all synthesized TMCs have a similar lattice structure of 1 T with a dominant plane of (101) and almost the same average crystallite size of 25.4 nm. Furthermore, the electronic property analyses of TMCs reveal superior electronic properties of W_2_C NFs: low work function; small charge transfer resistance in the electrochemical double layer region; and heavily reduced tungsten atoms at the surface, which may lead to the observed high activity. Computational results also indicate that the studied TMCs spontaneously chemisorb CO_2_ and water as compared to Cu. However, among the TMCs studied, W_2_C exhibits the optimal combination for CH_4_ production, with favorable adsorption energies of water and CO_2_ coupled with spontaneous dissociation, and less costly protonation of OH*, which is the limiting step, with a low limiting potential in the range of −0.483 to −0.744 V vs RHE. Using W_2_C NFs, we have demonstrated a solar-driven flow electrolyzer that can work up to 700 h with a solar to CH_4_ efficiency and a total SFE of 17.3 and 20.7%, respectively, under one sun illumination. The demonstrated solar-driven flow electrolyzer using a non-precious metal catalyst (W_2_C NFs) in this study achieves maximum efficiency of 62.3% making it a good candidate to approach the commercially relevant electrocatalytic CO_2_RR. This opens a new direction toward a low-cost, sustainable large-scale production of fuels from CO_2_ that can be used any time any place.

## Methods

### Synthesis of TMCs

TMCs were prepared by carburization process in a dual-zone tubular furnace with a controlled flow of CH_4_ and H_2_ mixture (volumetric ratio CH_4_:H_2_ of 1:9) at a temperature of 973 K. The obtained bulk powders were then collected and ground to fine powders in a mortar and pestle. Next, a certain amount of TMC powders were processed in isopropyl alcohol using an ultrasonic liquid processor (Sonics VibraCell VCX-130) to obtain a solution of TMC NFs. The resulting solution was further centrifuged and the top two-third of the solutions were collected and stored as the TMCs in a vial for cathode electrode preparation. A detailed explanation is provided in Supplementary section 1.

### Electrochemical setup

A two-compartment three-electrode electrochemical cell was used to perform the fundamental study for cathodic half-cell reaction using the synthesized W_2_C, Mo_2_C, Nb_2_C, and V_2_C NFs and compared them with Au and Cu NPs. In the three-electrode cell study, the working electrode was prepared by drop-casting the catalysts (mass loading of 0.1 mg) on a glassy carbon electrode with a geometric surface area of 1 cm^2^. The catalyst loading on the electrode was precisely controlled to be 0.1 mg/cm^2^ on the glassy carbon electrode. Platinum (Pt) gauze 52 mesh (Alfa Aesar) and Ag/AgCl (BASi) were used as counter and reference electrodes, respectively. The cathode and anode parts of the cell were separated through an anion exchange membrane (Sustainion X37-50 Grade RT, Dioxide Materials). All experiments were performed in a CO_2_ saturated KOH:CC (3 M:2 M) electrolyte with a pH of 14.5 ± 0.1. A two-compartment zero-gap solid polymer electrolyte flow electrolyzer was used to study the electrochemical performance where the working and counter electrodes are separated using an anion exchange membrane. Working electrodes (cathode) were prepared by brush-coating the solution of studied catalysts (W_2_C NFs, Au NPs, and Cu NPs) on the gas diffusion layer (GDL, Sigracet 39 BC, Fuel Cell Store) electrodes with a geometrical surface area of 5 cm^2^. The counter electrode (anode) was prepared using a similar procedure where IrO_2_ powder (Sigma Aldrich) was used as the catalyst solution. The actual loadings of 0.1 ± 0.01 mg/cm^2^ were determined by weighing the dry GDLs before catalyst deposition and coated GDLs after being dried in a vacuum oven overnight. As a separator in our experiments, we used an anion exchange membrane (Sustainion X37-50 Grade RT, Dioxide Materials) which was treated in 1 M KOH overnight at 75 °C and then washed with deionized water prior to use. Anolyte flow of KOH:CC (3 M:2 M) with a flow rate of 20 ml/min was fed to the anode compartment using a peristaltic pump (Masterflex, Cole-Parmer). A mass flow controller (SmartTrak 50, Sierra, calibrated with CO_2_ gas) connected to the CO_2_ humidifier kit, was used to feed the cathode compartment with a flow rate of 50 ml/min.

### PV cell characterization

A solar-powered flow cell was assembled by connecting the solid polymer electrolyte flow electrolyzer to a triple junction photovoltaic (TJ-PV) solar cell. The TJ-PV cell was characterized at different sun illuminations using a custom-made sun simulator light source and an InGaAs photodiode (Thorlabs, FDG03-CAL) with a known responsivity calibration curve. Our results indicated a maximum efficiency of 34.32% under one sun illumination used in our study.

### Electrochemical characterization

Electrochemical experiments were performed using a Biologic Potentiostat SP-150. The CA technique was used to study the performance of TMC NFs i.e., W_2_C, Mo_2_C, Nb_2_C, and V_2_C NFs and compared them with that of Au and Cu NPs. The CA experiments were carried out in the range of −0.45 to −1.05 V vs RHE potentials. All experiments were performed under identical experimental conditions. The LSV technique was used to study the fundamentals of the cathodic half-cell reaction in the three-electrode cell setup. LSV curves were obtained by sweeping the potential between +0.2 and −1.05 V vs RHE with a scan rate of 20 mV/s. The conversion of Ag/AgCl reference electrode potential to the RHE scale was calculated using the Nernst equation considering the pH of the solution (pH = 14.5).

### Product characterization

A gas chromatography system (GC, SRI, 8610 C) equipped with a flame ionization detector (FID) and a thermal conductivity detector (TCD) was used to detect and quantify the electrochemical CO_2_RR products. Ultra-high purity helium (He) and nitrogen (N_2_) gases (UHP 99.99%, Airgas) were used as the carrier gas to identify any possible type of product. the signal response of the FID and TCD to each gaseous product (e.g., H_2_, CO, CH_4_, and C_2_H_4_) was calibrated by analyzing a series of standard gas mixtures with known compositions prior to the experiments. To study the products, 1 mL sample of the headspace of the cell was injected into the GC system using a lock-in syringe (Hamilton). Moreover, an in situ differential electrochemical mass spectrometer (DEMS, Hiden Analytical, HPR-40) was used to validate the obtained information from the GC system by continuously detecting all possible products, even at trace amounts (partial pressure of 1 × 10^−13^ Torr), during the electrochemical CO_2_RR (CA experiment), resulting in more precise measurement. The signal responses of the DEMS instrument for different products (H_2_, CO, CH_4_, C_2_H_4_, CH_3_OH, and C_2_H_5_OH) were calibrated by feeding standard samples into the mass spectrometer. An electron energy of 70 eV was used for ionization of all species, with an emission current of 500 µA. All mass-selected product cations were detected by a secondary electron multiplier with a detector voltage of 1200 V for maximizing the signal-to-noise ratio of the products.

### X-ray diffraction (XRD)

The XRD technique was used to identify the phase purity and crystallinity of all studied catalysts (W_2_C, Mo_2_C, Nb_2_C, V_2_C NFs, Au NPs, and Cu NPs) using a Bruker D2 PHASER diffractometer in Bragg–Brentano geometry employing a Ni filtered Cu *K*α radiation (1.5405 Å). The XRD patterns were obtained using a LynxEye linear position-sensitive detector and a step width of 0.2 °2θ with a counting time of 1 s/step.

### X-ray photoelectron spectroscopy (XPS)

A Thermo-Scientific ESCALAB 250Xi instrument equipped with an electron flood and scanning ion gun was used to identify the oxidation states of the W_2_C NFs. All obtained spectra were analyzed using Thermo-Avantage software, considering the standard carbon peak at 284.8 eV and relative sensitivity factors.

### Ultraviolet photoelectron spectroscopy (UPS)

Surface work function measurements were carried out using the UPS technique. All UPS data were acquired by a Thermo-Scientific ESCALAB 250Xi instrument using He I (21.2 eV) ultraviolet radiation and a pass energy of 8.95 eV.

### Scanning transmission electron microscopy (STEM)

W_2_C NFs were characterized at the atomic scale using a spherical aberration-corrected JEOL JEM-ARM 200CF STEM with a cold field emission gun operating at 200 kV. HAADF detector with 22 mrd inner-detector angle and BF detector were utilized to obtain the atomic resolution images.

### Theoretical study

We performed a comparative DFT analysis for the observed catalytic activity and reactivity of W_2_C NFs with Au and other TMCs using the SIESTA package, with Perdew–Burke–Ernzerhof functional with a double-zeta with polarization (DZP) localized basis set and the norm-conserving Troullier-Martins pseudopotentials. Calculations of DOS for bulk and slab geometries of Au and TMCs were performed using the Effective Screening Method (ESM)^73^ for Brillouin zones of the unit cells sampled by Monkhorst-Pack k-point grids of size 9 × 9 × 9 and 1 × 9 × 9, respectively, together with a plane-wave cutoff of 300.0 Ry. The optimization of the atomic positions and cell parameters were carried out using a conjugate-gradient algorithm until a maximum atomic force tolerance of 0.04 eV/Å and a maximum stress component along each periodic direction of lower than 1 GPa were achieved. The Vienna ab initio Simulation Package (VASP, version 5.4.4) with PAW (projector augmented wave method) and Perdew–Burke–Ernzerhof exchange-correlation functionals were used to analyze the adsorption free energies of various molecular species on the (101) surface of M_2_C (M = W, V, Mo, Nb). All the VASP calculations were performed for neutral non-spin-polarized systems and a dipolar electrostatic correction was used along the normal to the surface of the slab. Next, we used the tetrahedron method with Blöchl corrections and 1 × 3 x 3 Monkhorst-Pack grid k-point sampling for the calculations of total electronic energy (smearing σ = 0.1). The adsorption free energies were then used within the CHE model^64–66^ to evaluate the lowest free energy pathways and the limiting reaction potentials.

## Supplementary information

Supplementary Information

## Data Availability

The data supporting the findings of this study are available within the article and its Supplementary Information. Other relevant data are available from the corresponding author upon reasonable request. The Source data underlying figures of this manuscript are provided as a Source Data file which is provided with this paper. The X-ray crystallographic coordinates for structures reported in this study have been deposited at the Cambridge Crystallographic Data Centre (CCDC), under deposition numbers 2089992–2089995. Source data are provided with this paper.

## Source data

Source Data
