# Supplementary material for: Gold-like activity copper-like selectivity of heteroatomic transition metal carbides for electrocatalytic carbon dioxide reduction reaction
Source: Nat Commun. 2021 Aug 20;12:5067. doi: 10.1038/s41467-021-25295-y (PMC8379264; doi:10.1038/s41467-021-25295-y)
Supplement: Supplementary file 1 — Supplementary Information [file 41467_2021_25295_MOESM1_ESM.pdf]

Supplementary Information

Gold-Like Activity Copper-Like Selectivity of Heteroatomic Transition Metal Carbides for Electrocatalytic Carbon Dioxide Reduction Reaction

Mohammadreza Esmaeilirad[1]*, Artem Baskin[2]*, Alireza Kondori[1], Ana Sanz Matias[2], Jin Qian[2], Boao Song[3], Mahmoud Tamadoni Saray[3], Kamil Kucuk[4], Andres Ruiz Belmonte[1], Pablo Navarro Munoz Delgado[1], Junwon Park[1], Rahman Azari[5], Carlo U. Segre[4], Reza Shahbazian-Yassar[3], David Prendergast[2†], Mohammad Asadi[1†]

[1]Department of Chemical and Biological Engineering, Illinois Institute of Technology, Chicago, IL 60616, USA.
[2]Molecular Foundry, Lawrence Berkeley National Laboratory, Berkeley, CA 94720, USA.
[3]Department of Mechanical and Industrial Engineering, University of Illinois at Chicago, Chicago, IL 60607, USA.
[4]Department of Physics & CSRRI, Illinois Institute of Technology, Chicago, IL 60616, USA.
[5]Department of Architecture, Pennsylvania State University, University Park, PA 16802, USA.
*These authors equally contributed to this work.
†Corresponding authors: dgprendergast@lbl.gov, m.asadi@iit.edu

Table of Contents

1- Catalysts Preparation

1.1 Synthesis of Transition Metal Carbide Nanoflakes (TMC NFs)

1.2 Synthesis of Copper Nanoparticles (Cu NPs)

1.3 Preparation of Gold Nanoparticles (Au NPs)

2- Two-Compartment Three-Electrode Electrochemical Cell

3- Products Analysis and Faradaic Efficiency (FE) Measurements

4- *In Situ* CO_2RR Onset Potential Measurement of W_2C NFs

5- Turnover Frequency Measurements (TOF) of TMCs

6- Tafel Analyses of TMCs

7- X-ray Photoelectron Spectroscopy (XPS) of TMCs

8- X-ray Diffraction (XRD) patterns of TMC NFs

9- Scanning Transmission Electron Microscopy (STEM) of TMCs

10- Dynamic Light Scattering (DLS) of TMCs

11- Electrochemical Impedance Spectroscopy (EIS) of TMCs

12- Work Function Measurements – UPS method

13- Theoretical Modeling and Calculations

13.1 General Methodology

13.2 Free Energy Calculations and Computational Hydrogen Electrode Model

14- The Effect of Choline Chloride on the Electrochemical Performance of TMCs

15- Stability Analysis of Choline Chloride Electrolyte using NMR experiments

16- 13-C Labeled Carbon Dioxide ($^{13}CO_2$) Experiments

17- Continuous Flow Electrolyzer Experiments: Methods and Materials

18- Electrochemical Performance of the Solar-Powered Flow Cell: Methods and Materials

1- Catalysts Preparation

1.1 Synthesis of Transition Metal Carbide Nanoflakes (TMC NFs)

Transition metal carbide nanoflakes (TMC NFs) *i.e.,* W_2C, Nb_2C, Mo_2C and V_2C NFs were synthesized using carburization method under controlled environment. A specific amount of metal oxide powders [WO_3, MO_3, V_2O_5 and Nb_2O_5] (Sigma Aldrich)] were introduced on a quartz boat and carburized with a flowing mixture of methane (CH_4) and hydrogen (H_2). The flowing rate of mixed gas was 220 ml/g, including 22 ml/g CH_4 (Airgas) and 198 ml.g H_2 (Airgas). The temperature was increased from room temperature to 373 K and from 373 K to specific value for each TMCs with rates of 100 K/h and 50 K/h, respectively. The sample was then held at final temperature for 2 hours and cooled down to room temperature in the carburizing mixed gas. Next, in a custom-made liquid exfoliation method, the bulk of the synthesized catalysts were processed with an ultrasonic liquid processor (Sonics VibraCell VCX-130) to obtain W_2C, Nb_2C, Mo_2C and V_2C NFs. Basically, 300 mg of the bulk powder was dispersed in 60 mL isopropanol using the sonication probe for 10 hrs. The solution was further centrifuged at 1000 rpm for 30 min and the top two-thirds of the solution was collected and stored in vials.

1.2 Synthesis of Copper Nanoparticles (Cu NPs)

Copper (II) nitrate trihydrate [$Cu(NO_3)_2.3H_2O$] (Sigma Aldrich), carbon black (Fuel Cell store) and sodium hydroxide [NaOH] (Sigma Aldrich) were used in the heterogeneous deposition-precipitation (DP) method to synthesis of copper nanoparticles on carbon black (Cu NPs)[1–6]. The nitrate solution of copper (Cu) was prepared by adding 50 ml of deionized water to 670 mg of Cu salt. Then DP process was conducted for 12 hours on a magnetic hotplate at 363 K. Metal ions were precipitated on 1.1 g of carbon black by adding 40 ml of 6 wt.% NaOH solution in specific time intervals to obtain different sizes of Cu nanoparticles with 16 wt.% metal loading deposited on the carbon black. The solution containing so-called Cu nanoparticles were then rinsed with deionized water, filtered and dried in a vacuum oven at 383 K for overnight. The dried powders

subsequently functionalized in a tubular furnace using a mixed hydrogen/argon flow with the H_2:Ar volume ratio of 1:20 under a pressure of 100 torr for 4 h at 773 K.

1.3 Preparation of Gold Nanoparticles (Au NPs)

Gold (Au) nanoparticles (Alfa-Aesar, 99.99%) were used as received. A specific amount of Au nanoparticles was sonicated in isopropyl alcohol using a Branson 2800 ultrasonic processor, Cleanosonic. The prepared solution was subsequently used to coat the working electrodes for electrochemical experiments.

2- Two-Compartment Three-Electrode Electrochemical Cell

As shown in Supplementary Figure 1, a custom designed two-compartment three-electrode electrochemical cell was used to perform carbon dioxide reduction reaction (CO_2RR). The catalysts were coated on a glassy carbon electrode by drop casting method[7] and employed as working electrodes. The catalysts loading on the electrode was precisely controlled to be 0.1 mg on the glassy carbon electrode with the surface area of 1 cm^2.

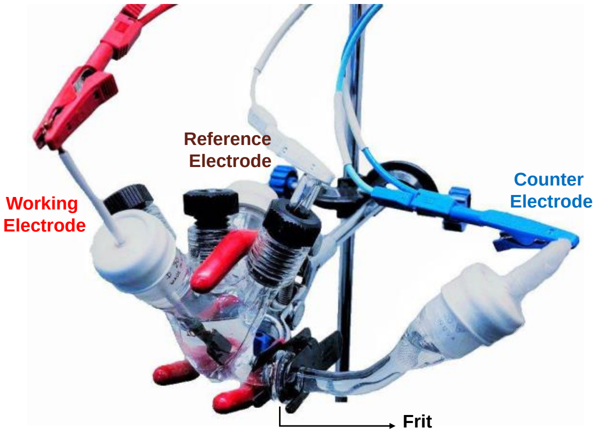

Supplementary Figure 1 | Photo of two-compartment three-electrode electrochemical cell.

Linear sweep voltammetry (LSV) experiments were performed to evaluate the overall catalytic activity of synthesized catalysts. All experiments were performed under identical electrochemical conditions in the cell with 25 ml of a mixture of 3 M potassium hydroxide (KOH) and 2 M choline-chloride (KOH:CC (3M:2M)) electrolyte saturated with CO_2 (UHP 99.99%, Airgas)[8]. Platinum (Pt) gauze 52 mesh (Alfa Aesar) and Ag/AgCl (BASi) were used as counter and reference electrodes,

respectively. The cathode and anode parts of the cell were separated through a frit to eliminate the effect of oxidation product at the anode surface.

pH of the solution before and after experiment was about 14.5±0.1 for all experiments using a Thermo Scientific Orion A111 pH meter. The cell was connected to the potentiostat (Biologic) for the electrolysis experiments. The LSV curves were obtained by sweeping a potential between +0.2 and -1.05 V vs. reversible hydrogen electrode (RHE) with scan rate of 20 mV/s. The current densities are normalized according to the geometrical surface area.

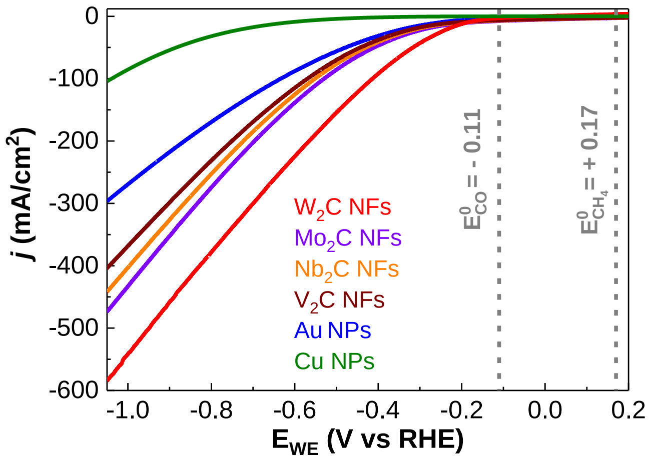

Supplementary Figure 2 | Linear sweep voltammetry (LSV) results for W_2C NFs, Mo_2C NFs, Nb_2C NFs, V_2C NFs, Au NPs, and Cu NPs with respect to RHE at a scan rate of 20 mV/s.

The calibration of Ag/AgCl electrode was performed in a the three-electrode cell with polished Pt wires as the working and counter electrodes, the Ag/AgCl electrode as the reference electrode and 1 M sulfuric acid (H_2SO_4) solution as an electrolyte. The electrolyte was pre-purged and saturated with high purity hydrogen (H_2) gas before calibration experiment. LSV experiment was then run at a scan rate of 0.5 mV/s, and the potential at which the current crossed zero was taken to be the thermodynamic potential for the hydrogen electrode reactions. The zero current point was at -0.210 V.

All potentials were converted to the RHE using Nernst equation (Equation 1) with $\mathrm{E}^0_{\mathrm{Ag/AgCl\ -RHE}} = 0.210$ and bulk pH of solution = 14.5:

$$\textit{Potential in RHE} = \textit{Applied potential vs Ag/AgCl} + 0.210\ V + 0.0592 \times pH \quad \text{(Equation 1)}$$

3- Products Analysis and Faradaic Efficiency (FE) Measurements

The products of electrocatalytic CO_2RR were detected and quantified using a gas chromatograph (GC) of type SRI 8610C Multiple Gas Analyzer and differential electrochemical mass spectroscopy (DEMS, HPR-40, Hiden Analytical). The GC system comprised a sampling loop equipped with a flame ionization detector (FID) and a thermal conductivity detector (TCD). Ultra-high purity helium (He) and nitrogen (N_2) (UHP 99.99%, Airgas) were used as the carrier gases to identify any possible type of product. The column oven was maintained at 40 ºC for 2 min followed by a temperature ramp at 20 ºC/min to 250 ºC which was held constant for 1 min for a precise product analysis. In order to quantify the CO_2RR products, the signal response of the FID and TCD to each product was calibrated by analyzing a series of standard mixtures with known compositions prior to the experiments. The chronoamperometry (CA) experiments were performed in the two-compartment three-electrode electrochemical cell for 30 min at different potentials. To analyze the products of the electrochemical experiment, 1 ml samples were taken from the dead volume (15 ml) of the cell using a lock-in syringe (Hamilton) and injected into the gas chromatograph.

The *in situ* differential electrochemical mass spectrometry (DEMS) was used in this work to validate that the electrocatalytic performance of catalysts measured by GC*. In* situ DEMS allows for the continuous detection of gaseous and volatile species produced during the electrochemical CO_2RR even at trace amounts (partial pressures as low as 1×10^{-13} Torr). The analysis system consists of a commercial mass spectrometer (HPR-40, Hiden Analytical) and a custom-made capillary as the inlet. The electrochemical CO_2 reaction products were continuously collected during the chronoamperometry experiments. Mass spectrometer settings were determined to be optimal for enhancing the signal to noise ratio of products. The signal responses of the DEMS instrument for different products were calibrated by feeding standard samples into the mass spectrometer. An electron energy of 70 eV was used for ionization of all species, with an emission current of 500 µA. The mass to charge ratios (m/z) of 2, 12, 15, 26, 31 and 46 were selected for

measuring production of H_2, CO, CH_4, C_2H_4, CH_3OH and C_2H_5OH respectively. All mass-selected product cations were detected by a secondary electron multiplier with a detector voltage of 1200 V for maximizing the signal to noise ratio of the products.

Equation 2 was used to measure FEs of catalysts at different applied potentials.

$$FE\% = \frac{experimental\ moles\ of\ product}{theoretical\ moles\ of\ product} = \frac{moles\ of\ product\ (measured\ by\ GC\ and\ DEMS)}{\frac{j\left(\frac{mA}{cm^2}\right) \times t(S)}{n \times F}} \times 100, \text{(Equation 2)}$$

Where in Equation 2, the experimental moles of products are obtained using the calibration curves. The theoretical mole of each product was also extracted by dividing the number of Coulombs by the number of electrons required (n) for formation of each product *e.g.*, H_2, CO, CH_4, C_2H_4, CH_3OH and C_2H_5OH (n_{H2}=2, n_{CO}=2, n_{CH4}=8, n_{C2H4}=12, n_{CH3OH}=6, n_{C2H5OH}=12) multiplied by Faraday's constant (96,485 C/mole) (Equation 3). FE measurements of catalysts at different potentials are presented in Supplementary Table 1.

Supplementary Table 1 | FE measurements of catalysts in the two-compartment three-electrode electrochemical cell at different potentials using KOH:CC (3M:2M).

Catalyst	Potential (mV vs RHE)	FE_{H2} (%)	FE_{CO} (%)	FE_{CH4} (%)	FE_{C2H4} (%)	FE_{MeOH} (%)	FE_{EtOH}(%)
	-122.7	98.03821	0.69473				
	-150	98.13833	1.23069				
	-250	94.75136	4.40023				
	-310	92.01354	5.07349				
W_2C NFs	-450	38.56548	33.09669	20.4983	3.39188	0.35040	0.08952
	-550	14.95612	40.59594	37.09258	4.04116	0.50832	0.19872
	-650	10.24194	36.69633	44.29114	4.90434	0.67275	0.50543
	-750	1.449726	27.39726	61.38772	5.82408	1.24858	0.83515
	-850	1.089321	13.39866	74.38512	6.93051	1.55406	1.35600
	-950	2.283232	13.79892	73.38572	6.17138	1.41386	1.21324
	-1050	2.622896	13.59914	73.08598	6.21035	1.18453	1.03392
	-310	96.71945	2.91868				
	-450	46.4846	25.14928	26.1234	0.38027	0.06386	
Mo_2C NFs	-550	29.87478	32.44245	35.59428	0.97068	0.120872	0.03352
	-650	20.39595	36.33299	41.72814	1.03072	0.19166	0.14755
	-750	7.7723	43.14587	47.61159	1.0007	0.28815	0.22775
	-850	7.68397	40.3672	50.65557	0.83058	0.40711	0.34446
	-950	8.70298	40.37661	49.31236	0.76053	0.33095	0.30734
	-1050	9.43316	39.37781	48.61174	0.62043	0.28939	0.27005
	-310	95.56138	4.98663				
	-450	53.69744	39.8302	3.84532			
	-550	22.8952	58.2648	16.61241		0.04349	

Nb_2C NFs	-650	12.5994	54.9375	30.18055	0.713562	0.10097	0.04714
	-750	8.3996	51.3644	36.20461	1.156693	0.20974	0.05271
	-850	5.7804	48.6891	41.22841	1.455176	0.27373	0.11345
	-950	5.8696	45.2095	45.37916	1.798176	0.29049	0.12198
	-1050	4.3396	43.4630	47.59129	1.923171	0.29421	0.15418
V_2C NFs	-310	98.03052	1.21974				
	-450	54.9035	42.2965				
	-550	33.13971	55.9612	9.0990		0.0275	
	-650	25.26626	60.3895	12.3391	0.6050	0.0858	0.0227
	-750	20.38106	57.2617	20.2890	0.7681	0.1848	0.0321
	-850	15.72569	54.4701	27.4469	1.1572	0.2341	0.0632
	-950	10.09962	51.6829	33.3631	1.4542	0.2613	0.0654
	-1050	9.70612	49.0069	36.7280	1.5788	0.2587	0.0851
Au NPs	-450	88.8327	8.55599				
	-550	68.9924	29.75408				
	-650	35.9947	62.63814				
	-750	29.7106	68.49212				
	-850	26.8223	71.2194				
	-950	23.3358	74.09392				
	-1050	20.3238	75.78839				
Cu NPs	-750	73.32156	20.84832				
	-850	54.20106	37.13029	1.23324	0.95769		
	-950	27.55689	38.29192	12.59866	6.02783	1.8751	7.43279
	-1050	10.23211	28.06677	30.57782	13.61896	2.78253	10.89543

The partial current densities of different products were calculated by considering the FE measurements and the total current densities of synthesized catalysts over the studied range of cell potentials. Supplementary Figure 3 indicates different products partial current densities of TMCs, Au NPs and Cu NPs in the three-electrode cell.

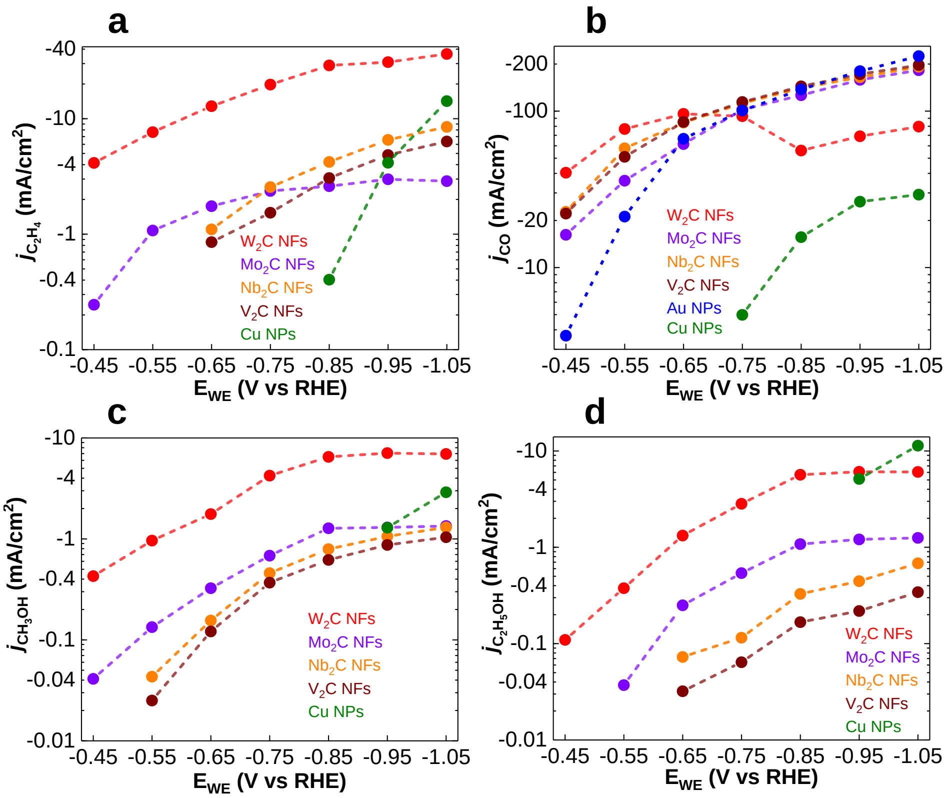

Supplementary Figure 3 | Catalytic performance of W_2C NFs, Mo_2C NFs, Nb_2C NF, V_2C NFs compared to Au NPs and Cu NPs. (a) C_2H_4 **(b)** CO, **(c)** CH_3OH and **(d)** C_2H_5OH. All experiments were performed in the two-compartment three-electrode electrochemical cell using CO_2 saturated KOH:CC (3M:2M).

We also compared the maximum CO_2RR and maximum CH_4 formation current densities of TMCs with state-of-the-arts catalysts in the literature. The data points shown in Supplementary Table 2 were extracted at the maximum CO_2RR and CH_4 formation faradaic efficiencies of our catalysts as well as others in the literature.

Supplementary Table 2 | Electrocatalytic performance of TMC NFs, *i.e.*, W_2C, Nb_2C, Mo_2C, and V_2C NFs for in CO_2RR compared to the state-of-the-art electrocatalysts in literature.

**a)** Maximum CO_2 reduction reaction current densities (j_{CO2RR})					
**Catalyst**	$\boldsymbol{j_{CO2RR}}$	**Potential (V vs RHE)**	**Electrolyte**	**pH**	**Ref.**
W_2C NFs	-548.89	-1.05	KOH:CC (3M:2M)	14.5	This study
Nb_2C NFs	-419.85	-1.05	KOH:CC (3M:2M)	14.5	This study
Mo_2C NFs	-381.86	-1.05	KOH:CC (3M:2M)	14.5	This study
V_2C NFs	-350.79	-1.05	KOH:CC (3M:2M)	14.5	This study
Cu-N	-320	-1	1M KOH	N/C	[9]
Au NPs	-208.11	-1.05	KOH:CC (3M:2M)	14.5	This study
Ni-N-C	-200	-1.25	1M $KHCO_3$	6.8	[10]
$Co_{Phthalocyanine}$	-175	-1.25	1M KOH	N/C	[11]
NGQDs	-149	-1.05	1M KOH	13.48	[12]
Cu_{oh}	-120	-0.96	1M KOH	13.78	[13]
ERD-Cu	-110	-1.4	0.5M $KHCO_3$	7.2	[14]

**b)** Maximum CH_4 formation current densities ($j_{max.\ CH4}$)						
**Catalyst**	**$j_{max.CH4}$**	**E_{WE} (V vs RHE)**		**Electrolyte**	**pH**	**Ref.**
W_2C NFs	-421.63	-1.05		KOH:CC (3M:2M)	14.5	This study
Nb_2C NFs	-219.16	-1.05		KOH:CC (3M:2M)	14.5	This study
Mo_2C NFs	-211.33	-1.05		KOH:CC (3M:2M)	14.5	This study
V_2C NFs	-147.56	-1.05		KOH:CC (3M:2M)	14.5	This study
La_2CuO_4	-117	-1.4		1M $KHCO_3$	6.8	[15]
Cu	-112.5	-1.4		1M $KHCO_3$	N/C	[16]
Cu-N	-100	-1		1M KOH	N/C	[9]
Cu_{oh}	-83	-0.96		1M KOH	13.78	[13]
OFn-Cu	-39	-1.15		1M $KHCO_3$	8	[6]
Cu NPs	-31.86	-1.05		KOH:CC (3M:2M)	14.5	This study
NGQDs	-30	-1.05		1M KOH	13.48	[12]
CuPc	-13	-1.06		0.5M $KHCO_3$	N/C	[17]
n-Cu/C	-10.2	-1.45		0.5M $NaHCO_3$	6.8	[18]
**c)** CH_4 formation current densities (j_{CH4}) at CH_4 onset potentials						
**Catalyst**	**j_{CH4}**	**E_{WE} (V vs RHE)**	**Overpotential (V)**	**Electrolyte**	**pH**	**Ref.**
W_2C NFs	-25.02	-0.45	0.62	KOH:CC (3M:2M)	14.5	This study
Nb_2C NFs	-2.19	-0.45	0.62	KOH:CC (3M:2M)	14.5	This study
Mo_2C NFs	-23.25	-0.45	0.62	KOH:CC (3M:2M)	14.5	This study
V_2C NFs	-8.3	-0.55	0.72	KOH:CC (3M:2M)	14.5	This study
La_2CuO_4	-9	-0.7	0.87	1M $KHCO_3$	6.8	[15]
Cu	-2.5	-0.8	0.97	1M $KHCO_3$	N/C	[16]
Cu-N	-1	-0.6	0.77	1M KOH	N/C	[9]
Cu_{oh}	-4	-0.65	0.82	1M KOH	13.78	[13]
OFn-Cu	-0.45	-0.85	1.02	1M $KHCO_3$	8	[6]
Cu NPs	-0.52	-0.85	1.02	KOH:CC (3M:2M)	14.5	This study
NGQDs	-1.5	-0.45	0.62	1M KOH	13.48	[12]
CuPc	-1	-0.86	1.03	0.5M $KHCO_3$	N/C	[17]
n-Cu/C	-0.5	-0.95	1.12	0.5M $NaHCO_3$	6.8	[18]

4- *In Situ* CO_2RR Onset Potential Measurement of W_2C NFs

In situ differential electrochemical mass spectrometry (DEMS) was used to measure the starting potential of CO_2RR. The potential was swept between +110 to -250 mV vs RHE with a low scan rate of 5 mV/s to measure pressure fluctuations in the chamber as a function of applied potential. At the same time, the variation of CO partial pressure was monitored to detect any product formation under the applied potentials. As shown in Supplementary Figure 4, the partial pressure variation of CO increases with the applied potential following the LSV plot trend. The recorded CO formation onset potential of -0.1227 V for W_2C NFs is 12.7 mV higher than the thermodynamic value of CO_2 reduction to CO potential (-110 mV vs RHE) in eCO_2RR.

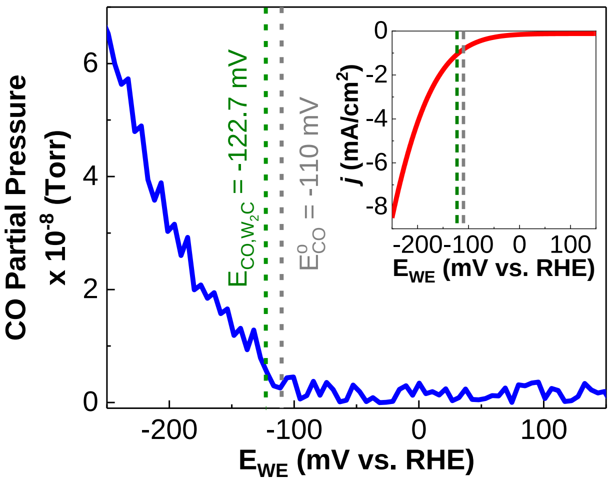

Supplementary Figure 4 | *In-situ* differential electrochemical mass spectrometry (DEMS) results of W_2C NFs. The CO partial pressure as a function of potential and corresponding LSV curve are shown.

5- Turnover Frequency Measurements (TOF) of TMCs

Turnover frequency (TOF) of TMC NFs were measured using roughness factor (RF) method to compare the actual catalytic activity of different catalysts in the electrochemical CO_2RR. To do so, the double layer capacitance (C_{dl}) of each catalyst were measured by performing cyclic voltammetry (CV) experiments in non-faradaic region at different scan rates. 0.1 M $HClO_4$ solution and Ag/AgCl electrode were used as an electrolyte and reference electrode, respectively. The CV results are shown in Supplementary Figure 5.

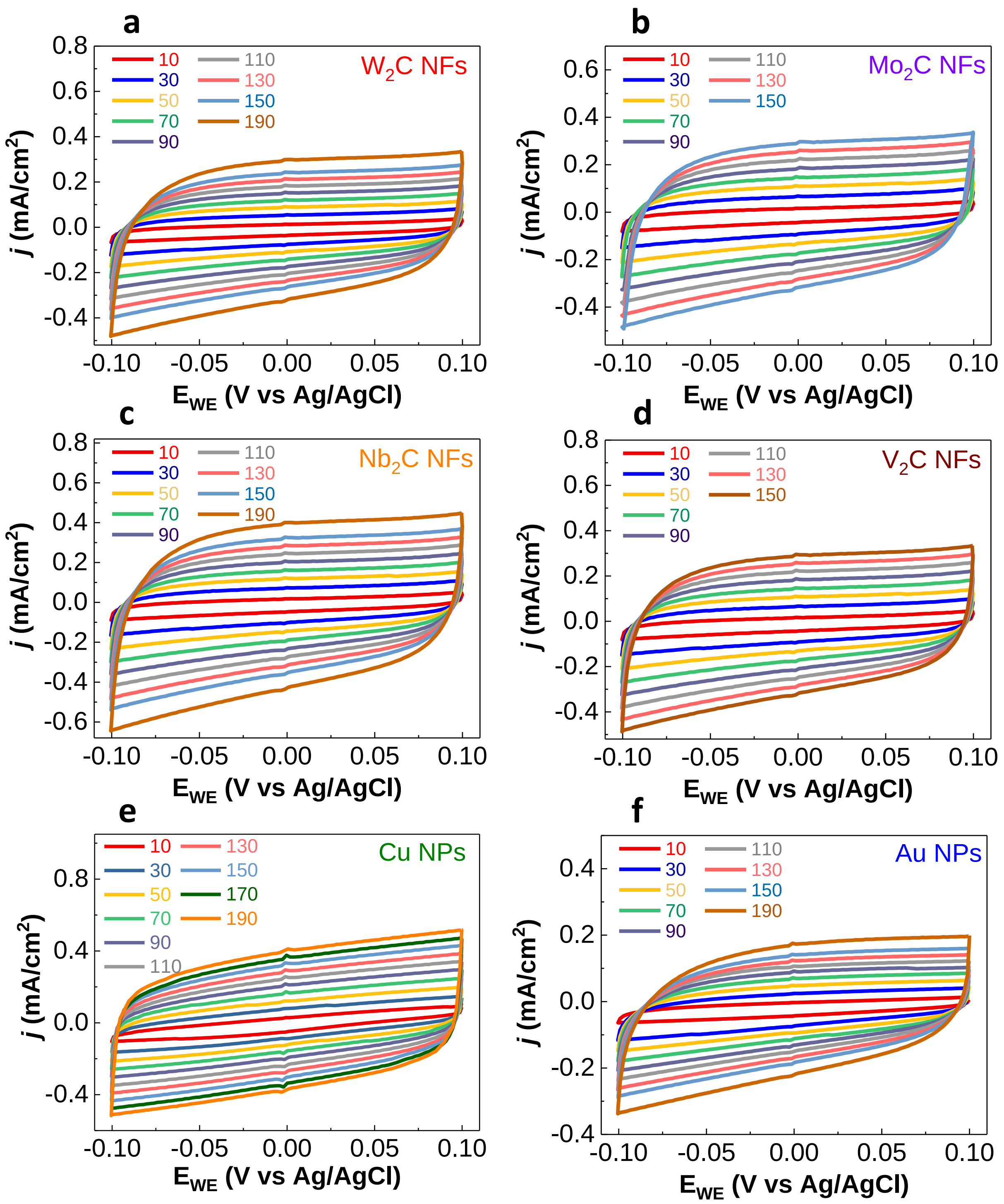

Supplementary Figure 5 | Cyclic voltammetry (CV) experiments for **(a)** W_2C NFs **(b)** Mo_2C NFs **(c)** Nb_2C NFs **(d)** V_2C NFs **(e)** Cu NPs and **(f)** Au NPs in non-faradaic region at different scan rates.

The C_{dl} value for each catalyst was then calculated from the slope of the “current density – scan rate” plot as shown in Supplementary Figure 6.

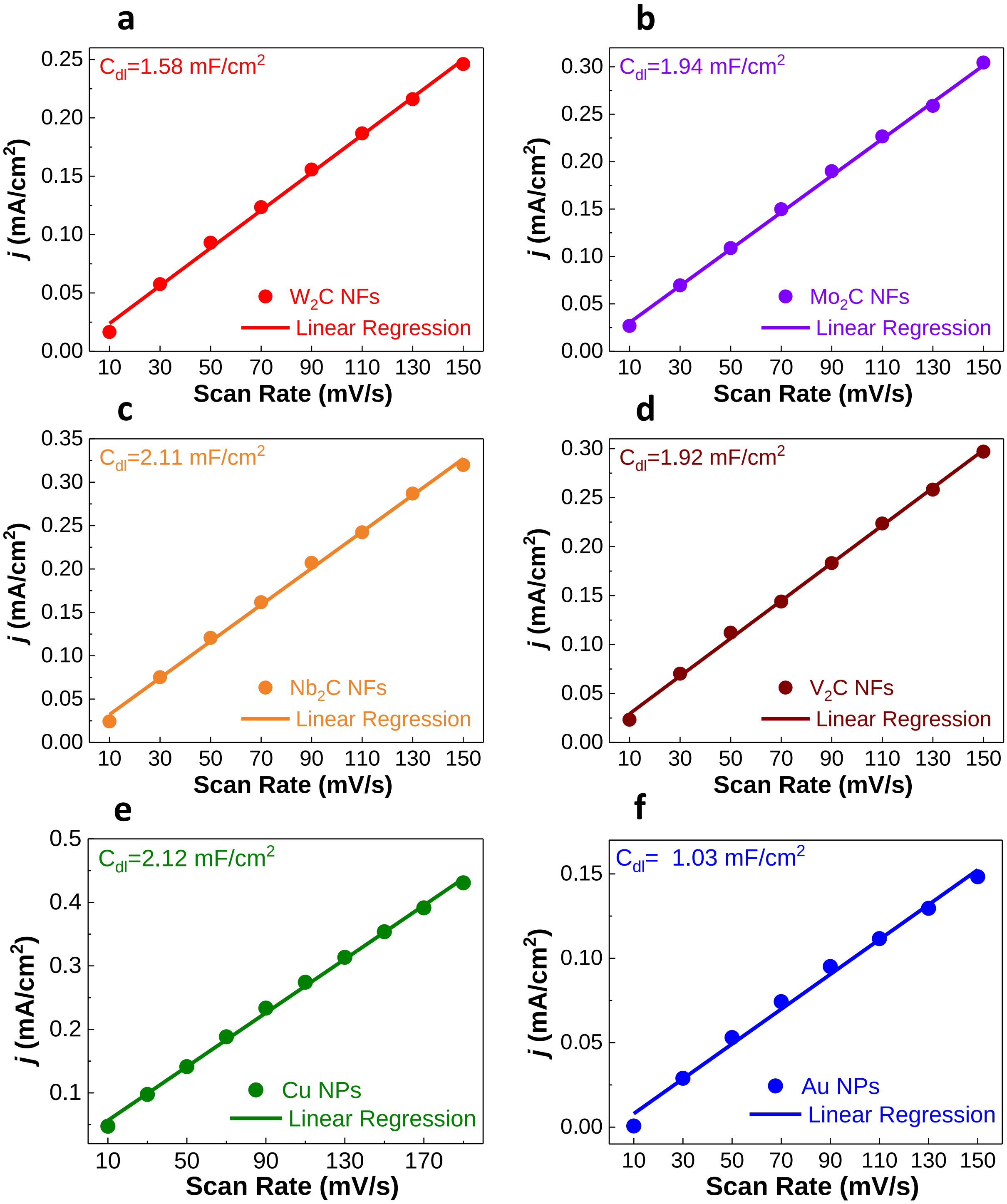

Supplementary Figure 6 | Double layer capacitance measurements for different catalysts.

The TOF of each catalyst then calculated from Equation 3 where the flat standard capacitor is extracted from literature.

$$Product\ \text{formation TOF (s}^{-1}) = \frac{j_{\text{product}}\ (\text{A/cm}^2)}{Density\ of\ active\ sites \times 1.602 \times 10^{-19}\ (C/e^{-}) \times n\ (e^{-})} \quad \text{(Equation 3)}$$

Where density of active sites was calculated by Equation 4:

$$Density\ of\ active\ sites\ (\mathrm{sites/cm^2}) = \frac{Density\ of\ metal\ atoms\ for\ standard\ samples\ (\frac{site}{cm^2})}{RF} \quad \text{(Equation 4)}$$

And RF values for different catalysts were measured using Equation 5.

$$\mathrm{RF} = \frac{C_{dl}}{flat\ standard\ capacitor} \quad \text{(Equation 5)}$$

The summary of active sites and RF calculations for different catalysts are presented in Supplementary Table 3.

Supplementary Table 3 | Summary of active sites and RF calculations for studied catalysts.

Catalyst	W_2C	Mo_2C	Nb_2C	V_2C	Au	Cu
**Flat standard capacitor (μF/cm²)**[19]	60	60	60	60	20	20
**No. of metal atoms for flat standard capacitor (x 10^{-15})**	1.22	1.27	1.24	1.29	3.04	3.18
**Double layer capacitor (mF/cm²)**	1.58	1.94	2.11	1.92	2.12	1.03
**Roughness factor (RF)**	26.33	32.33	35.33	32	106	53
**No. of metal atoms per cm² (x 10^{16})**	3.2	3.95	4.38	4.12	33.8	16.1

Supplementary Figure 7 shows CH_4 production and total CO_2RR TOF measurements for the studied catalysts in the three-electrode electrochemical cell.

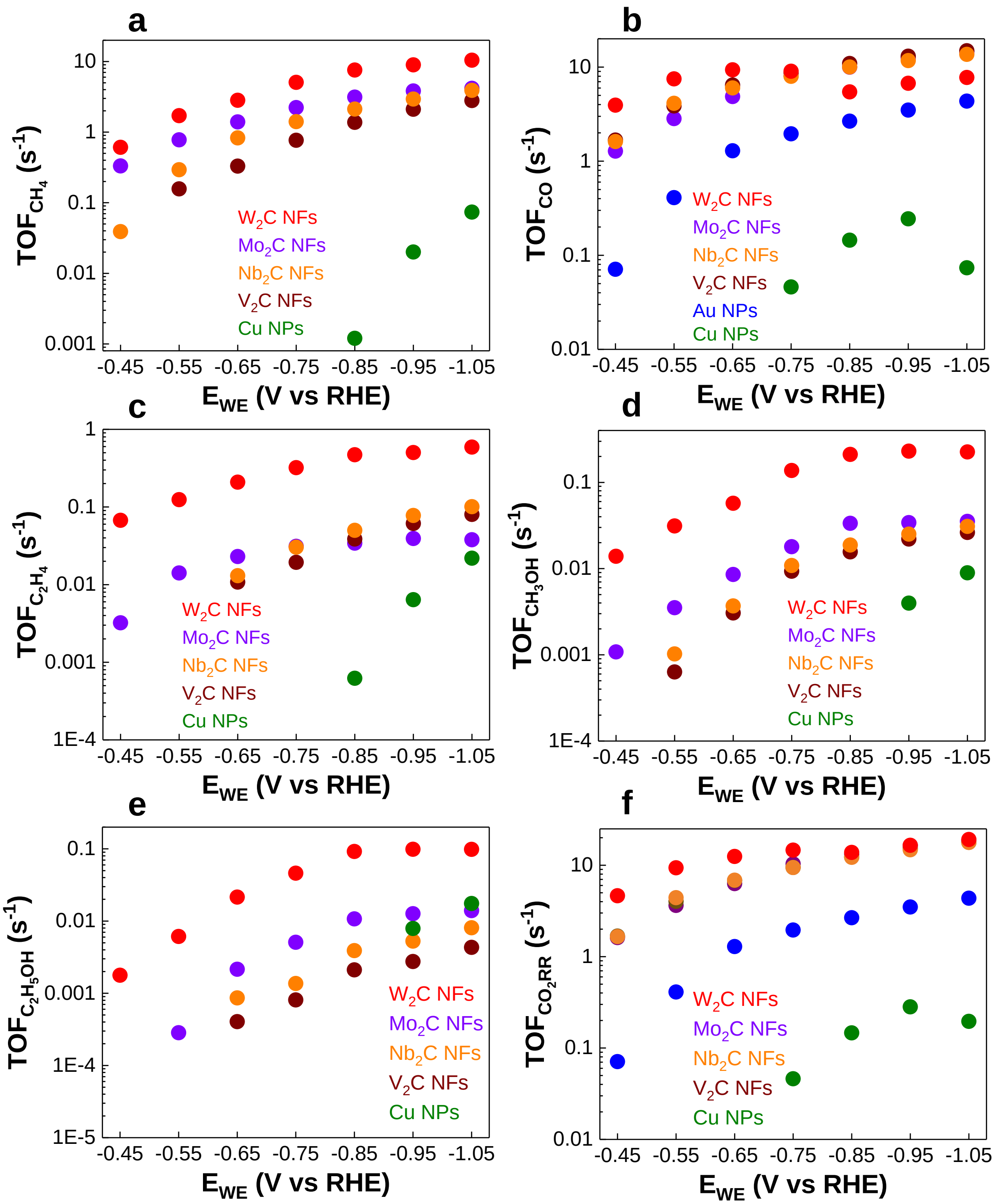

Supplementary Figure 7 | Turnover frequency (TOF) measurements of studied catalysts in three-electrode cell as a function of potential. (a) CH_4 formation TOF **(b)** CO formation TOF **(c)** C_2H_4 formation TOF **(d)** CH_3OH formation TOF **(e)** C_2H_5OH Formation TOF and **(f)** total CO_2RR TOF values of W_2C NFs, Mo_2C NFs, Nb_2C NFs, V_2C NFs, Au NPs and Cu NPs at different potentials.

6- Tafel Analyses of TMCs

The Tafel analysis for CH_4, CO, and C_2H_4 products were employed to study the kinetic behavior for formation of each product using W_2C, Mo_2C, Nb_2C, V_2C NFs, and Cu NPs. Supplementary

Figures 8a-c show the Tafel plots for CH_4, CO, and C_2H_4 products, respectively. Our Tafel plot analyses show that TMCs possess steeper Tafel slopes, and therefore a weaker potential dependence compared with Cu NPs for the formed products (i.e., CO, CH_4 and C_2H_4). This can be correlated to protonation of *CO intermediate as the rate-limiting step that is different than that of Cu catalyst where the C-O bond scission prior to *CO protonation is the rate determining step. Details are discussed in the theoretical study section (Supplementary section 13).

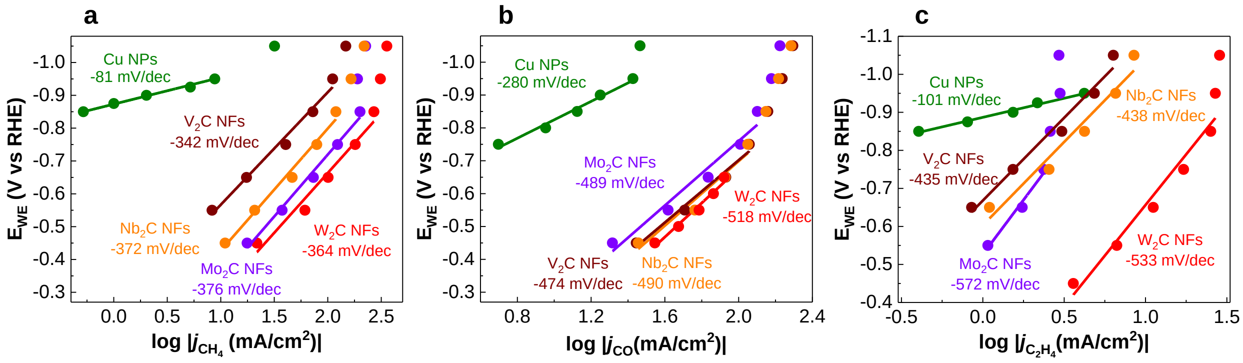

Supplementary Figure 8 | Tafel analyses of TMC NFs and Cu NPs.

7- X-ray Photoelectron Spectroscopy (XPS) of TMCs

X-ray photoelectron spectroscopy (XPS) spectra of synthesized transition metal carbides were obtained using a Thermo-Scientific ESCALAB 250Xi instrument equipped with an electron flood and scanning ion gun where the pressure of the analysis chamber was less than 2 x 10^{-9} Torr. The XPS instrument was calibrated prior to the experiments with binding energies all referenced to the Au $4f_{7/2}$ at 83.96 eV and Ag $3d_{5/2}$ at 368.27 eV. All samples were irradiated by a monochromatic Al-Kα X-ray source. All scans were acquired with a pass energy of 20 eV, 0.05 eV step size and dwell time of 200 ms using a charge neutralization system (an ion flood gun). The data processing was performed using the Avantage software such that W 4f, Mo 3d, Nb 3d and V 2*p* spectra were calibrated against the binding energy (BE) for adventitious carbon peak detected as a sharp intensity peak in the C 1s region (284.8 eV). For the curve-fitting, a Shirley background was used to consider the inelastic scattering. Supplementary Figures 9a-d indicates the XPS spectra of W_2C, Mo_2C, Nb_2C and V_2C NFs. The XPS spectrum of W_2C NFs depicts three

pronounced peaks centered at 31.7, 33.8 and 37.4 which are attributed to $4f_{7/2}$, $4f_{5/2}$ and $5p_{3/2}$ [20–22]. The XPS spectra of Mo_2C NFs also show the spin orbit split 3*d* peaks of $3d_{5/2}$ at binding energy of 228.3 eV and $3d_{3/2}$ at binding energy of 231.1 eV [23]. Similar to Mo_2C NFs, two peaks correspond to $3d_{5/2}$ and $3d_{3/2}$ at binding energies of 203.5 and 205.4 eV were observed in the XPS spectrum of Nb_2C NFs [24]. The XPS spectrum of V_2C NFs also indicates two peaks, corresponding to $2p_{3/2}$ and $2p_{1/2}$ at binding energies of 524.1 and 516.8 eV, respectively [25].

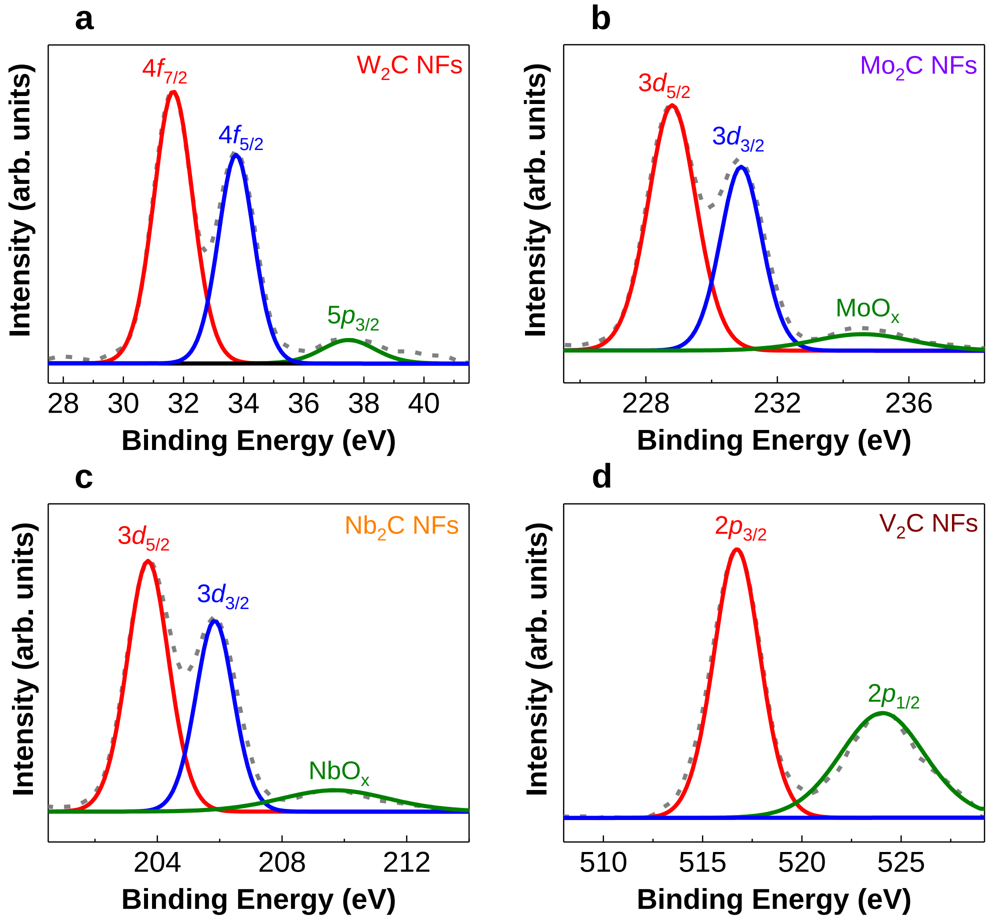

Supplementary Figure 9 | X-ray photoelectron spectra of **(a)** W_2C NFs **(b)** Mo_2C NFs **(c)** Nb_2C NFs and **(d)** V_2C NFs.

8- X-ray Diffraction (XRD) patterns of TMCs

The phase purity and crystallinity of synthesized samples were characterized by Bruker D2 PHASER diffractometer in Bragg–Brentano geometry employing a Ni filtered Cu *K*α radiation (1.5405 Å). The X-ray diffraction (XRD) patterns were obtained using a LynxEye linear position sensitive detector and a step width of 0.2 °2θ with a counting time of 1 s/step. All other parameters

were chosen to enhance the signal to noise in diffractograms. The XRD patterns of TMC NFs, Au NPs, and Cu NPs are shown in Supplementary Figures 10a-f.

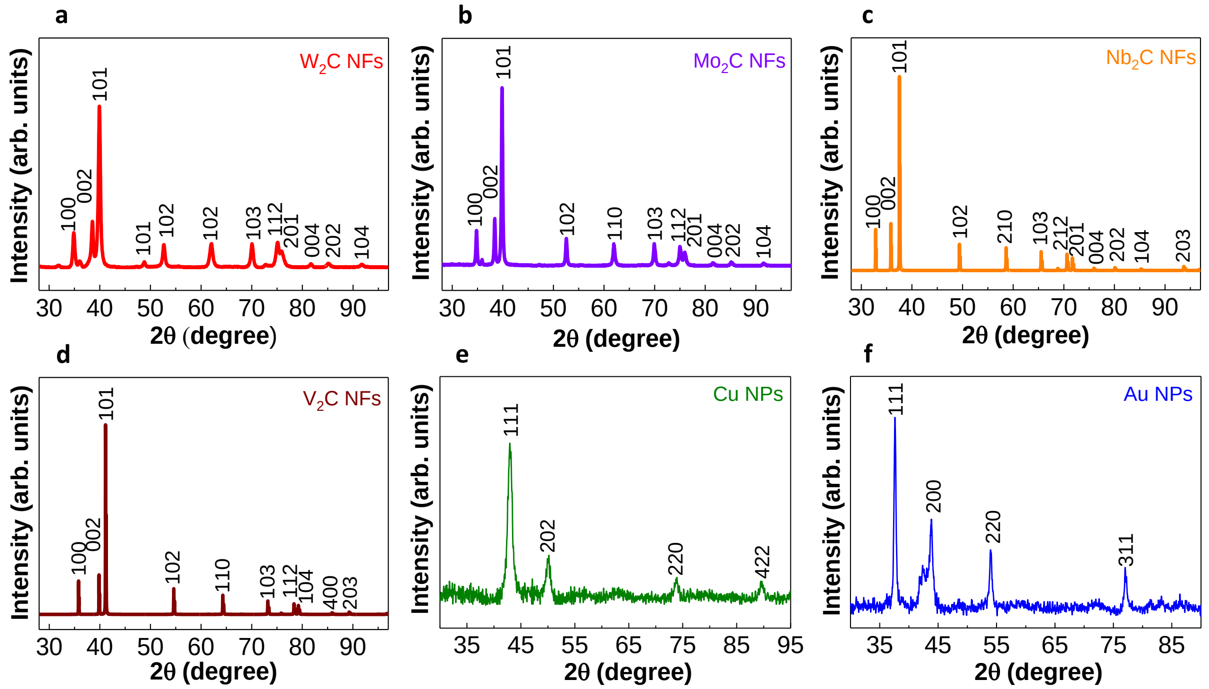

Supplementary Figure 10 | X-ray diffraction (XRD) patterns of **(a)** W_2C NFs, **(b)** Mo_2C NFs **(c)** Nb_2C NFs, **(d)** V_2C NFs, **(e)** Cu NPs and **(f)** Au NPs

The size of the coherently diffracting domains for synthesized TMC NFs, Au and Cu NPs were obtained using GSAS-2 software[26]. The Rietveld method was employed to refine and minimize the difference between the measured diffraction pattern and the standard phase patterns of each sample.[26] Then, the Bragg peaks of spectra were considered to calculated the crystallite sizes of synthesized catalysts using Scherrer equation[27,28].

$$P_{(nm)} = \frac{18000\, K\lambda}{\pi(LX - X_{inst})} \qquad \text{(Equation 6)}$$

Where λ is the wavelength of the X-rays (X-ray tube: Cu, λ = 0.154 nm), K is a numerical factor frequently referred to as the crystallite-shape factor. X_{inst} is the instrument broadening, and LX is the refined Lorentzian coefficient for size-type broadening[26]. Summary of the average crystallite sizes of catalysts are shown in Supplementary Table 4.

Supplementary Table 4 | Crystallite sizes of TMC NFs, Au and Cu NPs.

Sample	***Ave. crystallite Size (nm)***
W_2C NFs	23.7
Mo_2C NFs	21.4
Nb_2C NFs	29.5
V_2C NFs	27.2
Au NPs	31.6
Cu NPs	5.7

9- Scanning Transmission Electron Microscopy (STEM) of TMCs

The scanning transmission electron microscopy (STEM) imaging was conducted using a spherical aberration corrected JEOL JEM-ARM 200CF with a cold field emission gun operating at 200 kV. High-angle annular dark-field (HAADF) detector with 22 mrad inner-detector angle and bright field (BF) detector were utilized to obtain the atomic resolution images. Supplementary Figure 11 shows the atomic scale characterization results of synthesized W_2C NFs. Supplementary Figure 11a shows overall morphonology of the nanoflakes, and Supplementary Figures 11b and 11c show HAADF and BF images of W_2C in <101> zone axis. The corresponding fast Fourier transform (FFT) in Supplementary Figure 11d indicates diffraction spots from <101> zone axis. Supplementary Figure 11e show the atomic model in same zone axis. A zoom in of BF image in Supplementary Figure 11f indicates the C atomic columns and distance between two C atoms is 2.55 Å.

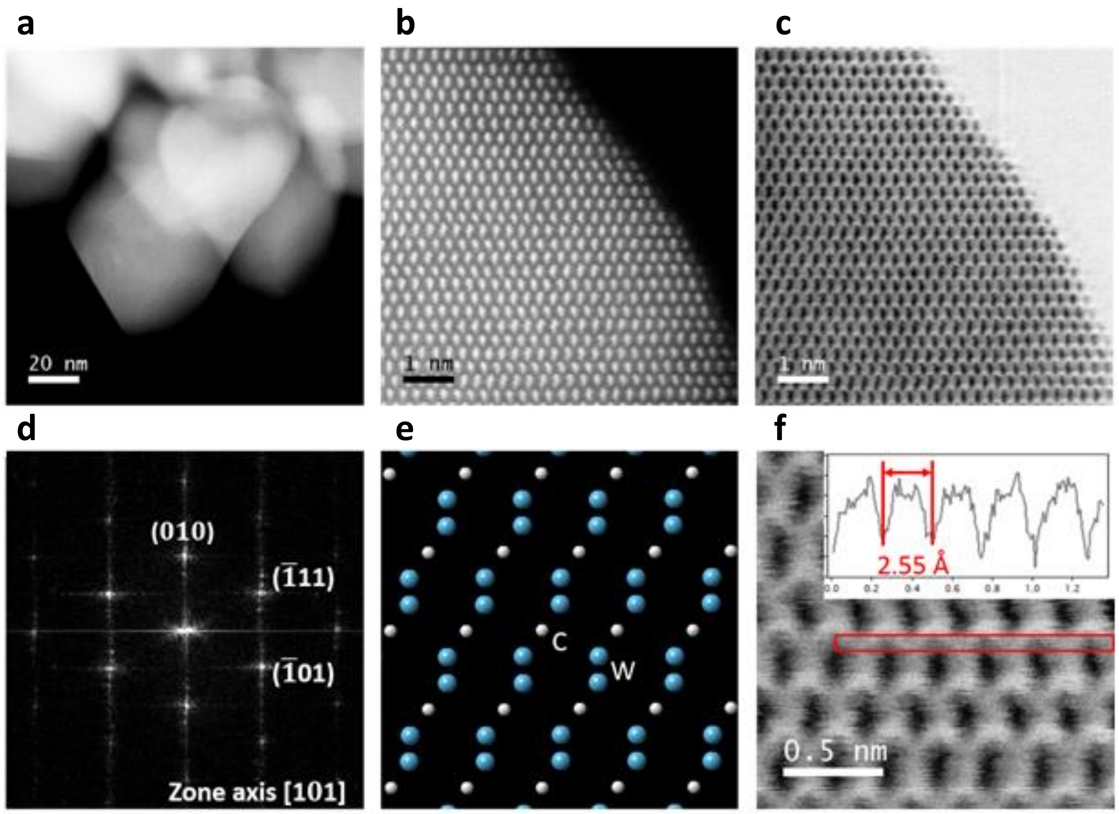

Supplementary Figure 11 | (a) HAADF image of W_2C NFs. **(b)(c)** HAADF and BF images of W_2C in <101> zone axis. **(d)** FFT corresponding to b and c showing the diffraction spots from <101> zone axis. **(e)** Atomic model of W_2C in <101> zone axis. W is shown as blue and C as white spheres. **(f)** Zoom in of S11c showing the C atomic columns in red box. The inset is intensity profile across red box region showing distance between two C atoms is 2.55 Å.

Supplementary Figure 12 depicts STEM images of synthesized Mo_2C NFs. Morphology of NFs is shown in Supplementary Figures 12a and 12b, as its layered structure is obvious from BF image, Supplementary Figure 12b.Tthe corresponding fast Fourier transform (FFT) in Supplementary Figure 12c indicates highlighted diffraction spots from <010> zone axis corresponding to (002), (101) and (100) planes. Supplementary Figures 12d and 12e show high resolution HAADF with measured d-spacings of 2.31 Å for (101) plane and 2.33 Å for (002) plane with angle 60° between them and BF images of Mo_2C in <010> zone axis, respectively. Supplementary Figure 12f depicts visual atomistic-level of detailed structure in the same zone axis.

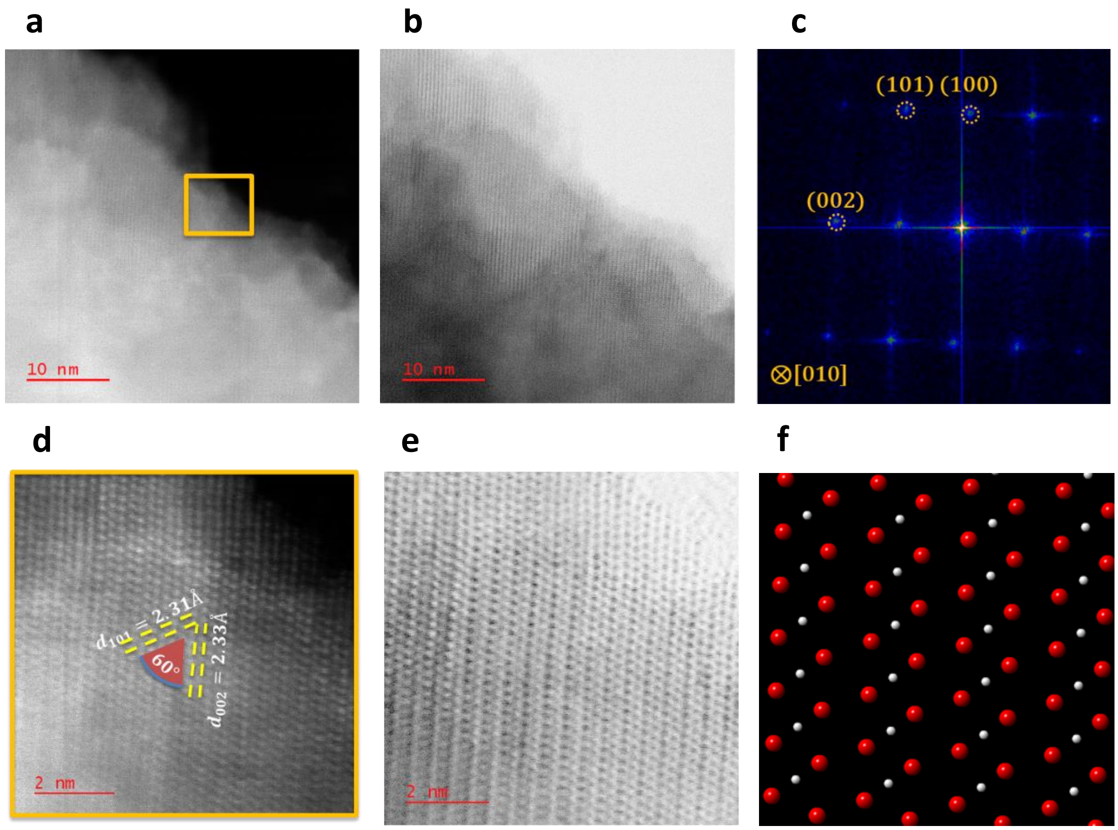

Supplementary Figure 12 | (a) Low-angle annular dark field (LAADF) STEM and, **(b)** corresponding BF images of Mo_2C NFs. **(c)** FFT corresponding to b illustrating the diffraction spots from the <010> zone axis. **(d)(e)** HAADF and BF images of Mo_2C showing the atomic structure in the <010> zone axis. **(f)** Atomic model of Mo_2C in <010> zone axis. Mo is shown as red and C as white spheres.

Supplementary Figure 13 depicts STEM results of synthesized Nb_2C NFs. Morphology of NFs is shown in Supplementary Figure 13a and 13b, and the corresponding fast Fourier transform (FFT) in Supplementary Figure 13c indicates highlighted diffraction spots from <210> zone axis corresponding to $(\bar{1}20)$ and (002) planes. Supplementary Figures 13d and 13e show high resolution HAADF with measured d-spacings of 2.49 Å for (002) plane and BF images of Nb_2C in <210> zone axis, respectively. Supplementary Figure 13f depicts visual atomistic-level of detailed structure in the same zone axis.

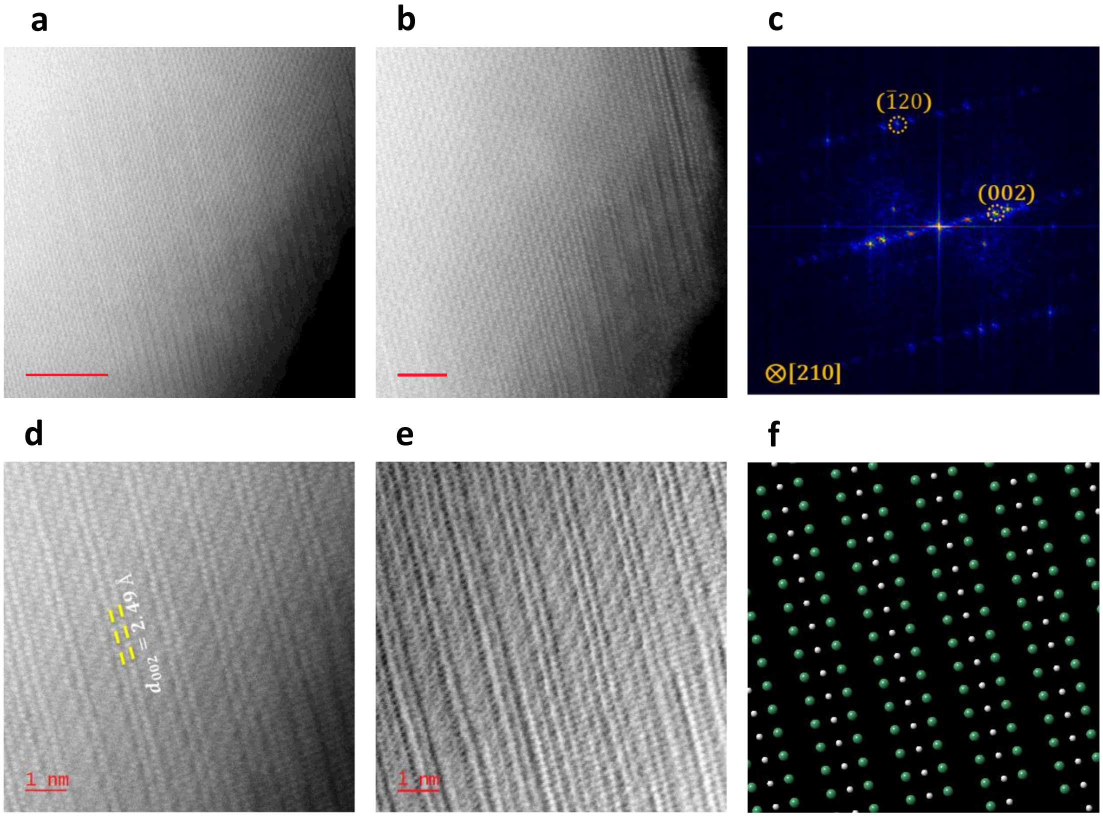

Supplementary Figure 13 | (a)(b) HAADF images of Nb_2C NFs. **(c)** FFT corresponding to b illustrating the diffraction spots from <210> zone axis. **(d)(e)** HAADF and BF images of Nb_2C in <210> zone axis. **(f)** Atomic model of Nb_2C in <210> zone axis. Nb is shown as green and C as white spheres.

Supplementary Figure 14 depicts STEM images of synthesized V_2C NFs. Morphology of NFs is shown in Supplementary Figures 14a and 14b, and the corresponding fast Fourier transform (FFT) in Supplementary Figure 14c indicates highlighted diffraction spots from <$1\bar{1}0$> zone axis corresponding to (002) and (110) planes. Supplementary Figures 14d and 14e show high resolution HAADF with measured d-spacings of 2.28 Å for (002) plane and BF images of V_2C in <$1\bar{1}0$> zone axis, respectively. Supplementary Figure 14f depicts visual atomistic-level of detailed structure in the same zone axis.

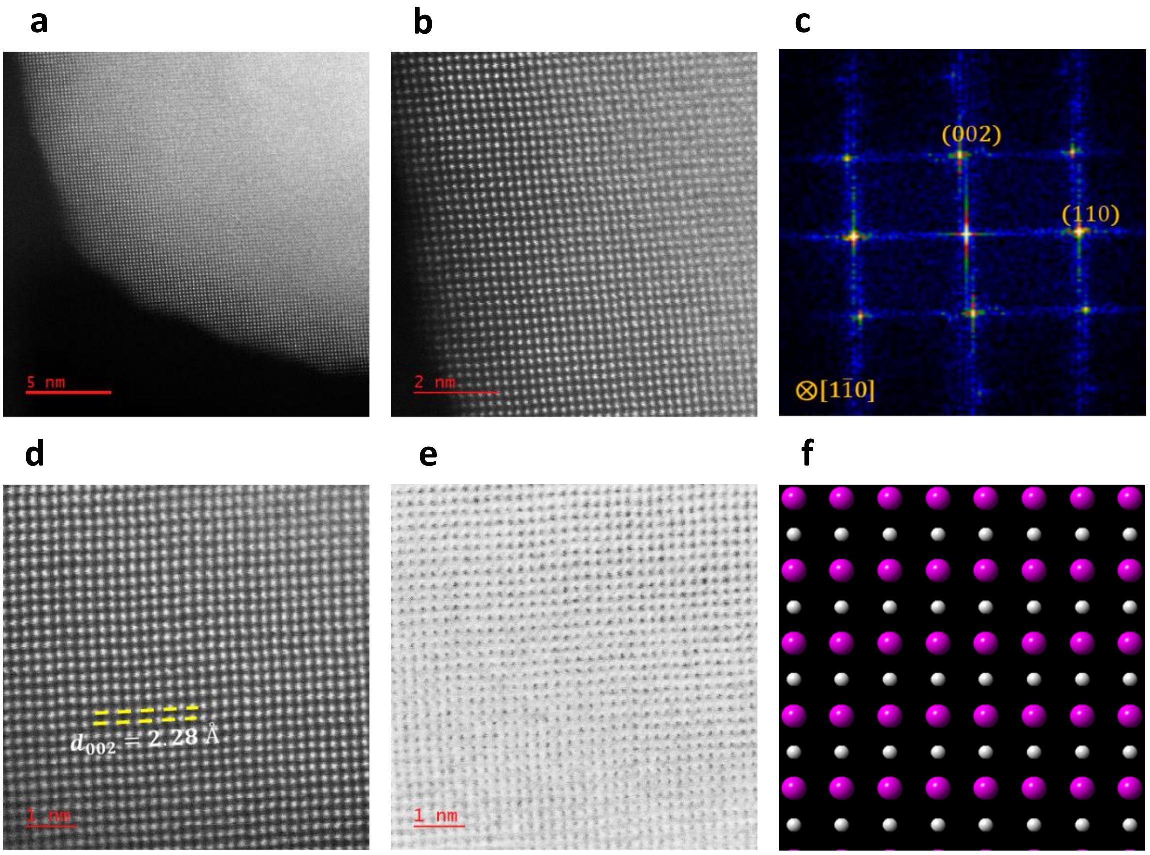

Supplementary Figure 14 | (a)(b) HAADF images of V_2C NFs. **(c)** FFT corresponding to b illustrating the diffraction spots from <1$\bar{1}$0> zone axis. **(d)(e)** HAADF and BF images of V_2C in <1$\bar{1}$0> zone axis. **(f)** Atomic model of V_2C in <1$\bar{1}$0> zone axis. V is shown as purple and C as white spheres.

Furthermore, we performed Scanning transmission electron microscopy-energy-dispersive X-ray spectroscopy (STEM-EDS) mapping to study the elemental distribution of catalysts. The results are shown in Supplementary Figures 15-18 reveal uniform elemental distribution and compositions confirming empirical formula of M_2C (M= W, Mo, Nb, V) for all synthesized TMCs.

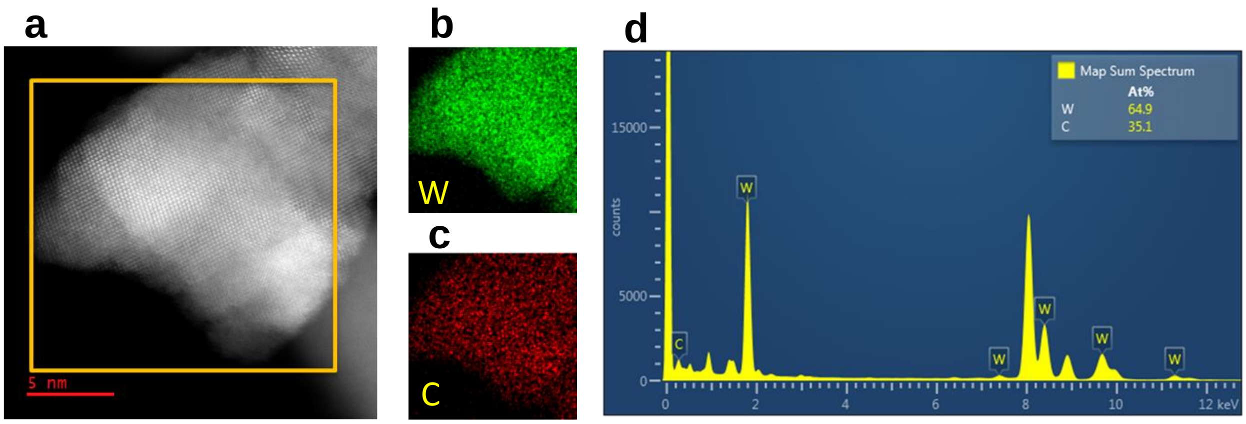

Supplementary Figure 15 | EDS element mappings of W_2C. **(a)** HAADF image of W_2C, **(b,c)** EDS mappings of W and C elements, **(c)** EDS spectrum and atomic percentage elemental analysis.

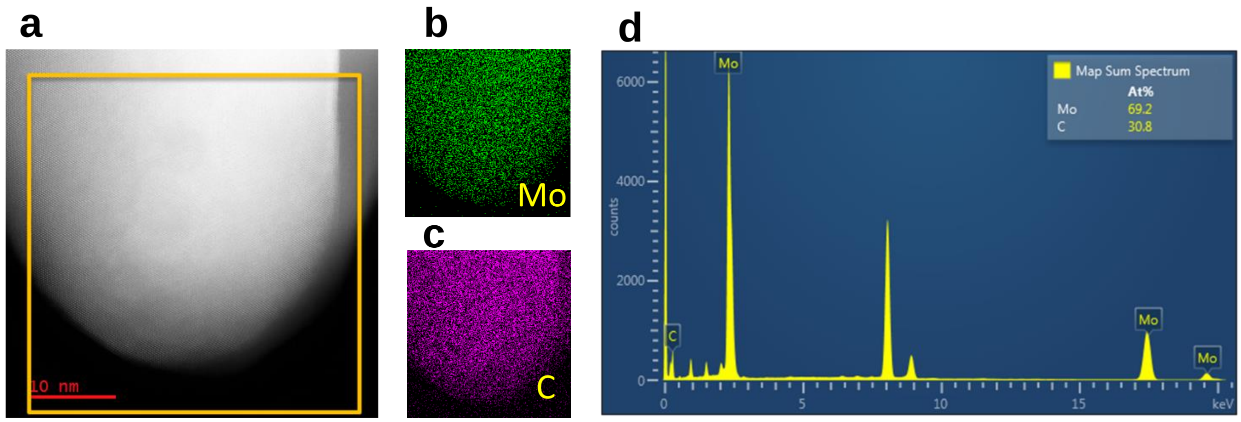

Supplementary Figure 16 | EDS element mappings of Mo_2C. **(a)** HAADF image of Mo_2C, **(b,c)** EDS mappings of Mo and C elements, **(c)** EDS spectrum and atomic percentage elemental analysis.

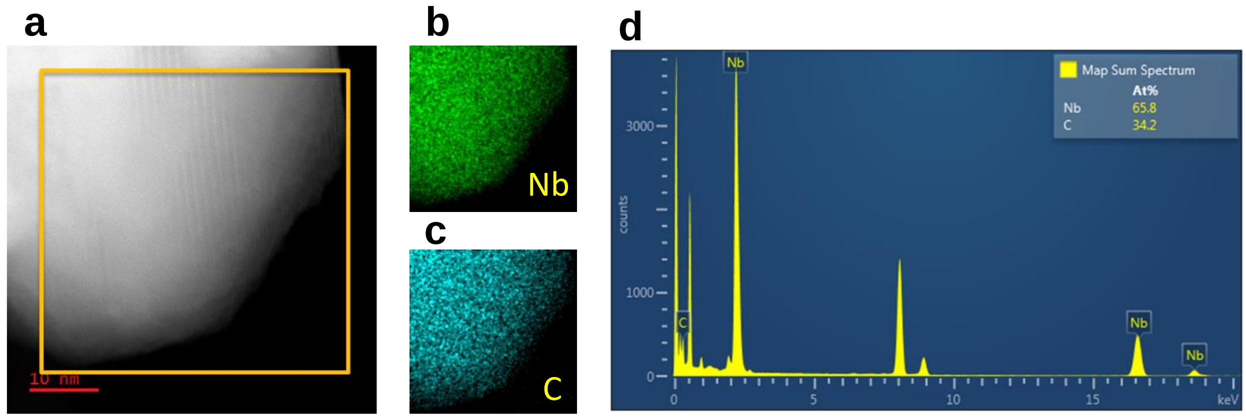

Supplementary Figure 17 | EDS element mappings of Nb_2C. **(a)** HAADF image of Nb_2C, **(b,c)** EDS mappings of Nb and C elements, **(c)** EDS spectrum and atomic percentage elemental analysis.

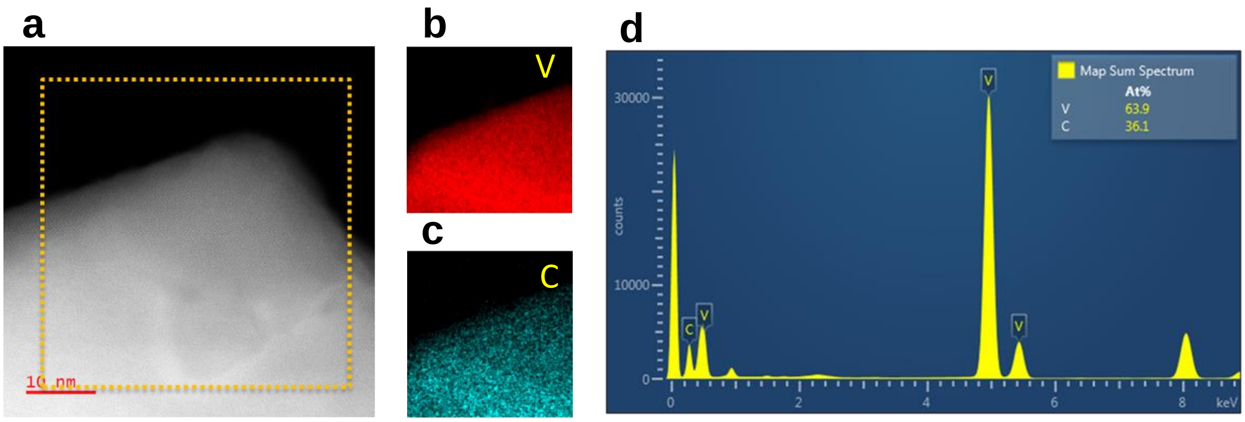

Supplementary Figure 18 | EDS element mappings of V_2C. **(a)** HAADF image of V_2C, **(b,c)** EDS mappings of V and C, (c) EDS spectrum and atomic percentage elemental analysis.

10- Dynamic Light Scattering (DLS) of TMCs

Dynamic light Scattering (DLS) experiments were carried out to measure flake sizes using the NiComp ZLS 380 system at 25ºC. The instrument includes a 35 mW semiconductor laser with 670 nm emissions and a thermoelectric temperature control for samples. Synthesized TMC NFs dispersed in isopropanol were used for DLS experiments. Typical error in DLS data is in the order of 5-8%. Supplementary Figure 19 represents flake size distributions of TMC NFs. The results show average sizes of 36, 35, 40 and 39 nm for W_2C, Mo_2C, Nb_2C and V_2C NFs, respectively.

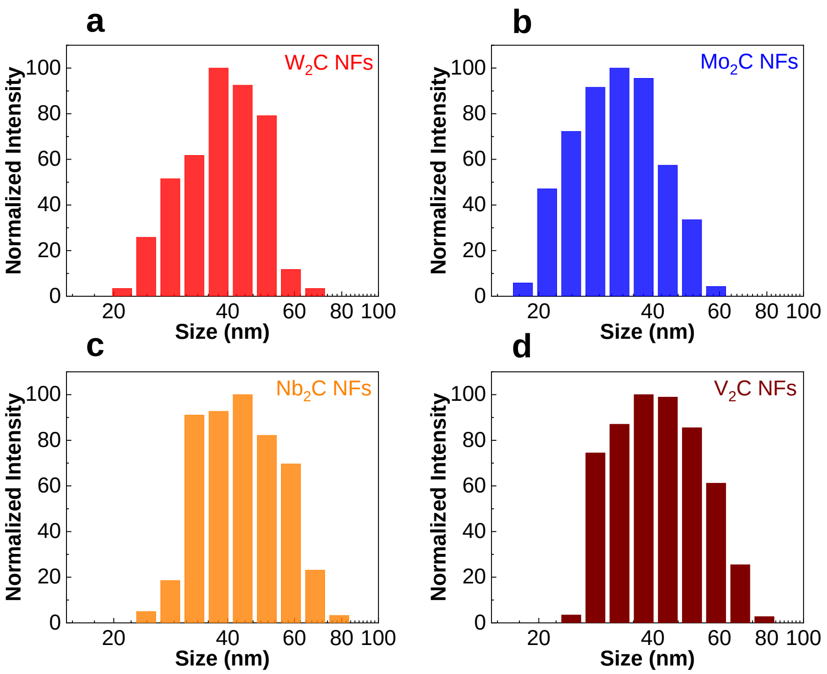

Supplementary Figure 19 | Size distribution of **(a)** W_2C NFs **(b)** Mo_2C NFs **(c)** Nb_2C NFs and **(d)** V_2C NFs.

11- Electrochemical Impedance Spectroscopy (EIS) of TMCs

In order to compare the charge transfer resistance (R_{ct}) of the developed TMC catalysts, we performed EIS experiments at the low overpotential of -310 mV vs RHE where the electrochemical results and product stream analysis showed that hydrogen evolution reaction (HER) and the electroreduction of CO_2 to CO are the only reactions happening at the surface of all the studied catalysts. The EIS experiments were performed under identical experimental conditions that includes factors such as mass loading (0.1 mg of each catalyst deposited on a glassy carbon substrate), surface area (1 cm^2), roughness factors (in a range of ~26-35, Supplementary section 5) inside a three-electrode electrochemical cell comprised of the catalysts as the working electrodes, platinum (Pt) gauze 52 mesh (Alfa Aesar) as the anode and Ag/AgCl (BASi) as the reference electrode. The Nyquist plots for different catalysts were recorded at a small amplitude of 10 mV and over a frequency range of 1 to 2 × 10^5 Hz. Supplementary Figure 20 show the fitted EIS spectra of each TMC catalyst at potentials of -310 mV vs RHE using Randles circuit model.

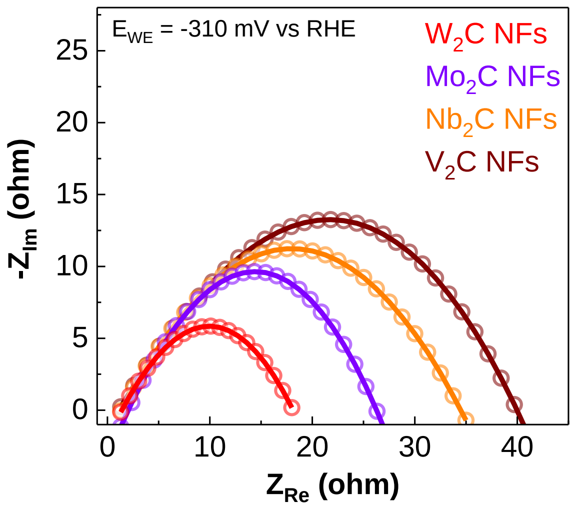

Supplementary Figure 20 | Electrochemical impedance spectroscopy (EIS) for studied catalysts at a potentials of -310 mV vs RHE in three-electrode electrochemical cell using KOH:CC (3M:2M).

A potential of -310 mV vs RHE is selected for our EIS experiment because it allows for precise measurement of the R_{ct} of each catalyst where the only happening reactions at TMC surfaces are the HER and the eCO_2RR to produce CO. In more details, the charge transfer resistance (R_{ct}) for each catalyst can be calculated using Equation 7.

$$R_{ct} = \frac{RT}{nFi_0} \qquad \text{(Equation 7)}$$

Where R is the universal gas constant, T is the temperature, F is the Faraday number, n is number of transferred electrons, and i_0 is exchange current density.

Since two competing reactions (HER and CO_2 reduction to CO) are happening in parallel on TMC catalyst surfaces, the observed R_{ct}, overall values in Nyquist plots are a combination of the R_{ct} values for HER ($R_{ct,H2}$) and CO_2 reduction to CO ($R_{ct,CO}$) as shown in Equation 8:

$$\frac{1}{R_{ct,overall}} = \frac{1}{R_{ct,H2}} + \frac{1}{R_{ct,CO}} \qquad \text{(Equation 8)}$$

By implementing R_{ct} for HER and CO_2 reduction to CO from Equation 7 and assumption of no other reactions involved in the process at -310 mV (that is a valid assumption based on our product stream analysis), the R_{ct}, overall becomes:

$$R_{ct,overall} = \frac{RT}{F\,(n_{H2}\, i_{0,H2} + n_{CO}\, i_{0,CO})} \qquad \text{(Equation 9)}$$

Considering n_{H2} and n_{CO} = 2 the overall R_{ct} is

$$R_{ct,overall} = \frac{RT}{2F\,(i_{0,H2} + i_{0,CO})} \qquad \text{(Equation 10)}$$

whereas $i_{0,H2}$ and $i_{0,CO}$ are dependent on the intrinsic properties of the studied TMC catalysts. Therefore, as shown in Equation 10, the R_{ct}, overall value observed in each catalyst at this potential (-310 mV vs. RHE) becomes only dependent on the summation of exchange current densities for HER ($i_{0,H2}$) and CO_2 reduction to CO ($i_{0,CO}$). This rationale, being valid at this low potential, enables us to conclude that the intrinsic electrocatalytic activity of W_2C NFs is higher than that of other studied TMC catalysts.

12- Work Function Measurements - UPS Method

To obtain more insight into the origin of the CO_2RR activity of TMC NFs, we compared the surface work function of TMC NFs that can be correlated to the surface activity of them. The surface work function measurements were carried out using a Thermo Scientific ESCALAB 250XI

photoelectron spectrometer with He I (21.2 eV) ultraviolet radiation and the pass energy of 8.95 eV. All measurements were referenced to a gold foil.

13- Theoretical Modeling and Calculations

13.1 General Methodology

To perform a comparative analysis of catalytic activity and reactivity we used density functional theory[29,30] Using the SIESTA package[31], we estimated the equilibrium configurations and density of states of model Au (111) and M_2C (101) (M = W, V, Mo, Nb) extended surfaces, modeled as slabs under periodic boundary conditions. The M_2C model structures were assumed to be in the 1T phase, belonging to the [P3¯m1] space group, as per the experimental characterization.All slab structures were carved out from bulk structures. To this end, the lattice parameters and atomic positions of bulk Au and M_2C were optimized using the PBE functional[32] with a double-zeta with polarization (DZP) localized basis set. The core electrons were represented via Troullier-Martins norm-conserving pseudopotentials[33]. Converged Monkhorst-Pack [34] k-point grids of size 9 x 9 x 9 were used to sample the Brillouin zone, together with a plane-wave cutoff of 300.0 Ry. The atomic positions were optimized using the conjugate-gradient algorithm until the maximum atomic force was lower than 0.04 eV/Å, and the cell parameters until the maximum stress component along each periodic direction was below 1 GPa. First, we optimized the positions and lattice parameters of the Au and M_2C bulk structures with increasing k-point grid densities until the change in energy was below 0.01 eV. The cell parameters were optimized until the maximum stress component along each periodic direction was below 1 GPa. The results are shown in Supplementary Table 5.

Supplementary Table 5 | Bulk phase results: converged k-point grid, calculated lattice parameters and lattice parameters in the literature.

		Calculated			Literature	
Material	Phase	K-point grid	a	c	a	c
V_2C	hP3/p_3m1/164	9x9x9	2.893	4.532	2.89,2.897	4.36
Nb_2C	hP3/164	7x7x7	3.349	5.349	3.12, 3.153	4.97, 5.004

Mo_2C	p_3m1/162	5x5x5	3.082	4.674	3.005	-
W_2C	hP3/p_3m1/164	7x7x7	3.054	4.650	3.001	4.728
Au	fcc	9x9x9	4.027	-	4.08[26]	-

Specifically, the resulting cell parameters of Au are in good agreement with the literature: a=4.027 Å, compared to a=4.08 Å [35]. The calculated lattice parameters of W_2C are in good agreement with the aforementioned XRD (Supplementary Figure 10) and TEM results (Supplementary Figure 11). The space group is [P3¯m1], and the calculated and experimental lattice parameters are, respectively, a=3.045 Å, c=4.669 Å and a=3.070 Å, c=4.678 Å; with α=β=90° and γ=120° in both cases.

All slab structures were generated from the relaxed bulk structures by creating supercells of size 1 x 1 x N (where N is 2 and 5 for Au and 5 and 7 for TMCs). The surface atomic positions were relaxed on each slab, with fixed bulk lattice parameters and the positions of the atoms in the center of the slab, in order to mimic the bulk conditions at the center of the slab. A vacuum of more than 12 A in the direction perpendicular to the surface was added in all calculations so as to avoid spurious interactions between periodic images. The k-point grid used for each slab in the x and y direction was the same as shown in Supplementary Table 5, while 1 k-point was enough to sample the Brillouin zone in the direction perpendicular to the slab.

We carried out a comparison of surface energies between different terminations that appear in the XRD profiles of the metal carbides using 5-layer slabs. The surfaces terminations included are the (101), (100), (002) and (102) planes, as well as the (111), which does not appear as a peak on the experimental XRD characterization of any of the materials, for comparison. The slab structures are shown on Supplementary Figure 21. Noticeably, all slabs except for the (111)-terminated one have a clearly layered structure. This leads to asymmetric, complementary C-rich and M-rich surfaces on either side of the slab, and hence an overall surface dipole as well as local dipoles on the top/bottom surface layers due to the M/C rich layering.

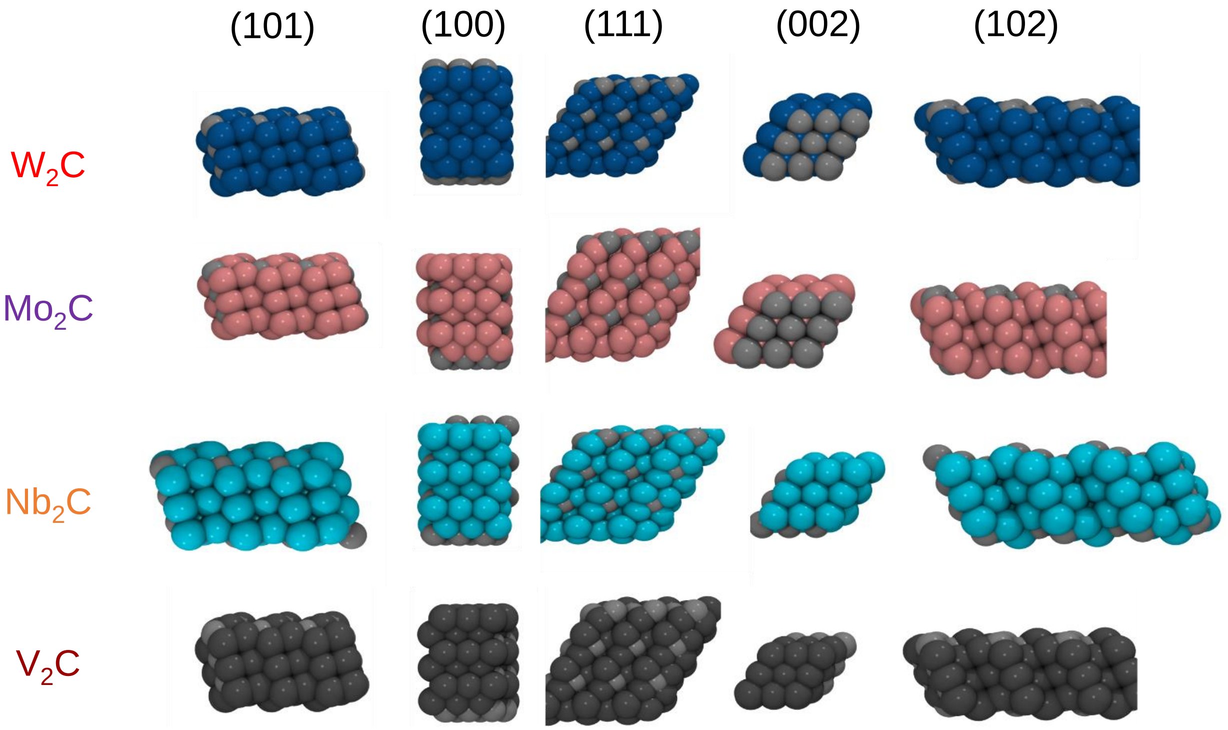

Supplementary Figure 21 | Top-view 2x2 supercells of the (101), (100), (002) and (102) 5-layer slabs of the metal carbides used to calculate the surface energies. Structures for M_2C (M =W, Mo, Nb, V) are the 1, 2 3 and 4th row, respectively.

The surface energies (Supplementary Table 6), E_{sub} were calculated as: E_{sur} = ½ (E_{slab}-E_{bulk} N) / A, where E_{slab} is the total energy of the N-layer slab, E_{bulk} is the total energy of the bulk unit cell, and A is the surface area of said termination. The variation in the surface energies between different slab terminations lies within 0.1 eV/AA^2. Deviations from the experimentally observed dominating surfaces are likely due to the level of theory used (a GGA exchange-correlation functional) because of computational efficiency.

Supplementary Table 6 | Surface energies (eV/A^2) for a 5-layer slab of each TMC.

	(101) 5L	(111)	(100)	(002)	(102)
V_2C	0.26	0.25	0.28	0.37	0.28
Nb_2C	0.14	0.14	0.14	0.17	0.14
Mo_2C	0.22	0.20	0.21		0.23
W_2C	0.27	0.23	0.24	0.28	0.29

Finally, density of states and charge distribution were evaluated using a more diffuse basis set by means of decreasing the orbital confinement cutoff radii to 0.001 Ry. The density states of the

slabs was calculated using the Effective Screening Method (ESM) developed by Otani et al. and implemented in SIESTA[36]. This method provides a common vacuum reference for the electrostatic potential in vacuum away from the slab for all calculated systems. Thus, the energy scale of the density of states and Fermi levels is aligned to a common reference and hence comparable between the different materials (Supplementary Figure 22).

We used vacuum-vacuum boundary conditions (bc1) for the electrostatic potential setting the reference zero values of the potential in the vacuum region of the simulation cell, far from the atoms in the slab.

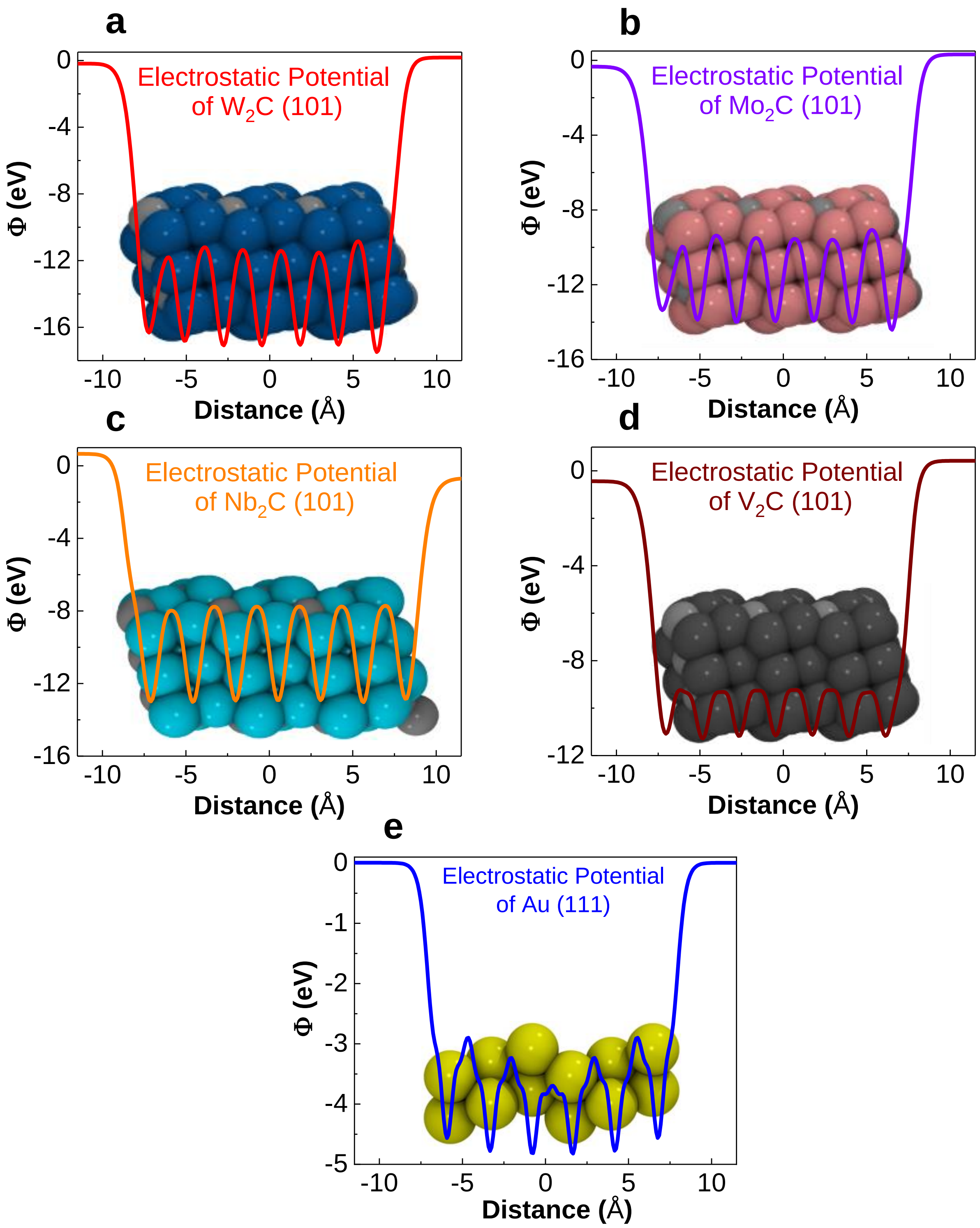

Supplementary Figure 22 | Electrostatic potentials (eV) along the direction perpendicular to the 7-layered (101) terminated **(a)** W_2C, **(b)** Mo_2C, **(c)** Nb_2C, **(d)** V_2C slabs, as well as **(e)** Au(111) slab, showing the common vacuum reference for the Fermi level away from the surface as an outcome from the ESM calculations.

Supplementary Figure 23 shows the Fermi level and 5*d* projected density of states (PDOS) corresponding to the outermost atoms of each slab. We normalized PDOS per atom (Au and W, respectively) The amount of d character states of a metal at the Fermi level indicates the number and the availability of d electrons that could be involved in charge transfer processes. The majority of the 5*d* PDOS of Au (111) surface atoms is ~1.5 eV below its Fermi level in these calculations. On the other hand, at the Fermi level, the W_2C (101) surface W atoms have close to 10 times more 5*d* states available.

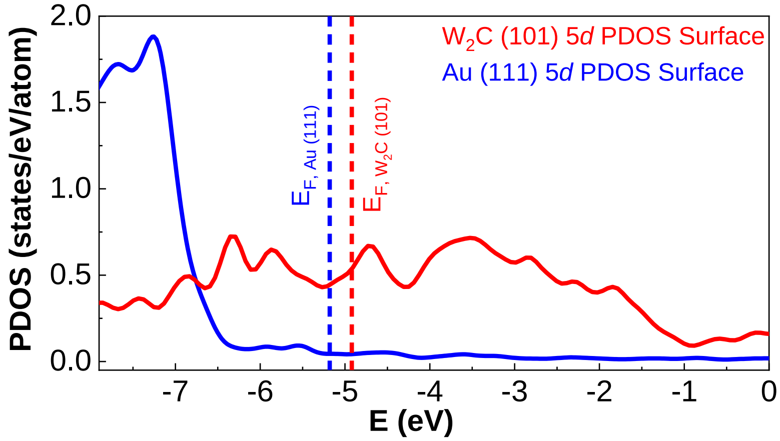

Supplementary Figure 23 | Comparison of the projected density of states corresponding to 5*d* states of surface atoms for Au (111) and W_2C (101), shown together with the Fermi level of each of the slabs.

For analysis of the charge distribution on the surfaces and adsorption free energies of various molecular species on the (101) surface of W_2C, we used the Vienna ab initio Simulation Package (VASP, version 5.4.4)[37,38]. The PAW[39] method and PBE[40] parametrization of the exchange-correlation functional are used. To account for the van der Waals (vdW) interactions, the dispersion corrected DFT-D3[41] scheme was employed. The kinetic energy cut-off for the plane-wave basis was set to 600 eV. The energy convergence threshold was set to 10^{-6} eV. We employed the asymmetric (W- and C-terminated on either side) slab geometry of the unit cells (Supplementary Figure 24) containing 120 atoms of W_2C (80 W-atoms and 40 C-atoms) to have a sufficiently large W-terminated surface area to accommodate surface molecular species avoiding image-image interactions. The same size unit cells (80 M-atoms and 40 C-atoms, where

M = V, Mo, Nb) were used for other studied TMCs. All the VASP calculations were performed for neutral non spin-polarized systems and dipolar electrostatic correction was used along the normal to the surface of the slab. The atomic relaxation and unit cell optimization were performed first for a pristine W_2C slab with the interslab distance of 50 Å and a fixed cell volume. A conjugate gradient algorithm was used for ionic optimization. Relaxation criterion was set to 10^{-4} eV for energies and 10^{-2} eV/Å for forces. To enhance the convergence of the electronic energy, we used Gaussian smearing with σ=0.1 (the difference between total energy and the electronic free energy was less than 1 meV/atom) and due to the large size of the systems, it was sufficient to sample the first Brillouin zone at the Γ-point only. Then various molecular species were optimized atop W-terminated (101) surfaces for fixed cell parameters. Once the atomic structures are optimized we used the tetrahedron method with Blöchl corrections and 1 x 3 x 3 Monkhorst-Pack[34] grid k-point sampling for the calculations of total electronic energy (smearing σ=0.1).

As shown in Supplementary Figure 24, Bader charge analysis indicates that top M-atoms in the (101) M_2C slabs are significantly reduced (two types of surface M-atoms0 as compared to M-atoms in the bulk regions. Specifically, Bader charges of (101) W_2C top W-atoms are +2 and +0.46 whereas for bulk W-atoms Bader charges are +0.7 to +0.85. This analysis also shows that (101) W_2C has the lowest Bader charges among other TMCs.

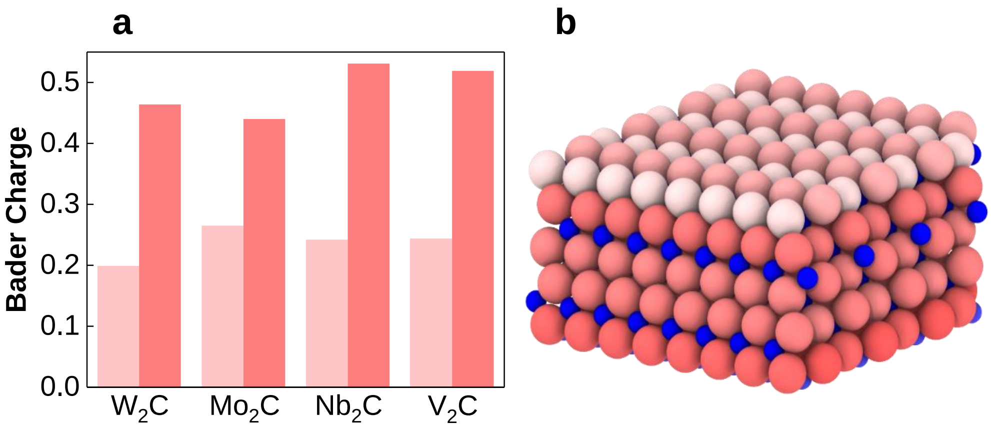

Supplementary Figure 24 | (a) Distribution of Bader charges in the (101) M_2C slabs. Light pink and pink colors correspond to two types of the surface metal atoms. **(b)** Distribution of Bader charges in (101) W_2C in the slab. Big and small balls correspond to of W- and C-atoms, and pink and blue colors correspond to positive and negative charges.

The thermal corrections to electronic energies (enthalpic temperature correction, entropy and zero-point vibrational energies) were calculated using vibrational frequencies calculated for slabs with relaxed adsorbed molecular configuration using only the Γ-point sampling. All atoms were free to move.

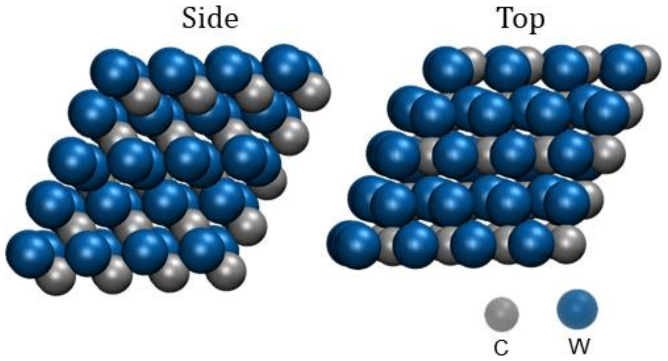

Supplementary Figure 25 | (101) W_2C slab geometry used in ab initio calculations.

For these phonon calculations, we used 10^{-6} eV energy convergence threshold to obtain reliable phonon frequencies (no negative eigenvalues.) To obtain the Free energy, G = H – TS, for the various equilibrium configurations, we used density functional perturbation theory (DFPT). For each of the vibrational modes, ν, a characteristic vibrational temperature can be expressed as $\Theta_{K,v} = h\nu/k_B$ (where K is the index of the vibrational mode). If we choose the first vibrational energy level as the zero of energy, then the partition function of this subset of simple harmonic oscillators is expressed as $q_{K,v} = \frac{1}{1-e^{-\Theta_{K,v}/T}}$, where the overall partition function, sampling the first Brillouin zone, is $q_v = \prod_K \frac{1}{1-e^{-\Theta_{K,v}/T}}$. The total vibrational entropy contribution is $S_v = R(\ln(q_v) + T\left(\frac{\partial \ln(q_v)}{\partial T}\right)_v = R\sum_K \left(\frac{\frac{\Theta_{K,v}}{T}}{e^{\frac{\Theta_{K,v}}{T}}-1} - \ln\left(1-e^{-\frac{\Theta_{K,v}}{T}}\right)\right)$. The total ZPE contribution is $ZPE = \sum_k \frac{1}{2} R\Theta_{K,v}$. The total enthalpy contribution is $H_v = R\sum_K \frac{\frac{\Theta_{K,v}}{T}}{e^{\frac{\Theta_{K,v}}{T}}-1}$. The overall vibrational contribution to the free energy, within the simple harmonic oscillator approximation, is $G_v = ZPE + H_v - T\,S_v$.

The identical computational protocol was applied to other studied TMCs: Nb_2C, Mo_2C, and V_2C.

13.2 Free Energy Calculations and Computational Hydrogen Electrode Model

The free energies of CO_2 reduction intermediates were approximated by total electronic energies corrected by zero-point vibrational energies, enthalpic temperature corrections and entropy: $G = E_{elec} + ZPE + H(T) - TS$, where E_{elec} includes the vdW corrections. Established methods[42] were used to calculate zero-point energies and vibrational entropies. The free energies of all considered intermediates along with the corrections are listed in Supplementary Tables 7 and 8. The fundamental assumption in these calculations is that we neglect the solvation energies of all species, and effects of dielectric and ionic screening from the bulk solution (water with KOH:CC (3M:2M) saturated with CO_2 gas). We also assume the dilute limit and consider one molecular species per surface slab unit cell.

Supplementary Table 7 | Electronic energies (E_{elect}), obtained at PBE/DFT-D3 level, zero point vibrational energy corrections (ZPE), Enthalpic temperature corrections ($\int C_P dT$), and Entropy contribution (S) to the Gibbs free energies at 298.15 K (G=E_{elec}+$\int C_P dT$+ZPE-TS) for different states along CO_2 reduction on W_2C (101).

Species	E_{elec}, eV	ZPE, eV	$\int C_P dT$, eV	S, eV/K	G, eV
**W_2C (clean 101 surface)**	-1385.433672	6.748433	4.795152	0.032923	-1383.701141
**CO_2***	-1410.362526	7.119794	4.859436	0.032955	-1408.203886
**H_2O* (dissociated state)**	-1402.107001	7.38717	4.840086	0.032879	-1399.677687
**CO***	-1402.398986	7.010625	4.837501	0.033088	-1400.411084
**O***	-1393.830394	6.879771	4.827774	0.032787	-1391.893375
**OH***	-1397.467167	7.18209	4.836313	0.032875	-1395.245514
**H***	-1388.910472	6.971895	4.810434	0.032808	-1386.904927
**HO-CO***	-1413.377085	7.424992	4.872422	0.033031	-1410.922909
**OCHO***	-1414.096905	7.408883	4.873266	0.033088	-1411.67498
**HCO***	-1406.343966	7.26831	4.852955	0.03297	-1404.047761
**H_2CO***	-1410.037936	7.553619	4.855623	0.033018	-1407.468058
**H_3CO* (CH_3*+O*)**	-1414.7222	7.786552	4.89005	0.033248	-1411.953502
**HCOOH* (formic acid)**	-1417.148951	7.719606	4.88698	0.033214	-1414.440137
**H_2CO_2***	-1417.432245	7.678349	4.889048	0.033293	-1414.786162
**CO*+O* (dissociated CO_2)**	-1411.293803	7.086832	4.868894	0.033008	-1409.174461
**CH_3***	-1405.972625	7.72836	4.856605	0.033033	-1403.231494

* indicates the adsorbed state. When species is unstable, the final state of optimization is shown in parentheses

To account for the effect of the electrified electrode-electrolyte interface and to put all the free energies in the experimentally accessible electrochemical scale we use the computational

hydrogen electrode (CHE) model[43–45] which relates free energies obtained at zero potential with respect to the reversible hydrogen electrode (RHE) to those at non-zero RHE potential: $\Delta G_n(\varphi) = \Delta G(\varphi = 0) + ne\varphi$, where φ is the electrode potential vs. RHE.

Supplementary Table 8 | Chemical potential (μ) of gas phase molecules reported[46] and used in the current study

Species	Eelec (eV)[a]	μ (eV)[a]	Eelec (eV)[b]	μ (eV)[b]
**CH_4 (gas)**	-24.03	-23.34	-24.05	-23.33
**CO_2 (gas)**	-22.99	-23.26	-22.95	-23.28
**H_2 (gas)**	-6.76	-6.76	-6.77	-6.76
**H_2O (gas)**	-14.23	-14.18	-14.23	-14.17
**CO (gas)**	-14.80	-15.25	-14.79	-15.27
**HCOOH (gas)**	-29.91	-29.96	-29.85	-29.95

[a] – ref. 46; [b] – our calculations

According to the convention in VASP calculation, total electronic energies are referenced to the energies of isolated non-spin-polarized atoms which are used in the generation of the pseudopotentials. As long as the total number of atoms is preserved in the reaction of interest, the differences in free energies of reactions that involve these chemical potentials are valid.

As one can see the difference in E_{elec} and μ_{el} that we calculated and those from ref. 46 is of order 0.01-0.02 eV, which is expected since the same code and the same level of theory was used.

An important point is the adjustment of μ_{el} of gaseous species to the experimental condition that are normally performed by adding pressure corrections calculated from experimental Faradaic efficiencies converted to molar yields[45]. To take those into account, we proceed as follows. Firstly, we define the reference standard thermodynamic state for all the species. For the gaseous species, specifically reagents and products that are poorly soluble in water, we define the standard state of CO_2, CO and CH_4 gases as an ideal gas at 1 atm at T = 298.15 K. For species in the liquid phase (water and formic acid), the standard state implies their activity taken as unity. For pure water at T = 298.15 K it corresponds to a concentration of 55.34 M. Therefore, to calculate the chemical potential of liquid water, a number of thermodynamic corrections needs

to be applied to the chemical potential of vapor water ideal gas. The free energy change of 1 mol of an ideal gas from 1 atm (24.46 L/mol) to 1 M (1 mol/L) gas state can be calculated as ΔG_1 = kTln(24.46) = 0.082 eV. The free energy of self-solvation of water can be calculated using the saturated liquid and vapor densities (55.34 mol/L and 1.281×10^{-3} mol/L, respectively, see J. Phys. Chem. **112**, 9709 (2008)). This gives $\Delta G_2 = kT\ln(1.281 \times 10^{-3}/55.34) = -0.274$ eV. Finally, the free energy change from 1 M to 55.34 M is given by ΔG_3 = kTln(55.34) = 0.103 eV. The sum of these corrections gives the overall correction ΔG = -0.089 eV. This value is very close to the adopted one in ref. 45 obtained from an assumed fugacity of the vapor pressure in equilibrium with liquid water 3534 Pa, which gives the correction of -0.084 eV. To be consistent with previous studies, here we use the latter value of the correction of the water vapor chemical potential. The standard state for the aqueous solution of formic acid could be its 1 M ideal solution. Due to the high solubility formic acid, its vapor pressure at standard concentration is very low which could be well below detection limit. Given the experimental conditions where no appreciable amount of formic acid is detected in the gas phase, the correction to the gas phase chemical potential of formic acid cannot be determined. In this case, we use its gas phase state as a reference state.

Secondly, to account for the experimental conditions, we define experimental fugacities of all relevant species produced with W_2C (101) catalyst at the working electrode potential that corresponds to the maximal production of methane. Based on the experimental data (see Supplementary Table 1), this potential is -850 mV vs. RHE. We then follow the procedure described in the original Peterson's paper (ref. 45) to convert our experimental Faradaic efficiencies of various products to their molar fractions and assumed fugacities that we use to compute the corrections to the ideal gas chemical potentials of these species. In Table S9 we now show the data and the results of calculations.

Supplementary Table 9 | Chemical potential (μ) of gas phase molecules and experimental corrections

Species	μ, eV[a]	Faradaic yield, %	Molar yield, %	Fugacity, Pa	Fugacity corrections, eV	Corrected μ, eV
**CH_4 (gas)**	-23.34	74.385	53.159	53863	-0.016	-23.356
**CO_2 (gas)**	-23.26	-	-	101325	0	-23.26
**H_2 (gas)**	-6.76	1.089	3.113	3154 (101325)	-0.087 (0)	-6.847 (-6.76)
**H_2O (gas)**	-14.18	-	-	3534[a]	-0.084	-14.264
**CO (gas)**	-15.25	13.398	38.299	38806	-0.024	-15.274
**HCOOH (gas)**	-29.96	-	-	2[a] (101325)	-0.27 (0)	-30.23 (-29.96)
**C_2H_4 (gas)**		6.93	3.302	3345	-0.085	-
**MeOH**		1.554	1.481	1499	-0.105	-
**EtOH**		1.356	0.646	654	-0.126	-

[a] – ref. 46 and used in the paper; Values in () correspond to CHE and adopted in this work; Highlighted rows correspond to products that are not considered in the computational thermodynamic cycle (Fig. 3, main text)

As one can, see the corrections to originally used values of reference chemical potentials are small (< 0.1 eV). Since the optimal path of electrochemical reduction of CO_2 to methane does not include the hydrogen evolution, CO and HCOOH production, the only electrochemical steps that are affected by these corrections are the ones that involve water or methane production:

HO-CO* → CO* + H_2O (ΔG = -0.37 eV vs. -0.29 eV)

CH_3* → CH_4 (ΔG = -0.45 vs. -0.43 eV)

OH* → H_2O (ΔG = +0.66 vs. +0.74 eV)

In Supplementary Figure 26 we show the thermodynamic cycle (CO_2 → CH_4) with chemical potentials corrected for experimental fugacities (Supplementary Table 9)

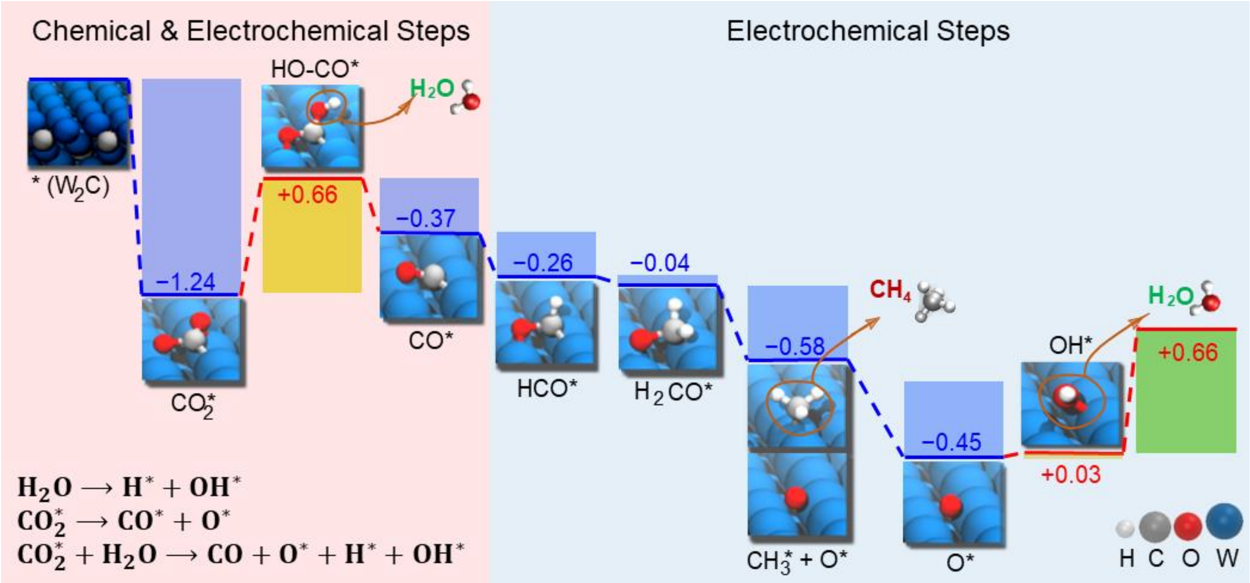

Supplementary Figure 26 | Minimum energy path for the electrochemical CO_2 conversion into CH_4 on the surface of W_2C NFs with corrected free energies due to partial pressure corrections**.** Only electrochemical steps are shown.

From these calculations it is obvious that these corrections are very small and do not change in any appreciable way the estimates of the potentials of limiting steps thus keeping our quantitative and qualitative conclusions the same. Therefore, given the small values of these partial pressure corrections we opted to focus on the dominant electronic part of the chemical potential (with ZPE, enthalpic and entropic corrections at finite temperature) in our calculations.

We use CO_2 reduction pathways devised both for Cu[45] and Mxene[47] to design the CO_2 conversion path on W_2C. Here we limit our analysis to just thermodynamic estimates and do not analyze the kinetics. Thermodynamic reference is made to standard ideal-gas reservoirs of the various reactants and products listed in Supplementary Table 9. For example, adsorption (similarly desorption) energies $B \rightarrow B^*$ are computed as $\Delta G(B \rightarrow B^*) = G(B^*) - G_{surf} - \mu(B)_{gas}$ where the equivalent clean surface free energy G_{surf} is also referenced (see examples in Supplementary Table 9). According to the CHE, the free energies at $\varphi = 0$ vs. RHE of an electrochemical step can be calculated for a reaction $B^* + (H^+ + e^-) \rightarrow BH^*$, using the free energy difference $\Delta G(B^* \rightarrow BH^*) = G(BH^*) - G(B^*) - 1/2\mu(H_2)_{gas}$.

The limiting potential, defined as the least negative potential in which the overall pathway to a product of interest becomes non-endergonic, is calculated using the largest positive change of the free energy (estimated at zero RHE potential) between adjacent steps over the entire pathway (converted to potential by dividing by the number of elementary charge units transferred by the electrode – typically only 1 for individual electrochemical reaction steps). This limiting potential, φ_l, can be viewed as a first approximation to the onset potential for a given product of interest, and thus it serves as an estimate of the potential of the overpotential of the reaction. For similar relevant examples of the application of the CHE method to estimate the overpotential, please refer to works by A. Peterson[45] and references therein.

The full thermodynamic cycle of the CO_2 conversion to methane that contains the lowest free energy pathways, and associated side products produced by electrochemical steps only is shown in Supplementary Figure 27. Depending on the first protonation step, adsorbed CO_2^* may be converted to either $OCHO^*$ or $HO-CO^*$, with the former being the thermodynamically more favorable outcome, with ΔG = -0.091 eV versus ΔG = +0.661 eV for the latter. However, in these cases the direct (chemical) H* transfer steps to $OCHO^*$ or $HO-CO^*$ are thermodynamically more favorable with ΔG = -0.267 eV and ΔG = +0.484 eV, respectively (Supplementary Table 10). The second coupled protonation is an uphill process that may result either in the release of formic acid or production of OCH_2O^*, with the latter being the least endergonic. Again, the steps that are affected by chemical potentials corrections are the following:

HO-CO* → CO* + H_2O (ΔG = -0.37 eV vs. -0.29 eV)
HO-CO* → CO + H_2O (ΔG = +1.06 eV vs. +1.17 eV)
OCH_2O* → HO-CO* + H_2 (ΔG = +0.39 eV vs. +0.48 eV)

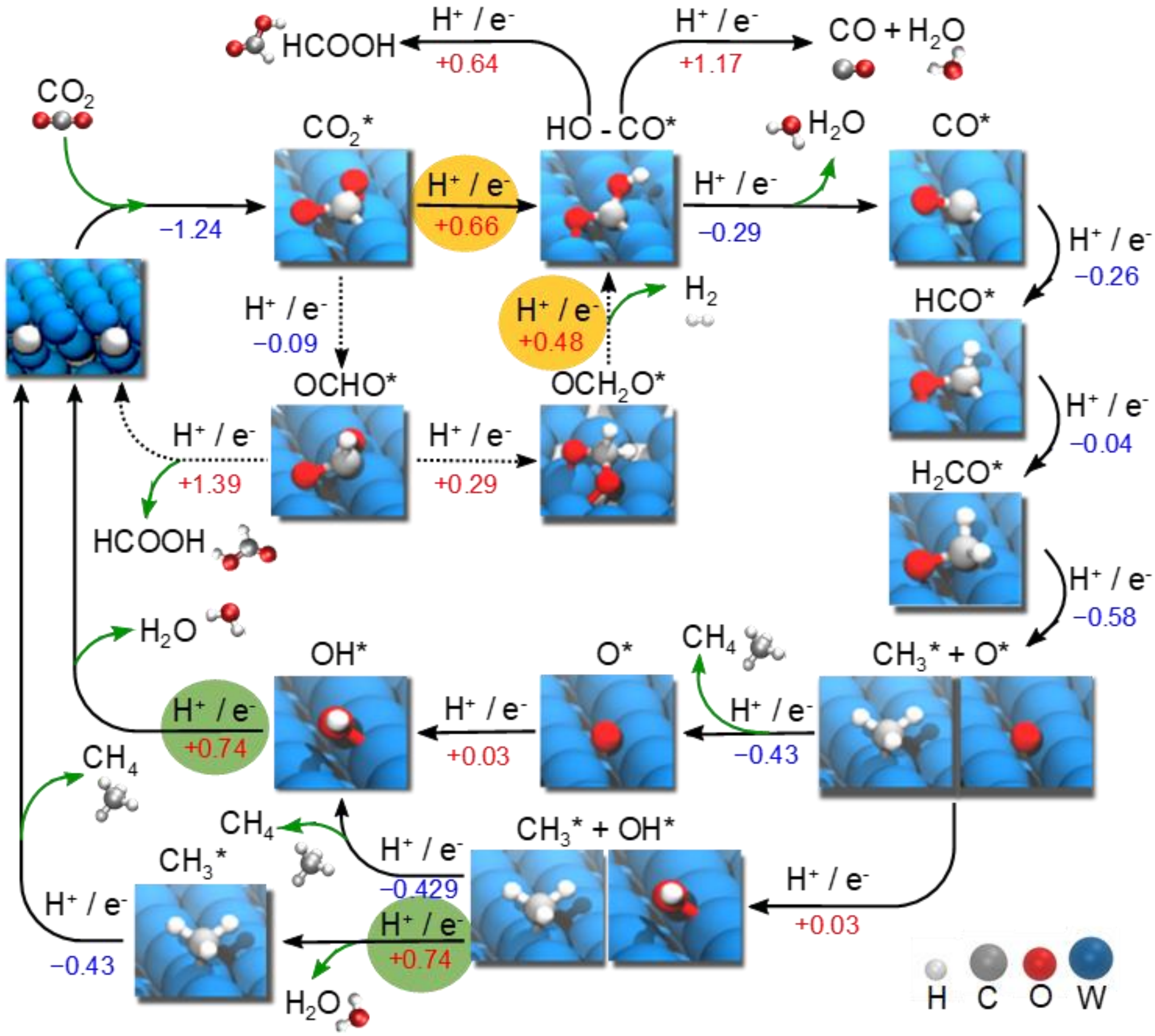

Supplementary Figure 27 | Reaction pathways of the electrochemical conversion of CO_2 on (101) surface of W_2C. Only coupled (H^+ + e^-) transfer steps are shown. Free energies at 0 V vs RHE of steps are shown in eV units (red for uphill and blue for downhill processes). Solid black arrows indicate the "Cu-like" reduction pathway; dotted arrows indicate the "MXene-like" pathway. Green arrows indicate the release of the products into the gas phase. the green and yellow balls indicate the limiting steps for the "Cu-like" and "MXene-like" pathways, respectively.

The third protonation on W_2C leads to the production of H_2 and $HO - CO^*$ which brings us back onto the "Cu-like" path of reduction. However, even though the next protonation towards CO* on W_2C is similar to that on Cu or Mo_2C, the following step remains still downhill with ΔG = -0.256 eV, whereas, on Cu, the conversion $CO^* + H^+ + e^- \rightarrow HCO^*$ is the rate-limiting uphill process with a limiting potential ranging from -0.74 V to -0.97 V vs RHE[45]. The activation of CO^* on Mo_2C is easier (ΔG = +0.38 eV) [48], but also remains an uphill process. Moreover, due to the spontaneous

water dissociation, the direct H* transfer step $CO^* + H^* \rightarrow HCO^*$ on W_2C could be even more favorable with the outcome ΔG= -0.433 eV (Supplementary Table 10). Another important difference between Cu, or MXenes, and W_2C is that the methoxy radical CH_3O^* with oxygen attached to the surface W atom is not stable and spontaneously dissociates into the methyl radical CH_3^* and the surface oxygen atom O^*. Similarly, to Mo_2C, the protonation of OH^* is the limiting step of the overall reaction $CO_2^* + 8\,H^+/\,e^- \rightarrow * + 2H_2O + CH_4$ (where the lone * refers to the clean surface). However, on W_2C (101), the limiting potential of -0.744 V vs RHE is significantly smaller than the reported value of -1.49 V for Mo_2C (100) [48]. The value of -0.744 V sets the upper boundary for the limiting potential obtained at the low surface coverage limit. However, as shown by previous studies on Mo_2C [48], the stability of some surface species, including OH*, can be significantly smaller at increasing coverage, thus reducing the limiting potential. On the other hand, the ease of water dissociation on W_2C (101) makes OH^* a "poisoning" surface agent that will compete with other surface reactions. In this case, the limiting potential can be determined by other reactions (e.g., $CO_2^* + 8\,H^+/\,e^- \rightarrow H_2 + H_2O + CH_4 + O^*$) and can be as small as -0.483 V vs RHE (Supplementary Figures 28-31).

In contrast to Cu where the desorption of CO* is a relatively easy process (the estimated limiting potential is -0.41 V vs RHE) and the CO desorption itself requires *ca.* 0.3 eV[45,49], the desorption of CO* from W_2C (101) requires 1.46 eV. This contradicts the CO production and cannot explain its small overpotential (*ca.* 12.7 mV). Therefore, our calculations suggest that the electrocatalytic performance of W_2C (101) towards CO_2 reduction may be strongly influenced by the competition of electrochemical and chemical reactions and the availability of catalytic sites on the catalyst surface. Specifically, the exergonic chemical dissociation of CO_2 (Supplementary Table 10) and the spontaneous adsorption-dissociation of water, acting in concert, may drive the massive CO production ($CO_2^* + H_2O \rightarrow CO + O^* + H^* + OH^*,\ \Delta G = -0.736\ eV$) characterized by two jumps at -0.450 V and -0.750 V vs RHE, respectively (Supplementary Table 10). This coupling

makes the CO formation weakly dependent on the electrode potential which is also confirmed by our three-electrode electrochemical cell results where a similar trend is observed for CO production on W_2C NFs (Supplementary Table 1). The partial CO current (Supplementary Figure 3) also demonstrates a much weaker dependence of its kinetics on the electrode potential as compared to Cu which suggests that chemical steps play a relevant role in the CO production. For the same reason, the equilibrium of the reaction $CO^* + H_2O \rightarrow CO + H^* + OH^*$ depends on the reaction $OH^* + H^+/\, e^- \rightarrow H_2O,$ which is dependent on the electrode potentials. Therefore, at more negative electrode potentials, the equilibrium will be shifted to the bound CO* which limits the formation of CO at high cathodic potentials and is indeed observed experimentally (Supplementary Table 1).

In Supplementary Figures 28-31 we show free energies of the possible reactions at $\varphi = 0$ vs. RHE and at various limiting potentials φ_l. The essential chemical steps and their free energies are listed in Supplementary Table 10.

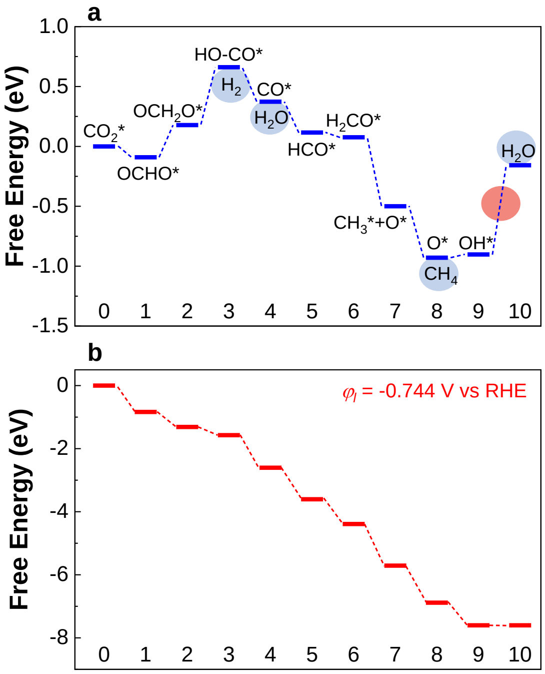

Supplementary Figure 28 | The lowest free energy path of conversion of CO_2 on (101) W_2C slab at **(a)** $\varphi = 0$ and at **(b)** φ_l = -0.744 V vs RHE. The red ball marks the limiting step. Numbers 0 … 10 correspond to the total number of electrons/protons transferred from the electrode/solvent. Blue balls indicate gas-phase products.

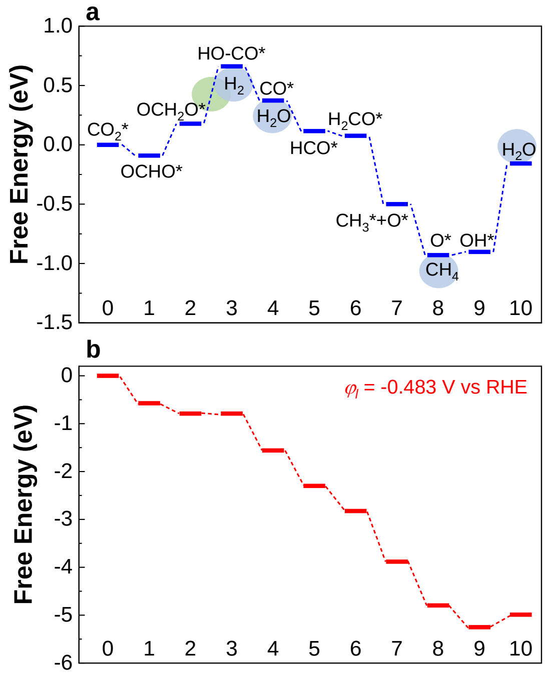

Supplementary Figure 29 | The lowest free energy path of conversion of CO_2 on (101) W_2C slab at **(a)** $\varphi = 0$ and at **(b)** φ_l = -0.483 V vs RHE. The green ball marks the limiting step. Numbers 0 … 10 correspond to the total number of electrons/protons transferred from the electrode/solvent. Blue balls indicate gas-phase products.

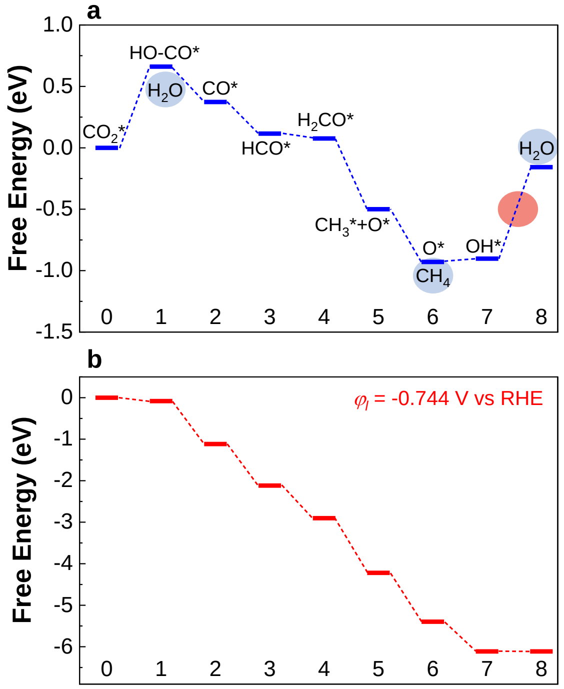

Supplementary Figure 30 | The "Cu-like" free energy path of conversion of CO_2 on (101) W_2C slab at **(a)** $\varphi = 0$ and at **(b)** φ_l = -0.744 V vs RHE. The red ball marks the limiting step. Numbers 0 … 8 correspond to the total number of electrons/protons transferred from the electrode/solvent. Blue balls indicate gas-phase products.

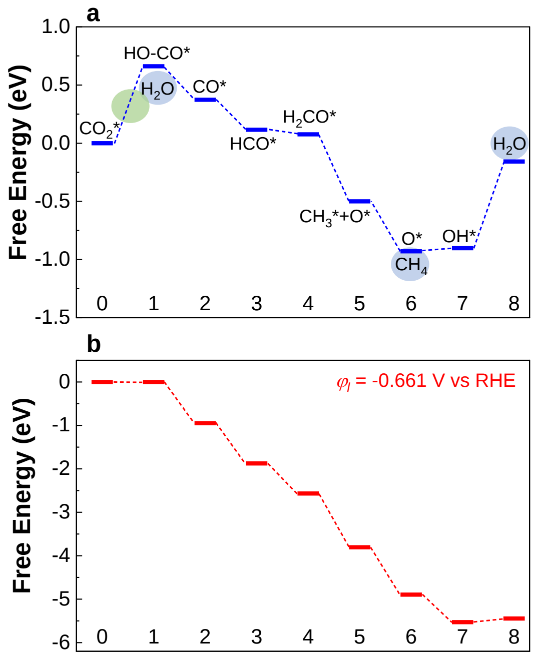

Supplementary Figure 31 | The "Cu-like" free energy path of conversion of CO_2 on (101) W_2C slab at **(a)** $\varphi = 0$ and **(b)** at φ_l = -0.661 V vs RHE. The green ball marks the limiting step. Numbers 0 … 8 correspond to the total number of electrons/protons transferred from the electrode/solvent. Blue balls indicate gas-phase products.

Supplementary Table 10 | Chemical steps on W_2C (101) and their free energies

Reaction	ΔG (eV)
$CO_2 \rightarrow CO_2^*$, adsorption	-1.242
$H_2O \rightarrow H^* + OH^*$, adsorption + dissociation	-1.712 (-1.796)
$CO_2^* \rightarrow CO^* + O^*$, dissociation	-0.971
$CO^* \rightarrow CO$, desorption	+1.436 (+1.460)
$CO_2^* + H^* \rightarrow HO-CO^*$, H* transfer step	+0.484
$CO_2^* + H^* \rightarrow OCHO^*$, H* transfer step	-0.267
$CO^* + H^* \rightarrow HCO^*$, H* transfer step	-0.433
$CO^* + H_2O \rightarrow CO + H^* + OH^*$, desorption assisted by water adsorption	-0.277 (-0.336)
$O^* + H_2O \rightarrow 2OH^*$, protonation of surface oxygen	-0.632 (-0.716)
$CO_2^* + H_2O \rightarrow CO + O^* + H^* + OH^*$, desorption following dissociation of CO_2^*	-0.676 (-0.736)

The values corrected by experimental fugacities are shown by red.

Supplementary Table 11 | Adsorption energies with respect to gas phase references and ***ΔG*** of the limiting step reaction for TMCs with indications of spontaneous (diss) or exergonic dissociation of adsorbed species.

	H_2O	CO_2	$\Delta G,\ OH^* + H^+ + e^- \rightarrow H_2O$
**W_2C (101)**	-1.796 eV (diss)	-1.24 eV	+0.74 eV
**Nb_2C (101)**	-1.87 eV (diss)	-1.32 eV	+1.17 eV
**Mo_2C (101)**	-1.23 eV	-1.62 eV	+1.25 eV
**V_2C (101)**	-0.59 eV	-0.95 eV	+0.85 eV

Since CO evolution is an important part of the selective CO_2 reduction on W_2C, here we discuss the pathways of CO production. In Supplementary Figure 32 we show the electrochemical steps for CO production.

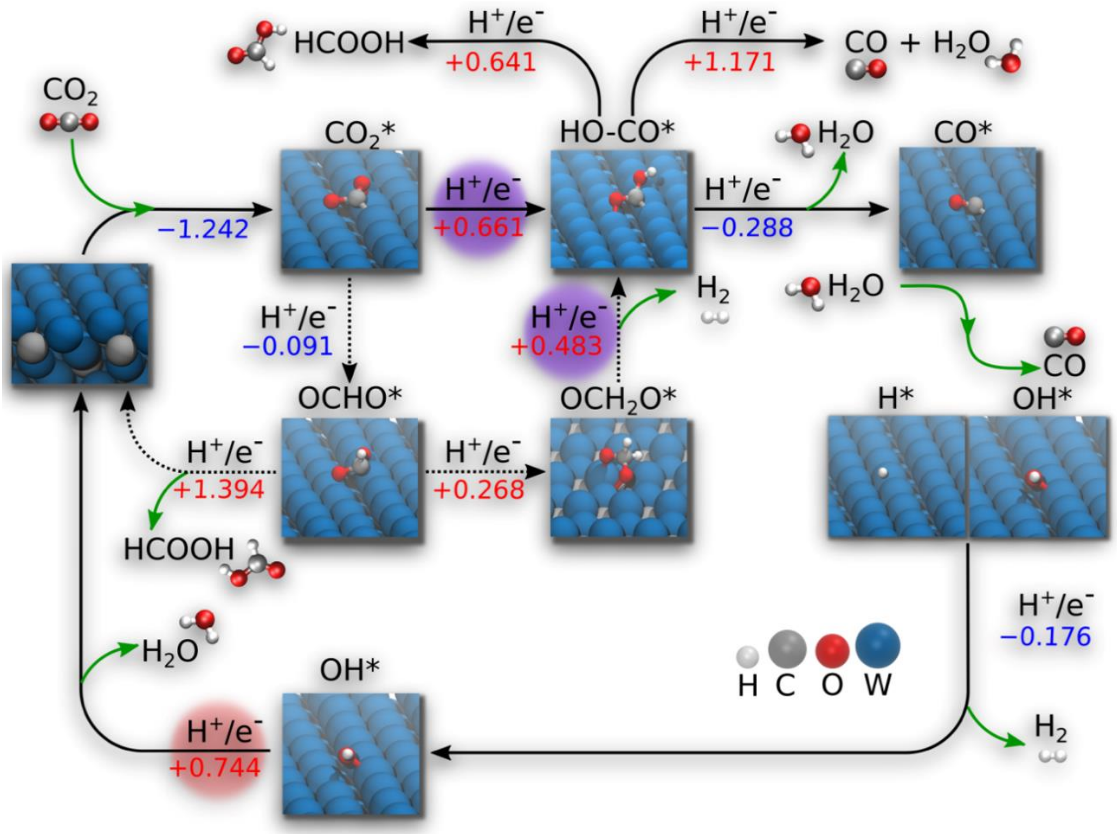

Supplementary Figure 32 | Reaction pathways, a combination of electrochemical (black arrows) and chemical steps (green arrows) of the electrochemical conversion of CO_2 to CO on the (101) surface of W_2C. Free energy differences between steps are shown in eV units (red for uphill and blue for downhill processes). Solid black arrows indicate the "Cu-like" reduction pathway; dotted arrows indicate the "Mxene-like" pathway. Green arrows indicate the release of the products into the gas phase. Red and magenta balls mark the limiting steps for the "Cu-like" and "TMC-like" pathways, respectively.

In Supplementary Figures 33-36, we show free energies of the possible reactions of conversion of CO_2 to CO at $\varphi = 0$ vs. RHE and at various limiting potentials φ_l for pathways that combine chemical and electrochemical steps.

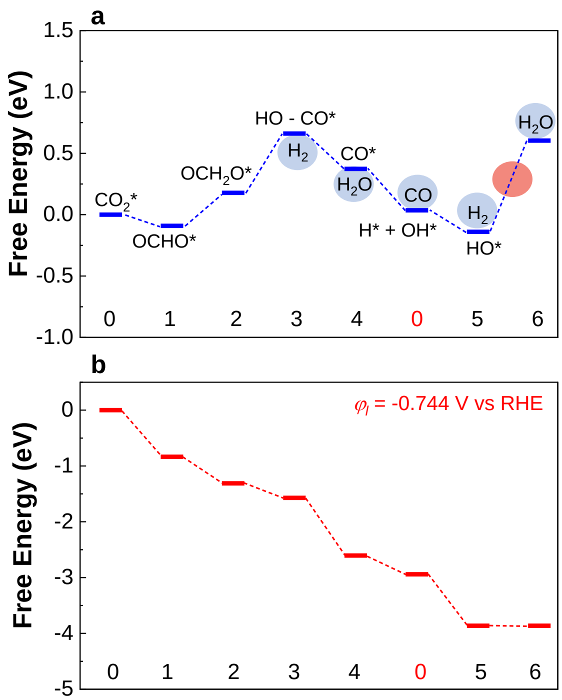

Supplementary Figure 33 | The lowest free energy path of conversion of CO_2 to CO on (101) W_2C slab at **(a)** $\varphi = 0$ and at **(b)** φ_l= -0.744 V vs RHE. The red ball marks the limiting step. Numbers 0 … 6 correspond to the total number of electrons/protons transferred from electrode/solvent. Blue balls indicate gas-phase products.

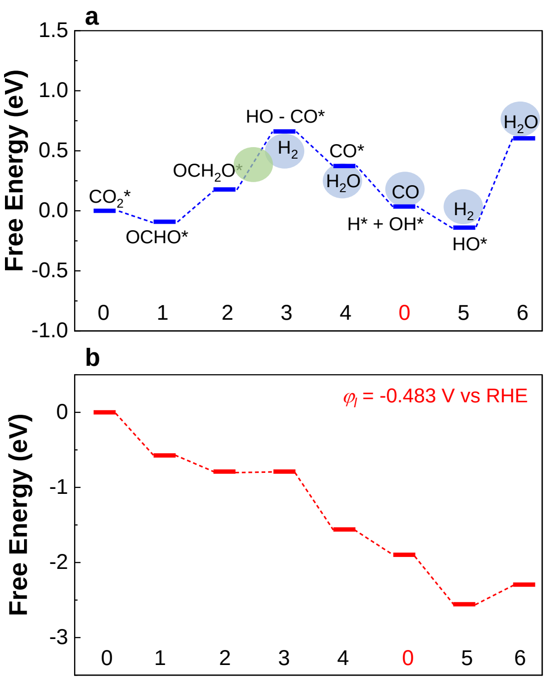

Supplementary Figure 34 | The lowest free energy path of conversion of CO_2 to CO on (101) W_2C slab at **(a)** $\varphi = 0$ and at **(b)** φ_l = -0.483 V vs RHE. The green ball marks the limiting step. Numbers 0 … 6 correspond to the total number of electrons/protons transferred from the electrode/solvent. Blue balls indicate gas-phase products.

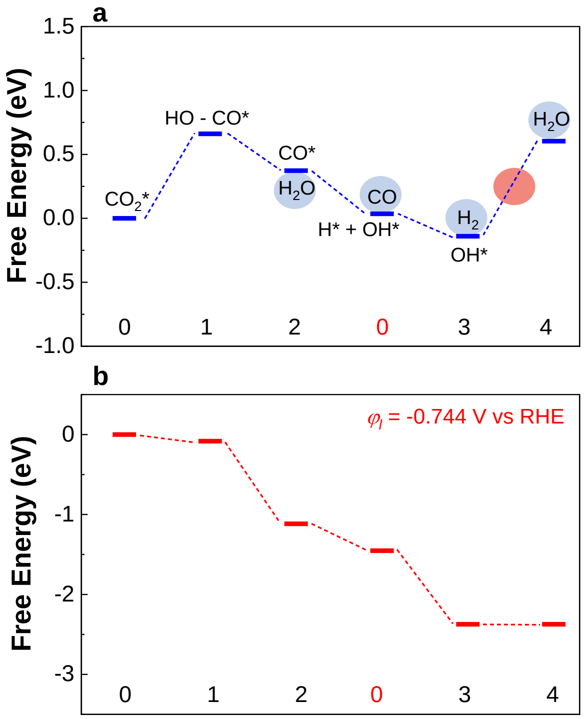

Supplementary Figure 35 | The "Cu-like" free energy path of conversion of CO_2 to CO on (101) W_2C slab at **(a)** $\varphi = 0$ and at **(b)** φ_l = -0.744 V vs RHE. The red ball marks the limiting step. Numbers 0 … 4 correspond to the total number of electrons/protons transferred from the electrode/solvent. Blue balls indicate gas-phase products.

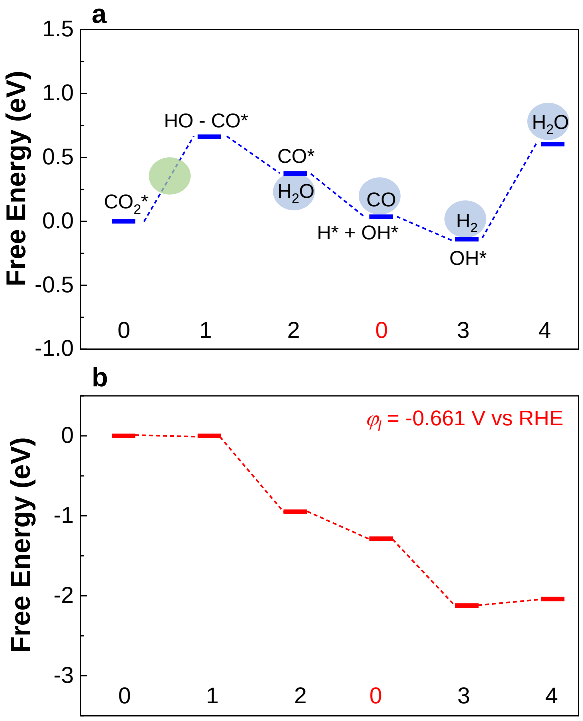

Supplementary Figure 36 | The "Cu-like" free energy path of conversion of CO_2 to CO on (101) W_2C slab at **(a)** $\varphi = 0$ and at **(b)** φ_l = -0.661 V vs RHE. The green ball marks the limiting step. Numbers 0 … 4 correspond to the total number of electrons/protons transferred from the electrode/solvent. Blue balls indicate gas-phase products.

14- Effect of Choline Chloride on the Electrochemical Performance of TMCs

To investigate the dependence of activity and selectivity of W_2C NFs for electrochemical CO_2RR on varying concentration of choline chloride, we performed CO_2RR using mixed 3 M KOH with different concentrations (0, 0.01, 0.1, 1 and 2 M) of choline chloride (CC). All experiments were performed in the three-electrode electrochemical cell under identical conditions. Prior to each experiment, the cell was first bubbled with Ar and then with CO_2, each for 60 min at the flow rate of 50 ml/cm^2. The chronoamperometry experiments were performed for the specific amount of time at different potentials. The products of the electrochemical experiments characterized using gas chromatography (GC) and differential electrochemical mass spectroscopy (DEMS). The electrochemical results of W_2C NFs in different concentrations of mixed 3 M KOH and choline chloride electrolytes are shown in Supplementary Figure 37. Supplementary Figure 37a indicates the current densities of W_2C NFs in the different electrolytes. As shown in this figure, adding choline chloride to the 3 M KOH electrolyte increases total activity of W_2C NFs in the

electrochemical CO_2RR. The overall current density and Faradaic efficiency (FE) results of W_2C NFs in studied electrolytes are represented in Supplementary Figures 37a-f. These results reveal that increasing concentration of choline chloride enhances the electrochemical CO_2RR and suppresses the competing hydrogen evolution reaction.

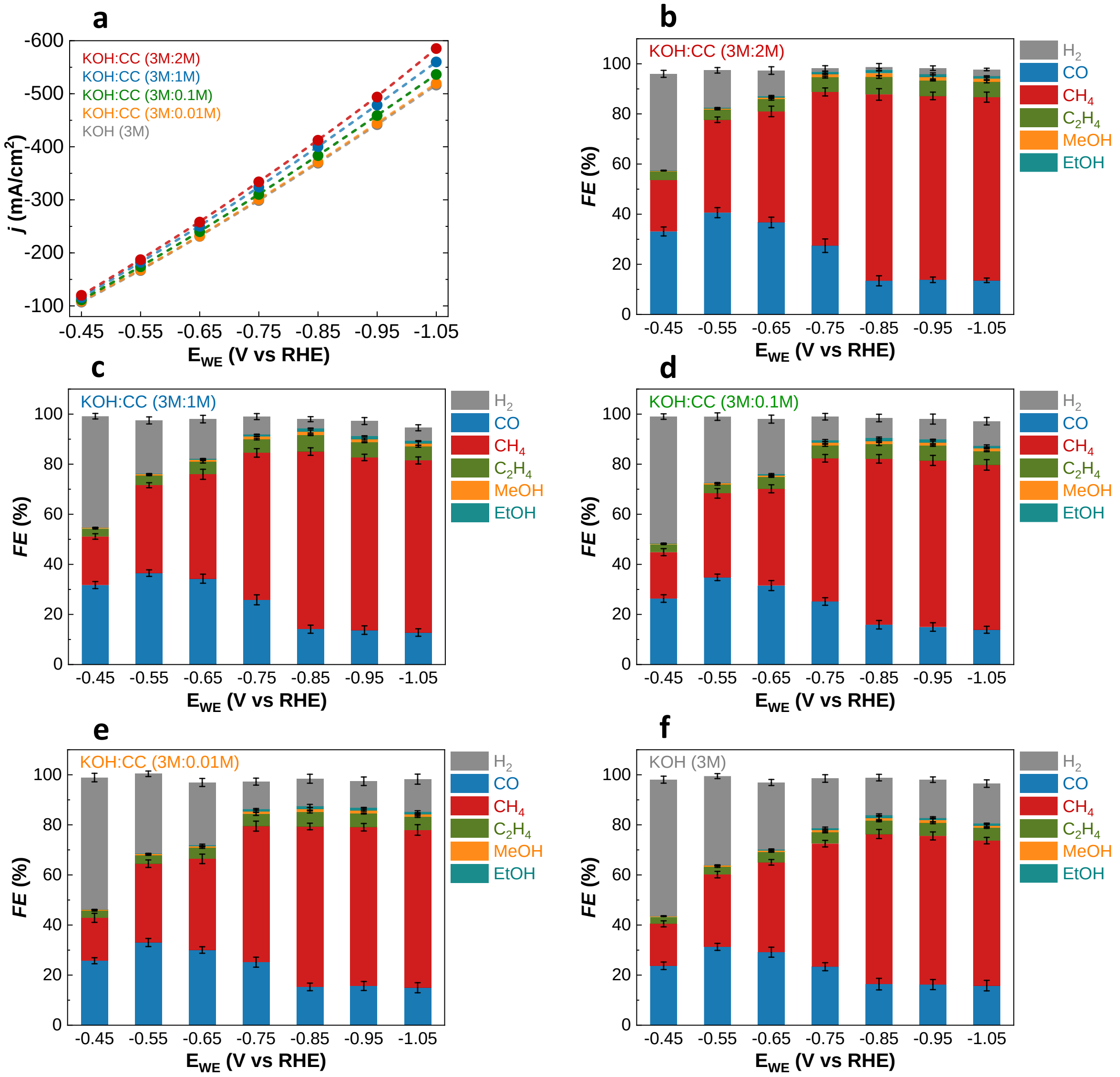

Supplementary Figure 37 | Effect of choline chloride (CC) in electrochemical performance of W_2C NFs for CO_2RR. **(a)** Current density as a function of potential for W_2C NFs using different concentrations of choline chloride obtained by chronoamperometry experiments at identical experimental conditions. **(b-f)** Faradaic efficiency (FE) measurements of H_2, CO, CH_4, C_2H_4, methanol (MeOH) and ethanol (EtOH) for W_2C NFs using mixed 3 M KOH and different concentrations of choline chloride (0, 0.01, 0.1, 1 and 2 M).

15- Stability Analysis of Choline Chloride Electrolyte using NMR experiments

We performed nuclear magnetic resonance (NMR) experiments for fresh and used studied electrolytes to examine the stability of choline chloride in the electrochemical CO_2RR. At first, we purged Argon (ultra-high purity grade, Airgas) into the electrolytes with a flow rate of 50 mL/min for 60 min to remove all dissolved gassed from the electrolyte. Then used samples were collected by performing chronoamperometry experiments at a cell potential of -1.05 V for 120 minutes. The NMR samples were prepared by mixing 0.1 mL of each electrolyte and 0.5 mL of D_2O solvent. ^{1}H NMR and ^{13}C NMR experiments were carried out with a Bruker Advance III 500 MHz system equipped with DCH cryoprobe. All experiments were carried out at 298 K.

^{1}H NMR spectra of fresh and used electrolytes are shown in Supplementary Figure 38. The recorded ^{1}H spectra exhibit 5 signals in the aliphatic region for the alkyl chains. The peaks at 3.04 ppm correspond to $-CH_3$ groups which are attached to nitrogen for fresh and used electrolytes. Furthermore, the peaks at 3.26 and 3.87 ppm represent $-CH_2$ groups next to nitrogen and oxygen, respectively. The solvent peak ($H_2O + D_2O$) overlaps with the -OH peak and appears at a chemical shift of 4.72 ppm. Moreover, the results for ^{13}C NMR are shown in Supplementary Figure 39. In all spectra three distinguished peaks of choline chloride were observed. The peak located at 53.71 ppm corresponds to s $-CH_3$ group whereas the $-CH_2$ group peaks appeared at 55.79 and 68.71 ppm. The ^{1}H and ^{13}C NMR spectra reveal similar peak areas and chemical shifts of $-CH_2$ and -CH_3 peaks for fresh and used electrolytes that confirm no generation of new diamagnetic species and change in the choline chloride structure and thus verify high stability under applied potential of -1.05 V vs RHE.

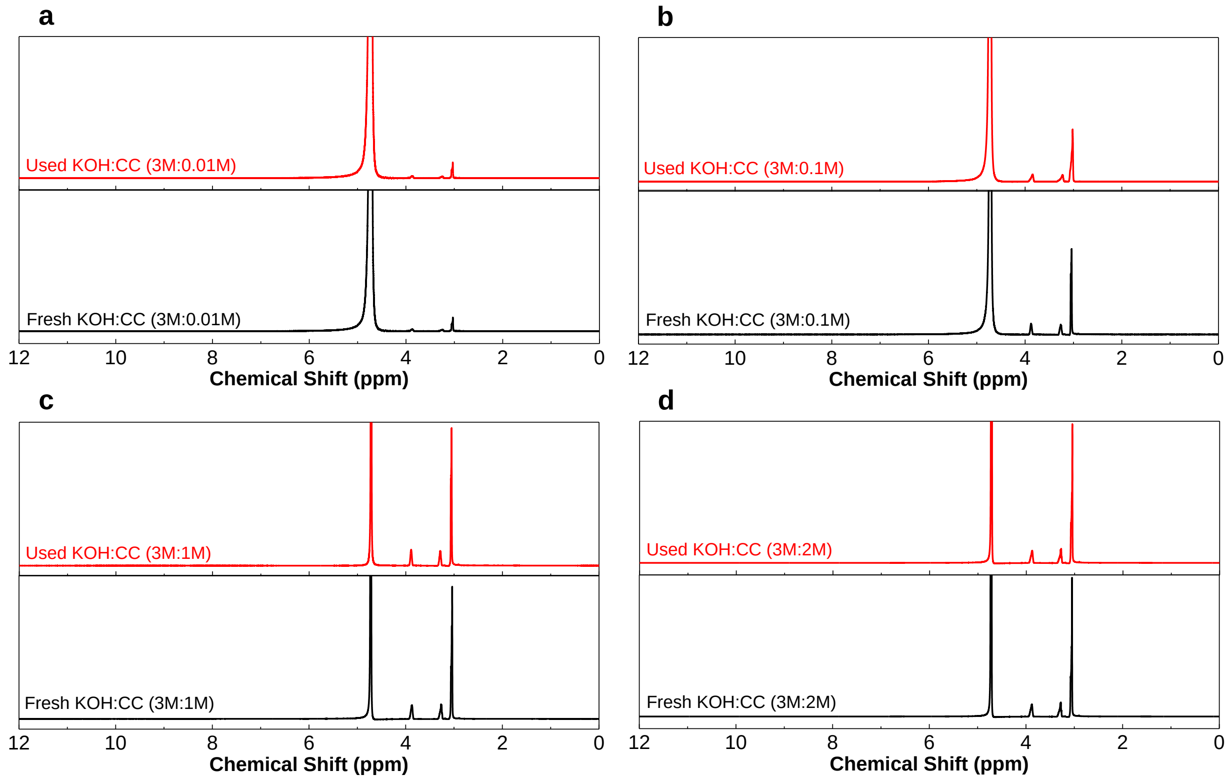

Supplementary Figure 38 | ^{1}H Nuclear Magnetic Resonance (NMR) results of fresh and used electrolytes **(a)** KOH:CC (3M:0.01M) **(b)** KOH:CC (3M:0.1M) **(c)** KOH:CC (3M:1M) **(d)** KOH:CC (3M:2M).

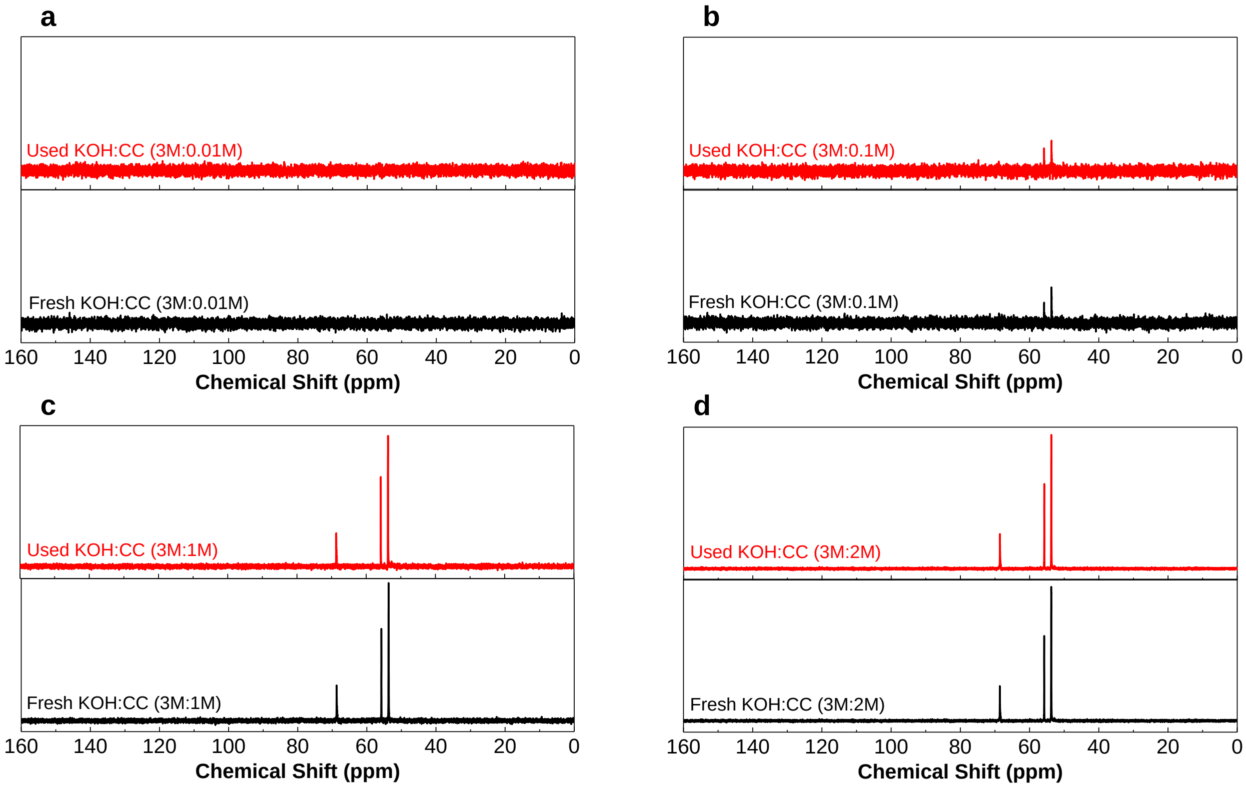

Supplementary Figure 39 | ^{13}C Nuclear Magnetic Resonance (NMR) results of fresh and used electrolytes **(a)** KOH:CC (3M:0.01M) **(b)** KOH:CC (3M:0.1M) **(c)** KOH:CC (3M:1M) **(d)** KOH:CC (3M:2M).

16- 13-C Labeled Carbon Dioxide ($^{13}CO_2$) Experiments

We have performed electrochemical CO_2RR experiments using 13-C labeled CO_2 ($^{13}CO_2$) to verify the source of carbon for products such as CH_4 in our system. To do this, a custom-made two-compartment three-electrode electrochemical cell filled with the KOH:CC (3M:2M) solution was used to perform the electrochemical CO_2RR. The working electrode (cathode) was prepared by drop casting the synthesized W_2C NFs on a customized glassy carbon (GC) electrode. Platinum (Pt) gauze 52 mesh (Alfa Aesar) and Ag/AgCl (BASi) were used as counter and reference electrodes, respectively. The cathode and anode parts of the cell were separated by a frit to eliminate the effect of oxidation product at the anode surface. The cell was carefully sealed to prevent any leakage from the head space of the cell. At first, we purged Argon (ultra-high purity grade, Airgas) into the solution with a flow rate of 30 mL/min for 20 min to remove all dissolved gases from the electrolyte. The $^{13}CO_2$ (Sigma Aldrich) was bubbled into the cell to saturate the electrolyte. Then, we performed the chronoamperometry (CA) experiment at a potential of -0.85 V vs RHE (Supplementary Figure 40). Once the CA experiment was finished, 2 mL sample from the headspace of the electrochemical cell was collected using a sample locked syringe (Hamilton) and then injected into the differential electrochemical mass spectrometer (DEMS, HPR-40, Hiden Analytical) to analyze the type of products in the cell. These mass spectrometer settings were optimized to detect any possible products with partial pressures as low as 1.0 x 10^{-13} Torr. We used bar detection mode to measure peak intensities from mass to charge (m/z) ratio from 1 to 60 with the scan rate of 0.2 second per scan (Supplementary Figure 41). All recorded data were analyzed by MASoft 7 professional software (Hiden Analytical).

The DEMS bar mode analysis of the 2 mL sample is shown in Supplementary Figure 41. The result of the Supplementary Figures 41a and b show the DEMS bar diagram results for after and before 2 mL sample injection, respectively. The final data obtained by subtracting DEMS results before and after the sample injection are also shown in Supplementary Figure 41c, indicating 13-C labeled hydrocarbon products at different m/z ratios. As shown in this figure, two peaks with

relatively high intensities are observed at m/z=17 and m/z=29, corresponding to the formation of $^{13}CH_4$ and ^{13}CO hydrocarbon products. Supplementary Figure 41c also shows ^{13}C (m/z=13), $^{13}C_2H_4$ (m/z=30), $^{13}CH_3OH$ (m/z=33), $^{13}CO_2$ (m/z=45) and $^{13}C_2H_5OH$ (m/z=48) peaks, suggesting formation of other hydrocarbon products. Moreover, Supplementary Figure 41c does not show any peaks at m/z=12, 16, 28, 32 and 46 signals (corresponding to ^{12}C, $^{12}CH_4$, ^{12}CO, $^{12}C_2H_4$, $^{12}CH_3OH$ and $^{12}C_2H_5OH$, respectively), confirming that the injected 13-C labeled CO_2 is the only source of the products.

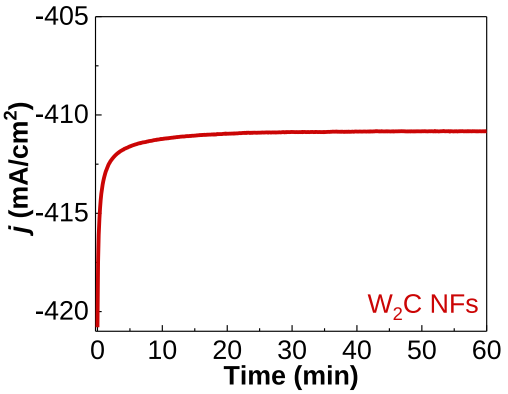

Supplementary Figure 40 | The chronoamperometry (CA) experiment results for W_2C NFs in the two-compartment three-electrode electrochemical cell using $^{13}CO_2$ saturated KOH:CC (3M:2M) electrolyte.

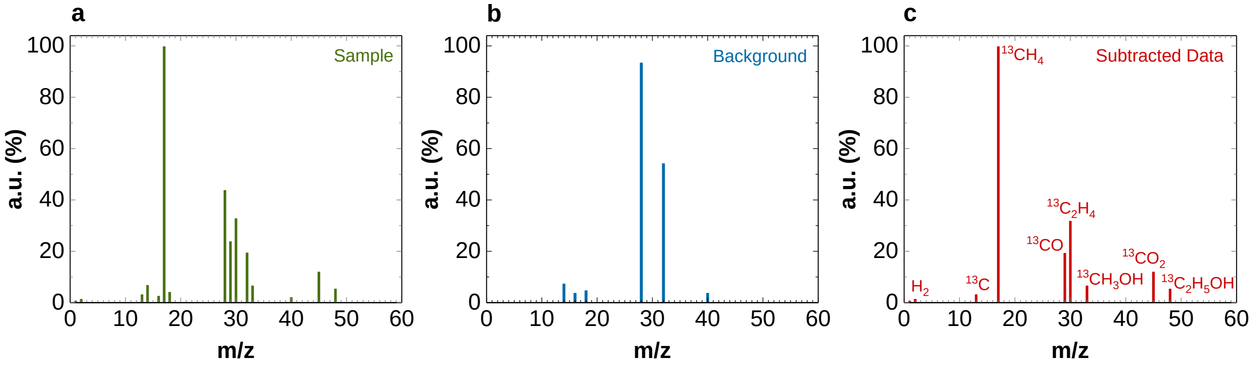

Supplementary Figure 41 | Mass spectroscopy analysis of 2 mL gas sample extracted from sealed two-compartment three-electrode cell. m/z shows mass-to-charge ratio. **(a)** Raw sample data after injecting 2 mL sample to DEMS **(b)** Background data **(c)** Deconvoluted data which is derived from subtracting raw sample data from background data.

To further confirm this, we also measured the partial pressure variation of the possible products as a function of time using the multiple ion detection (MID) mode. This allows us to precisely determine the source of possible formed 13-C labeled hydrocarbon products ($^{13}CH_4$, ^{13}CO, $^{13}C_2H_4$, $^{13}CH_3OH$, $^{13}CO_2$ and $^{13}C_2H_5OH$) in this study. To do this, specific m/z ratios were chosen for 13-

C products. In detail, m/z ratios of 17, 29, 30, 33, 45 and 48 were selected for detection of $^{13}CH_4$ (m/z=17), ^{13}CO (m/z=29), $^{13}C_2H_4$ (m/z=30), $^{13}CH_3OH$ (m/z=33), $^{13}CO_2$ (m/z=45) and $^{13}C_2H_5OH$ (m/z=48) respectively. Supplementary Figures 42a-f show the results of MID mode spectra obtained by injecting 2 mL sample from the headspace of the cell to the DEMS using a sample locked syringe (Hamilton) after the chronoamperometry experiments. As shown in these figures, immediate signal increase is observed for different m/z ratios after the sample injection, confirming that the only source of 13-C labeled formation of products is the $^{13}CO_2$ gas.

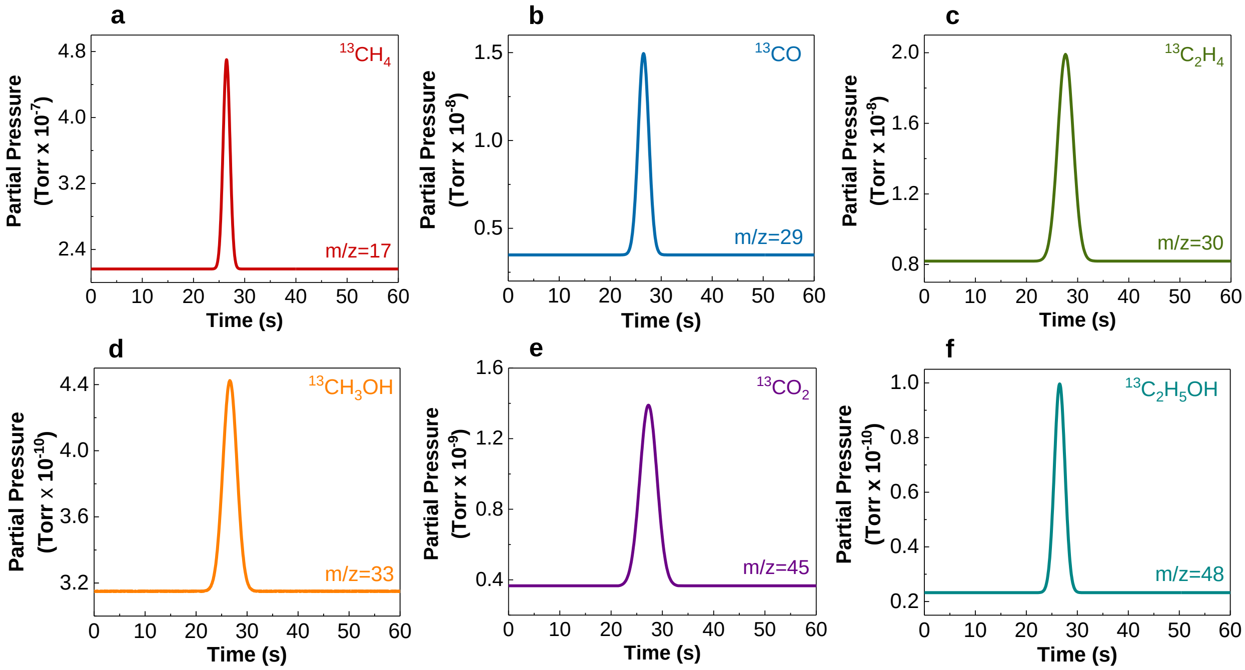

Supplementary Figure 42 | The multiple ion detection (MID) signals of **(a)** $^{13}CH_4$, **(b)** ^{13}CO, **(c)** $^{13}C_2H_4$, **(d)** $^{13}CH_3OH$, **(e)** $^{13}CO_2$ and **(f)** $^{13}C_2H_5OH$ obtained in a CA experiment using W_2C NFs in $^{13}CO_2$-saturated KOH:CC (3M:2M) electrolyte in the two-compartment three-electrode cell.

17- Continuous Flow Electrolyzer Experiments: Methods and Materials

A solid polymer electrolyte flow electrolyzer was used to study electrochemical performance of W_2C NFs, Au NPs and Cu NPs for continuous electrochemical CO_2 reduction reaction. The photos of the flow electrolyzer and different compartments are shown in Supplementary Figure 43 and 44. As shown in these figures, the working and counter electrodes with geometric surface areas of 5 cm^2 were separated using an anion exchange membrane (Sustainion X37-50 Grade RT, Dioxide Materials). A peristaltic pump (Masterflex, Cole-Parmer) was used to continuously pump

KOH:CC (3M:2M) (pH = 14.5) with flow rate of 20 ml/min through the anode side. A mass flow controller (SmartTrak 50, Sierra, calibrated with CO_2 gas) connected to the humidifier kit, was used to set the flow rate of CO_2 at 50 ml/min. The membrane was treated in the electrolyte for 12 hr at 75 °C prior to use.

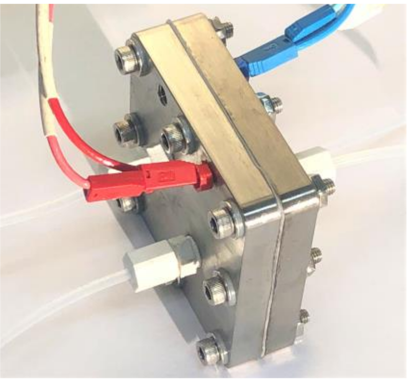

Supplementary Figure 43 | Photo of the flow electrolyzer used for electrochemical CO_2RR testing. The membrane in the flow electrolyzer consists of the working electrode (W_2C NFs, Au NPs and Cu NPs) and counter electrode (IrO_2 NPs) on either side of the anion exchange membrane (AEM).

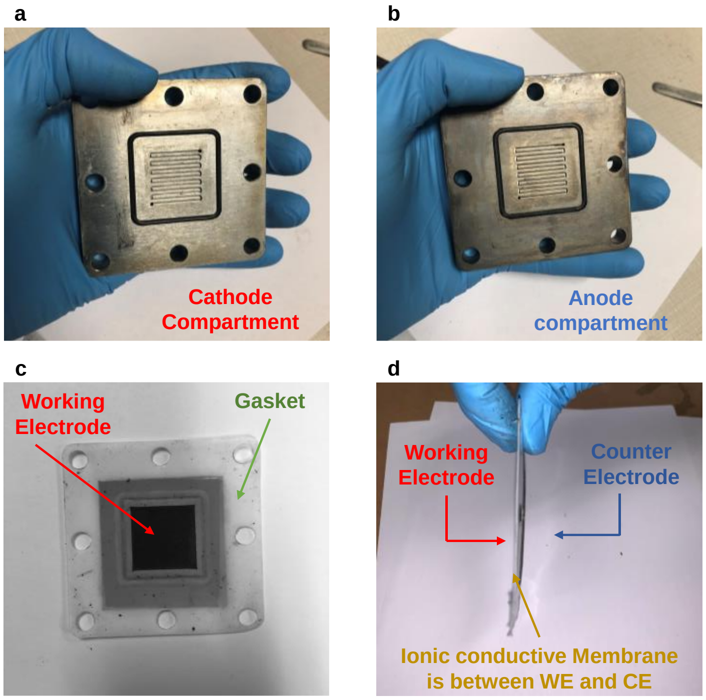

Supplementary Figure 44 | The pictures of different parts of the flow electrolyzer.

The cathode electrodes were prepared by mixing 5 mg of each catalysts (W_2C NFs, Au NPs and Cu NPs) with 20 ml of isopropyl alcohol in a glass vial. The mixed inks were then sonicated (Branson 2800 ultrasonic processor, Cleanosonic) for 30 minutes and hand-brush coated on gas

diffusion layers (GDLs, Sigracet 39, Fuel Cell Store). The actual loadings of 0.1±0.01 mg.cm^{-2} was determined by weighting the GDLs before and after deposition. IrO_2 powder (Sigma Aldrich) was used to prepare anodes following the same method.

The chronoamperometry experiments were performed to study the performance of W_2C NFs, Au NPs and Cu NPs in the flow electrolyzer by applying different cell potentials (potential difference between cathode and anode) and simultaneously reading the current. The current densities of different catalysts in the flow cell at cell potentials of -1.5 to -2.3 V are presented in Supplementary Figure 45. The figure presents average current densities of one-hour experiments at different cell potentials for the studied catalysts.

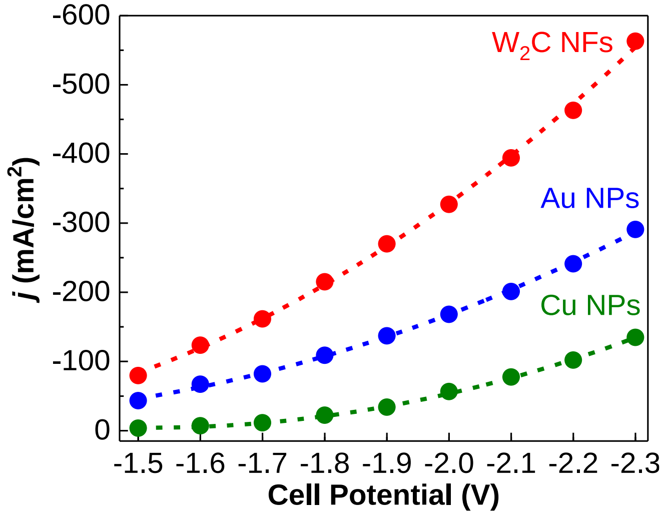

Supplementary Figure 45 | Current density as function of cell potential for W_2C NFs compare to Au and Cu nanoparticles, obtained by chronoamperometry experiments at identical experimental conditions.

The GC and *in situ* DEMS experiments was used to analyze the products of electrochemical CO_2RR in the solid polymer electrolyte flow electrolyzer. The FE values at different cell potentials were obtained using the Equation 2. Supplementary Table 12 indicates FE measurements of W_2C NFs for different products i.e., H_2, CO, CH_4, C_2H_4, CH_3OH and C_2H_5OH in the solid polymer flow electrolyzer at different cell potentials.

Supplementary Table 12 | Faradaic efficiency measurements of W_2C NFs in the solid polymer electrolyte flow electrolyzer at different cell potentials.

Cell Potential (V)	FE_{H2} (%)	FE_{CO} (%)	FE_{CH4} (%)	FE_{C2H4} (%)	FE_{MeOH} (%)	FE_{EtOH}(%)
-1.5	54.8576	40.02105				
-1.6	32.5712	43.20585	19.17932	2.13547	0.03226	
-1.7	11.1072	50.88655	32.35172	3.08662	0.31283	0.07985
-1.8	9.3454	41.0924	42.70344	3.67747	0.45381	0.17725
-1.9	8.46437	28.3193	55.94484	4.46296	0.60062	0.45083
-2	4.4288	14.79415	72.64828	5.29993	1.1147	0.74493
-2.1	1.3568	6.02105	82.70344	6.30677	1.38742	1.20951
-2.2	5.7504	4.65965	78.93792	5.61597	1.26225	1.08218
-2.3	12.928	1.7731	74.82068	4.85084	1.05752	0.92223

Supplementary Figure 46 shows the FE measurements of Au and Cu NPs for CO_2RR in the solid polymer flow electrolyzer at different cell potentials.

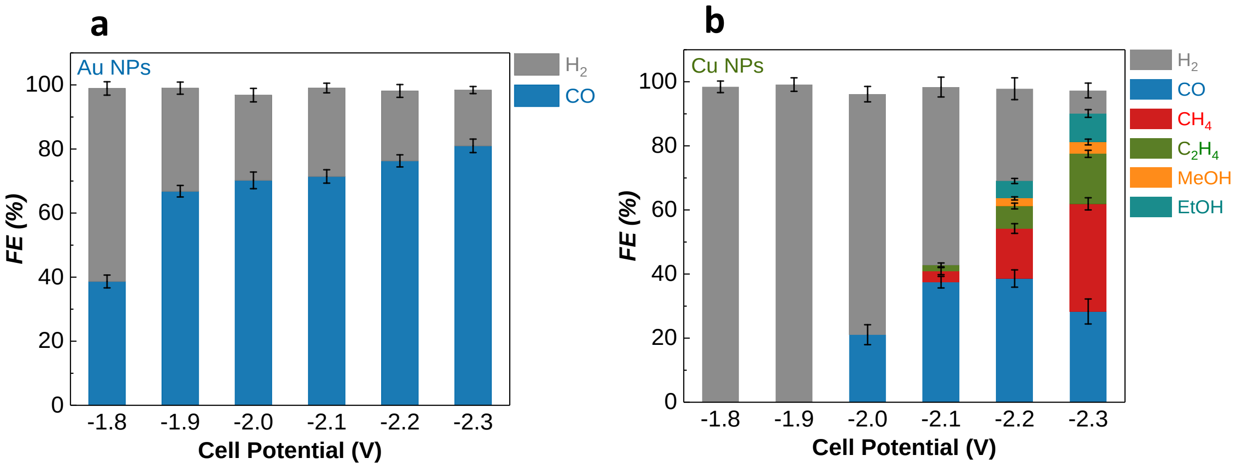

Supplementary Figure 46 | Faraday Efficiency measurements of **(a)** Au NPs **(b)** Cu NPs for CO_2RR in solid polymer electrolyte flow electrolyzer at different cell potentials.

The efficiency of CO_2RR using W_2C NFs in the solid polymer electrolyte flow electrolyzer at different applied cell potentials was measured using Equation 11.

$$CO_2RR\ Efficiency\ (\%) = \frac{Output\ Energy}{Input\ Energy} \times 100 = \frac{Power\ of\ Products\ (W/cm^2)}{Cell\ Power\ Density\ (W/cm^2)} \times 100 \quad \text{(Equation 11)}$$

Where the power of CO_2RR products is obtained by multiplying the rate of products formation and lower heating values (LHV) of products. The LHV of CO, CH_4, C_2H_4, CH_3OH and C_2H_5OH are 282.98, 802.23, 1322.94, 638.73 and 1235.45 (KJ/mol), respectively. Supplementary Table 13 indicates the measured CO_2RR efficiency of W_2C NFs at different cell potentials. The results indicate that a maximum efficiency of 62.3% is obtained at a cell potential of -1.7 V.

It has been demonstrated that considering the initial and the capital costs of industrially mature solid polymer electrolyte flow electrolyzer and relative prices of fossil fuels and electricity, a CO_2RR efficiency of approximately 40 to 50% is required to approach the commercially relevant electrocatalytic CO_2RR[50–52]. As shown in Supplementary Table 13, the efficiency of CO_2RR using W_2C NFs at the cell potential range between -1.6 to -2.1 is higher than 50% which demonstrates the process is economically feasible.

Supplementary Table 13 | CO_2RR Efficiency of W_2C NFs in the flow electrolyzer at different applied cell potentials.

Cell Potential (V)	***Power of Products (W.cm^{-2})***	***Cell Power Density (W.cm^{-2})***	***CO_2RR Efficiency (%)***
-1.6	0.09919	0.197724	50.16688
-1.7	0.17114	0.274601	62.32502
-1.8	0.22551	0.387158	58.24897
-1.9	0.27878	0.512895	54.35478
-2	0.34312	0.654256	52.44455
-2.1	0.41750	0.828035	50.42125
-2.2	0.45844	1.018791	44.99854
-2.3	0.50386	1.295007	38.90867

We also compared the maximum efficiency of W_2C NFs in the CO_2RR with state-of-the-art catalysts in the literature (Supplementary Figure 47)[53–57]. As shown in Supplementary Figure 47 W_2C NFs exhibits a maximum CO_2RR efficiency of 62.3% which is 1.67 and 1.72 times higher than that of Cu_{oh} and Cu-CIPH catalysts, respectively.

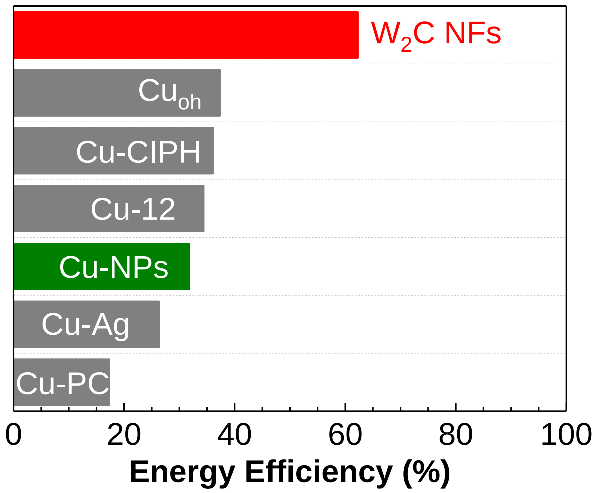

Supplementary Figure 47 | CO_2RR Efficiency of W_2C NFs compared to state-of-the-art catalysts

18- Electrochemical Performance of The Solar-Powered Flow Cell: Methods and Materials

For the standalone self-operating process, the electrochemical flow electrolyzer cell was connected to a triple junction photovoltaic (TJ-PV) solar cell to power the flow electrolyzer with solar energy. Supplementary Figure 48a indicates the unit of TJ-PV cell. A sun simulator light source was used to provide different sun illumination energies for the TJ-PV cell. Therefore, a preliminary procedure was used to calibrate different sun illumination distances. An InGaAs photodiode (Thorlabs, FDG03-CAL) with a known responsivity calibration curve was used to measure 0.5, 1, 1.5 and 2 sun illuminations energies. The TJ-PV cell was characterized by connecting the PV cell to the potentiostat (CH Instrument 660E). Supplementary Figure 48b shows j-V curves of the cell obtained at different sun illuminations. The efficiency of TJ-PV cell refers to the ability of solar panel to convert sunlight into electrical energy calculating by Equation 12 where the P_s and P_{mp} are the power of sunlight that hits the solar cell's surface (1 KW/m^2) and maximum power of solar cell, respectively.

$$Maximum\ TJ - PV\ cell\ efficiency = \frac{P_{mp}}{P_s} \times 100 = \frac{V_{mp} \times I_{mp}}{P_s} \times 100 \qquad \text{(Equation 12)}$$

The results show that at one sun illumination the TJ-PV cell generates a photocurrent density of 13.6 mA/cm^2, suggesting a maximum efficiency of 34.3 %.

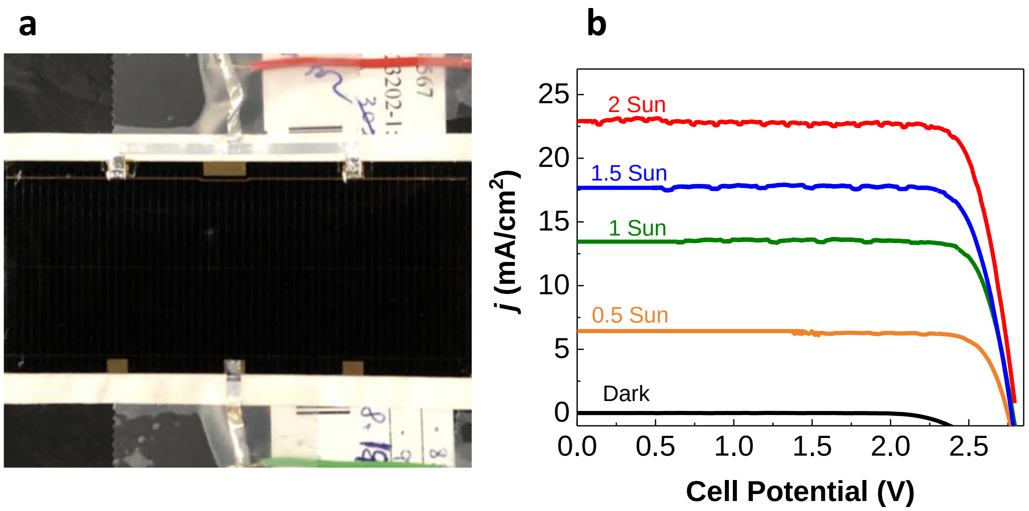

Supplementary Figure 48 | (a) A photo of TJ-PV cell and **(b)** Characterization at different sun illuminations

The flow electrolyzer was coupled to the TJ-PV cell with a surface area of 144 cm^2 to supply the required current density of -394.3 mA/cm^2 at a cell potential of -2.1 V for the flow electrolyzer with active surface area of 5 cm^2. Supplementary Figure 49 shows the j-V characteristic curve (blue curve) of the TJ-PV cell under one sun illumination (100 mW/cm^2). The maximum power point (MPP), shown by the orange dot in Supplementary Figure 49, indicates a photocurrent of 384.17 mA at a cell potential of 2.42 V. The red curve shown in this figure presents the measured current density of the solar-driven electrolyzer at different applied cell potentials. The operating point for the device is determined by the optimum point at which the two curves intersect (Supplementary Figure 49). The operating point is chosen to provide a photo current density of -394.3 mA/cm^2 at a potential of -2.1 V which has the maximum FE of CH_4 (82.7%±2%) calculated in the flow electrolyzer.

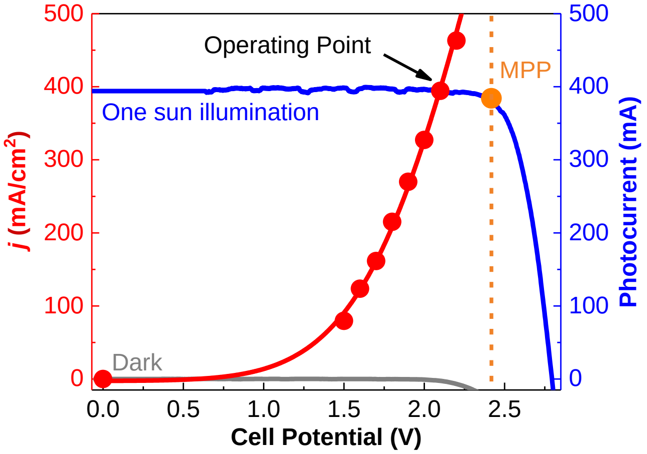

Supplementary Figure 49 | Characteristic curve (shown in blue) of the TJ-PV cell under one sun illumination. The red curve also shows the measured current densities at different cell potentials obtained by chronoamperometry experiments.

The measured current density of the flow electrolyzer using W_2C NFs at a cell potential of -2.1 V and characterization curves of TJ-PV cell, indicate that approximately ~29 cm^2 of the PV cell is needed to supply the required current for 1 cm^2 of the flow electrolyzer. The sun to given product efficiency of coupled cell is calculated by using Equation 13[58].

$$Sun\ to\ product = \frac{Power\ of\ product}{Solar\ power} = \frac{J_{op}\ FE_p(V_{op})LHV_P}{n_p F P_s} \qquad \text{(Equation 13)}$$

Where the operating current density J_{op} and the operating cell potential V_{op} are obtained by finding the intersection of the TJ-PV and the flow electrolyzer *j*-V curve. $FE_p(V_{op})$ is faradaic efficiency of product, which is function of cell potential. The LHV_P is the lower heating value per mole of product and P_s is the power of solar cell per unit area. The n_p is number of electrons transfer for the given product and F is faradaic constant which is 96485.33 $C.mol^{-1}$. All values are presented in Supplementary Table 14 [58]. The total sun to fuel efficiency was calculated by the sum of the sun to products efficiencies at the range of cell potentials.

$$Sun\ to\ fuels\ efficiency = \frac{J_{op}}{P_s} \sum \frac{FE_p(V_{op})LHV_P}{n_p F} \qquad \text{(Equation 14)}$$

Supplementary Table 14 | Number of electrons transfer, lower heating values, thermoneutral potentials, and equilibrium potentials for different reactions.

Reaction	*Number of Electrons*	*Equilibrium Potential (V)*	*Thermoneutral Potential (V)*	*Lower Heating Value (kJ.mol^{-1})*
$H_2O \rightarrow H_2 + ½ O_2$	2	1.229	1.481	241.81
$CO_2 \rightarrow CO + ½ O_2$	2	1.329	1.466	282.98
$CO_2 + 2H_2O \rightarrow CH_4 + 2O_2$	8	1.059	1.153	802.23
$2CO_2 + 2H_2O \rightarrow C_2H_4 + 3O_2$	12	1.149	1.219	1322.94
$2CO_2 + 4H_2O \rightarrow 2CH_3OH + 3O_2$	6	1.199	1.255	638.73
$2CO_2 + 3H_2O \rightarrow C_2H_5OH + 3O_2$	12	1.144	1.181	1235.45

We also evaluated the long-term performance of W_2C NFs in the solar-powered flow electrolyzer by measuring sun to fuels efficiency of the cell in a 700-hour experiment. Supplementary Figure 50 shows a photo and different compartments of the solar-powered flow electrolyzer experimental setup.

a

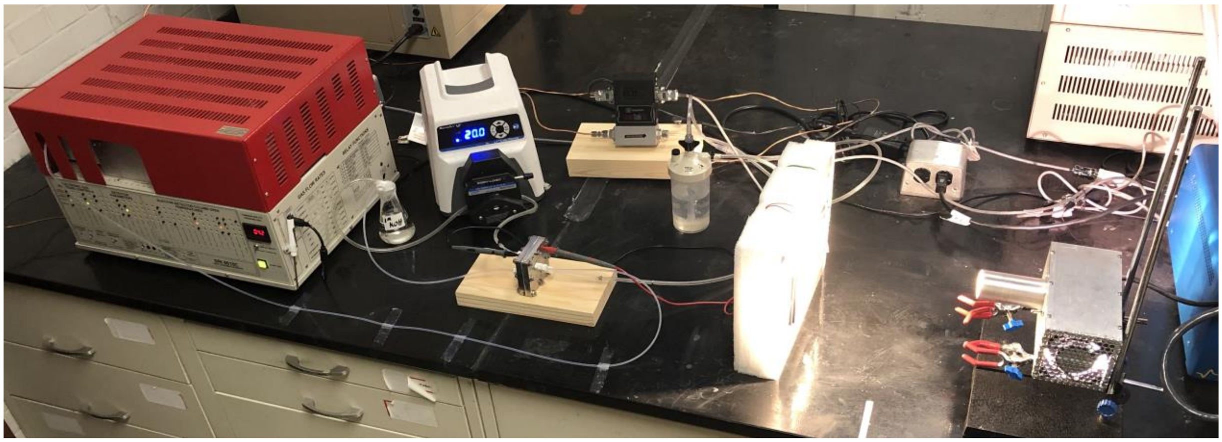

b

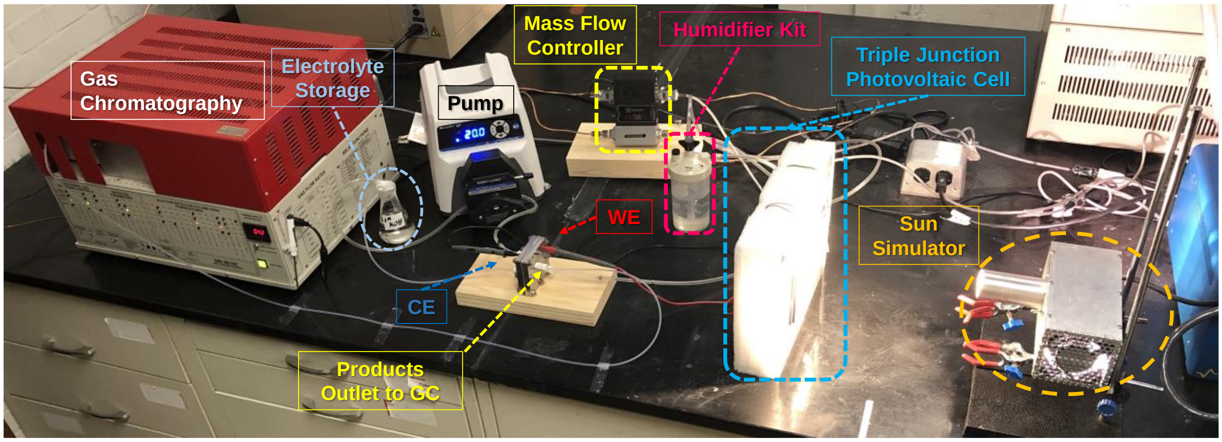

Supplementary Figure 50 | (a) a photo and **(b)** different compartment parts of experimental setup used for solar-driven electrochemical reduction of CO_2.s

Supplementary References

1. Guo, L. W. *et al.* Contributions of distinct gold species to catalytic reactivity for carbon monoxide oxidation. *Nat. Commun.* **7**, 13481 (2016).

2. Zhang, S. *et al.* Catalysis on singly dispersed bimetallic sites. *Nat. Commun.* **6**, 7938 (2015).

3. Murdoch, M. *et al.* The effect of gold loading and particle size on photocatalytic hydrogen production from ethanol over Au/TiO2 nanoparticles. *Nat. Chem.* **3**, 489 (2011).

4. Serpell, C. J., Cookson, J., Ozkaya, D. & Beer, P. D. Core@shell bimetallic nanoparticle synthesis via anion coordination. *Nat. Chem.* **3**, 478 (2011).

5. Esmaeilirad, M., Zabihi, M., Shayegan, J. & Khorasheh, F. Oxidation of toluene in humid air by metal oxides supported on Γ-alumina. *J. Hazard. Mater.* **333**, 293–307 (2017).

6. Esmaeilirad, M. *et al.* Oxygen Functionalized Copper Nanoparticles for Solar-Driven Conversion of Carbon Dioxide to Methane. *ACS Nano* **14**, 2099-2108 (2020).

7. Kondori, A. *et al.* Identifying Catalytic Active Sites of Trimolybdenum Phosphide (Mo_3P) for Electrochemical Hydrogen Evolution. *Adv. Energy Mater.* **9**, 1900516 (2019).

8. Asadi, M. *et al.* Cathode Based on Molybdenum Disulfide Nanoflakes for Lithium-Oxygen Batteries. *ACS Nano* **10**, 2167–2175 (2016).

9. Zhang, T. *et al.* Highly dispersed, single-site copper catalysts for the electroreduction of CO_2 to methane. *J. Electroanal. Chem.* 113862 (2020).

10. Möller, T. *et al.* Efficient CO_2 to CO electrolysis on solid Ni–N–C catalysts at industrial current densities. *Energy Environ. Sci.* **12**, 640–647 (2019).

11. Ren, S. *et al.* Molecular electrocatalysts can mediate fast, selective CO_2 reduction in a flow cell. *Science* **365**, 367 – 369 (2019).

12. Wu, J. *et al.* A metal-free electrocatalyst for carbon dioxide reduction to multi-carbon hydrocarbons and oxygenates. *Nat. Commun.* **7**, 13869 (2016).

13. De Gregorio, G. L. *et al.* Facet-Dependent Selectivity of Cu Catalysts in Electrochemical CO_2 Reduction at Commercially Viable Current Densities. *ACS Catal.* **10**, 4854–4862 (2020).

14. De Luna, P. *et al.* Catalyst electro-redeposition controls morphology and oxidation state for selective carbon dioxide reduction. *Nat. Catal.* **1**, 103–110 (2018).

15. Chen, S. *et al.* Highly Selective Carbon Dioxide Electroreduction on Structure-Evolved Copper Perovskite Oxide toward Methane Production. *ACS Catal.* **10**, 4640–4646 (2020).

16. Wang, X. *et al.* Efficient Methane Electrosynthesis Enabled by Tuning Local CO_2 Availability. *J. Am. Chem. Soc.* **142**, 3525–3531 (2020).

17. Weng, Z. *et al.* Active sites of copper-complex catalytic materials for electrochemical carbon dioxide reduction. *Nat. Commun.* **9**, 415 (2018).

18. Manthiram, K., Beberwyck, B. J. & Alivisatos, A. P. Enhanced Electrochemical Methanation of Carbon Dioxide with a Dispersible Nanoscale Copper Catalyst. *J. Am.*

Chem. Soc. **136**, 13319-13325 (2014).

19. Zhan, C. *et al.* Computational Screening of MXene Electrodes for Pseudocapacitive Energy Storage. *J. Phys. Chem. C* **123**, 315–321 (2019).

20. Tang, C., Wang, D., Wu, Z. & Duan, B. Tungsten carbide hollow microspheres as electrocatalyst and platinum support for hydrogen evolution reaction. *Int. J. Hydrogen Energy* **40**, 3229–3237 (2015).

21. Krasovskii, P. V *et al.* XPS study of surface chemistry of tungsten carbides nanopowders produced through DC thermal plasma/hydrogen annealing process. *Appl. Surf. Sci.* **339**, 46–54 (2015).

22. Ishii, T., Yamada, K., Osuga, N., Imashiro, Y. & Ozaki, J. Single-Step Synthesis of W_2C Nanoparticle-Dispersed Carbon Electrocatalysts for Hydrogen Evolution Reactions Utilizing Phosphate Groups on Carbon Edge Sites. *ACS Omega* **1**, 689–695 (2016).

23. Ba, K. *et al.* Single Faceted Two-Dimensional Mo_2C Electrocatalyst for Highly Efficient Nitrogen Fixation. *ACS Catal.* **10**, 7864–7870 (2020).

24. Yang, G., Zhao, J., Yi, S., Wan, X. & Tang, J. Biodegradable and photostable Nb_2C MXene quantum dots as promising nanofluorophores for metal ions sensing and fluorescence imaging. *Sensors Actuators B Chem.* **309**, 127735 (2020).

25. Cao, Y. *et al.* Engineered Exosome-Mediated Near-Infrared-II Region V_2C Quantum Dot Delivery for Nucleus-Target Low-Temperature Photothermal Therapy. *ACS Nano* **13**, 1499–1510 (2019).

26. Larson A. C., Von Dreele R. B., General Structure Analysis System (GSAS). Los Alamos National Laboratory, Los Alamos, New Mexico (1994).

27. Holzwarth, U. & Gibson, N. The Scherrer equation versus the Debye-Scherrer equation. *Nat. Nanotechnol.* **6**, 534 (2011).

28. Patterson, A. L. The Scherrer Formula for X-Ray Particle Size Determination. *Phys. Rev.* **56**, 978–982 (1939).

29. Kohn, W. & Sham, L. J. Self-Consistent Equations Including Exchange and Correlation Effects. *Phys. Rev.* **140**, A1133–A1138 (1965).

30. Hohenberg, P. & Kohn, W. Inhomogeneous Electron Gas. *Phys. Rev.* **136**, B864–B871 (1964).

31. Sánchez-Portal, D., Ordejón, P., Artacho, E. & Soler, J. M. Density-functional method for very large systems with LCAO basis sets. *Int. J. Quantum Chem.* **65**, 453–461 (1997).

32. Perdew, J. P., Burke, K. & Wang, Y. Generalized gradient approximation for the exchange-correlation hole of a many-electron system. *Phys. Rev. B* **54**, 16533–16539 (1996).

33. Rivero, P. *et al.* Systematic pseudopotentials from reference eigenvalue sets for DFT calculations: Pseudopotential files. *Data Br.* **3**, 21–23 (2015).

34. Monkhorst, H. J. & Pack, J. D. Special points for Brillouin-zone integrations. *Phys. Rev. B* **13**, 5188–5192 (1976).

35. Michaelson, H. B. The work function of the elements and its periodicity. *J. Appl. Phys.* **48**,

4729–4733 (1977).

36. Otani, M. & Sugino, O. First-principles calculations of charged surfaces and interfaces: A plane-wave nonrepeated slab approach. *Phys. Rev. B* **73**, 115407 (2006).

37. Kresse, G. & Hafner, J. Ab initio molecular-dynamics simulation of the liquid-metal--amorphous-semiconductor transition in germanium. *Phys. Rev. B* **49**, 14251–14269 (1994).

38. Kresse, G. & Furthmüller, J. Efficient iterative schemes for ab initio total-energy calculations using a plane-wave basis set. *Phys. Rev. B* **54**, 11169–11186 (1996).

39. Kresse, G. & Joubert, D. From ultrasoft pseudopotentials to the projector augmented-wave method. *Phys. Rev. B* **59**, 1758–1775 (1999).

40. Perdew, J. P., Burke, K. & Ernzerhof, M. Generalized Gradient Approximation Made Simple. *Phys. Rev. Lett.* **77**, 3865–3868 (1996).

41. Grimme, S., Antony, J., Ehrlich, S. & Krieg, H. A consistent and accurate ab initio parametrization of density functional dispersion correction (DFT-D) for the 94 elements H-Pu. *J. Chem. Phys.* **132**, 154104 (2010).

42. Cramer, C. J. *Essentials of computational chemistry: theories and models*. (John Wiley & Sons, 2013).

43. Nørskov, J. K. *et al.* Origin of the Overpotential for Oxygen Reduction at a Fuel-Cell Cathode. *J. Phys. Chem. B* **108**, 17886–17892 (2004).

44. Rossmeisl, J., Logadottir, A. & Nørskov, J. K. Electrolysis of water on (oxidized) metal surfaces. *Chem. Phys.* **319**, 178–184 (2005).

45. Peterson, A. A., Abild-Pedersen, F., Studt, F., Rossmeisl, J. & Nørskov, J. K. How copper catalyzes the electroreduction of carbon dioxide into hydrocarbon fuels. *Energy Environ. Sci.* **3**, 1311–1315 (2010).

46. Lim, D. H. *et al.* Carbon dioxide conversion into hydrocarbon fuels on defective graphene-supported Cu nanoparticles from first principles. *Nanoscale* **6**, 5087–5092 (2014).

47. Li, N. *et al.* Understanding of Electrochemical Mechanisms for CO_2 Capture and Conversion into Hydrocarbon Fuels in Transition-Metal Carbides (MXenes). *ACS Nano* **11**, 10825–10833 (2017).

48. Kim, S. K., Zhang, Y. J., Bergstrom, H., Michalsky, R. & Peterson, A. Understanding the Low-Overpotential Production of CH_4 from CO_2 on Mo_2C Catalysts. *ACS Catal.* **6**, 2003–2013 (2016).

49. Kortlever, R., Shen, J., Schouten, K. J. P., Calle-Vallejo, F. & Koper, M. T. M. Catalysts and Reaction Pathways for the Electrochemical Reduction of Carbon Dioxide. *J. Phys. Chem. Lett.* **6**, 4073–4082 (2015).

50. De Luna, P. *et al.* What would it take for renewably powered electrosynthesis to displace petrochemical processes? *Science* **364**, 3506 (2019).

51. Wang, Q., Chen, X., Jha, A. N. & Rogers, H. Natural gas from shale formation – The evolution, evidences and challenges of shale gas revolution in United States. *Renew. Sustain. Energy Rev.* **30**, 1–28 (2014).

52. Parag, Y. & Sovacool, B. K. Electricity market design for the prosumer era. *Nat. Energy* **1**, 16032 (2016).

53. Dinh, C. T. *et al.* CO_2 electroreduction to ethylene via hydroxide-mediated copper catalysis at an abrupt interface. *Science* **360**, 783 – 787 (2018).

54. García de Arquer, F. P. *et al.* CO_2 electrolysis to multicarabon products at activities greater than 1 A cm^{-2}. *Science* **367**, 661 – 666 (2020).

55. Ringe, S. *et al.* Understanding cation effects in electrochemical CO_2 reduction. *Energy Environ. Sci.* **12**, 3001–3014 (2019).

56. Hoang, T. T. H. *et al.* Nanoporous Copper–Silver Alloys by Additive-Controlled Electrodeposition for the Selective Electroreduction of CO_2 to Ethylene and Ethanol. *J. Am. Chem. Soc.* **140**, 5791–5797 (2018).

57. Li, F. *et al.* Molecular tuning of CO_2-to-ethylene conversion. *Nature* **577**, 509–513 (2020).

58. Singh, M. R., Clark, E. L. & Bell, A. T. Thermodynamic and achievable efficiencies for solar-driven electrochemical reduction of carbon dioxide to transportation fuels. *Proc. Natl. Aca. Sci.* (2015).
